# Proline catabolism is a key factor facilitating *Candida albicans* pathogenicity

**DOI:** 10.1371/journal.ppat.1011677

**Published:** 2023-11-02

**Authors:** Fitz Gerald S. Silao, Tong Jiang, Biborka Bereczky-Veress, Andreas Kühbacher, Kicki Ryman, Nathalie Uwamohoro, Sabrina Jenull, Filomena Nogueira, Meliza Ward, Thomas Lion, Constantin F. Urban, Steffen Rupp, Karl Kuchler, Changbin Chen, Christiane Peuckert, Per O. Ljungdahl

**Affiliations:** 1 Department of Molecular Biosciences, The Wenner-Gren Institute, Science for Life Laboratory, Stockholm University, Solna, Sweden; 2 Shanghai Institute of Immunity and Infection, Chinese Academy of Sciences, Shanghai, China; 3 Intravital Microscopy Facility, Department of Molecular Biosciences, The Wenner-Gren Institute, Stockholm University, Stockholm, Sweden; 4 Department of Molecular Biotechnology, Fraunhofer Institute for Interfacial Engineering and Biotechnology IGB, Stuttgart, Germany; 5 Clinical Microbiology and Umeå Centre for Microbial Research (UCMR), Umeå University Umeå, Sweden; 6 Medical University of Vienna, Max F. Perutz Laboratories GmbH, Department of Medical Biochemistry, Vienna, Austria; 7 Institute of Microbiology, Department of Pathobiology, University of Veterinary Medicine Vienna, Vienna, Austria; 8 St. Anna Kinderkrebsforschung e.V., Children’s Cancer Research Institute, Vienna, Austria; University of California Los Angeles David Geffen School of Medicine, UNITED STATES

## Abstract

*Candida albicans*, the primary etiology of human mycoses, is well-adapted to catabolize proline to obtain energy to initiate morphological switching (yeast to hyphal) and for growth. We report that *put1-/-* and *put2-/-* strains, carrying defective Proline UTilization genes, display remarkable proline sensitivity with *put2*-/- mutants being hypersensitive due to the accumulation of the toxic intermediate *pyrroline-5-carboxylate* (P5C), which inhibits mitochondrial respiration. The *put1-/*- and *put2-/-* mutations attenuate virulence in *Drosophila* and murine candidemia models and decrease survival in human neutrophils and whole blood. Using intravital 2-photon microscopy and label-free non-linear imaging, we visualized the initial stages of *C*. *albicans* cells infecting a kidney in real-time, directly deep in the tissue of a living mouse, and observed morphological switching of wildtype but not of *put2-/-* cells. Multiple members of the *Candida* species complex, including *C*. *auris*, are capable of using proline as a sole energy source. Our results indicate that a tailored proline metabolic network tuned to the mammalian host environment is a key feature of opportunistic fungal pathogens.

## Author summary

*Candida albicans* is listed as one of four “critical priority” fungal pathogens by the World Health Organization (WHO). Intriguingly, *C*. *albicans* is a natural commensal organism thriving in symbiosis with other components of the human microflora. Given the opportunistic character of *C*. *albicans*, it is important to understand how nutrient availability within host microenvironments contribute to the transition from commensal to pathogenic growth. Here, we report that proline is catabolized by *C*. *albicans* as an important energy source, a characteristic that is conserved among other pathogenic *Candida* species, including the multidrug resistant *C*. *glabrata* and *C*. *auris*. Using different infection models and applying a state-of-the-art intravital imaging technique to visualize *C*. *albicans* cells infecting kidneys in a living mouse, we observed that strains unable to utilize proline exhibit significantly reduced virulence and filamentous hyphal growth. Genetic dissection of the Proline UTilization (PUT) pathway and deciphering the control mechanisms governing proline use led to the discovery that proline is toxic to cells unable to catabolize this amino acid. Our findings provide novel insights implicating proline metabolism as a key determinant of pathogenic fungal growth.

## Introduction

Proline is the sole proteinogenic secondary amino acid. Its pyrrolidine ring gives it a distinctive role in protein architecture and dynamics. Proline plays an important role in energy generation, stress protection, signaling and redox balance of cells across multiple kingdoms [1–3]. Proline is the most abundant amino acid in the extracellular matrix (ECM); collagen, contains ~23% proline and hydroxyproline combined [4], and mucin, is about ~13% proline [5]. In some pathological states, such as cancer and sarcopenia, proline is released in significant quantities as a result of ECM degradation by extracellular proteases [6, 7], generating a proline pool that can be assimilated by human cells and the associated microbiome. In eukaryotes, proline catabolism occurs largely in the mitochondria, and its complete oxidation can generate ~30 equivalents of ATP, making proline an excellent source of energy [3, 8, 9].

*Candida* spp. are the major fungal commensals in humans with *Candida albicans* as the predominant species. *C*. *albicans* is an opportunistic pathogen capable of causing a spectrum of pathologies ranging from superficial mycoses to life-threatening systemic infections. As a pathogen, *C*. *albicans* must circumvent the host immune response and acquire nutrients to support the bioenergetic demands of infectious growth. Proline is a potent inducer of morphological switching in *C*. *albicans*, i.e., yeast-to-hyphal growth [10, 11]. The inducing properties of proline depends on its catabolism, which stimulates the well-characterized hyphal-inducing Ras1/cAMP/PKA pathway [10]. *C*. *albicans* strains that cannot metabolize proline exhibit defective hyphal growth and reduced survival within macrophages [10]. Consistently, strains lacking *GNP2*, encoding the primary proline permease, are unable to filament in the presence of proline and exhibit reduced survival when co-cultured with macrophages [12]. Most of the presumed knowledge regarding Proline UTilization (PUT) in fungi has been extrapolated from studies on the budding yeast *Saccharomyces cerevisiae* (reviewed in [13]). The regulatory mechanisms underlying PUT in *C*. *albicans* have not been well-characterized.

In eukaryotes the catabolic conversion of proline to glutamate is restricted to the mitochondria and is carried out by the concerted actions of proline dehydrogenase (PRODH; EC 1.5.5.2) and Δ^1^-pyrroline-5-carboxylate (P5C) dehydrogenase (P5CDH; EC 1.2.1.88) (reviewed in [1, 2, 14]). In *C*. *albicans* PRODH and P5CDH are encoded by *PUT1* (C5_02600W) and *PUT2* (C5_04880C), respectively [10]. Put1 uses flavin adenine dinucleotide (FAD) as a co-factor, oxidizing proline to generate P5C and FADH_2_. The electrons from FADH_2_ reduce membrane bound ubiquinone, co-enzyme Q, effectively linking proline oxidation to the mitochondrial electron transport chain (ETC) [1, 2, 14]. In mammals, PRODH is functionally and physically linked to ETC-Complex II (succinate dehydrogenase) [15]. P5C forms a non-enzymatic equilibrium with L-glutamate γ-semialdehyde (GSA), an equilibrium that is pH sensitive; P5C formation is favored with increasing pH [16]. Put2 catalyzes the oxidation of GSA to glutamate resulting in the reduction of NAD^+^ to NADH, which is oxidized in an energy conserving manner by NADH dehydrogenase (ETC-Complex I). Glutamate is subsequently converted to α-ketoglutarate (α-KG) by the NAD^+^-dependent glutamate dehydrogenase (GDH; EC 1.4.1.2) releasing ammonia and reduced NADH [17, 18]. In mammalian cells, GDH is localized in the mitochondria and performs an important anaplerotic function by directly feeding the TCA cycle with α-KG [19]. In *C*. *albicans*, GDH (Gdh2; C2_07900W) is cytosolic [17]. Gdh2 is the key ammonia-generating enzyme when *C*. *albicans* cells utilize amino acids for growth, and it is responsible for the observed alkalization of the extracellular milieu [17].

Previously, we reported that mitochondrial proline catabolism induces and energizes hyphal formation in *C*. *albicans* and that *C*. *albicans* cells depend on proline catabolism to evade macrophages [10]. Here, we show that multiple pathogenic *Candida* spp. are able to catabolize proline as the sole nitrogen and carbon (energy) source. Using *C*. *albicans* as a paradigm species, we have carried out a thorough characterization of PUT, focusing on the induction by proline and the bioenergetics of proline-driven virulence. Mitochondrial proline catabolism is tightly regulated to minimize the toxicity of the intermediate P5C, and PUT is required for virulence in systemic infection models and survival in human neutrophils and whole blood. Finally, using intravital microscopy, we visualized the initial stages of colonization of the kidney *in situ* in a living host and confirm the importance of PUT in the induction of filamentous growth during the early stages of infection.

## Results

### The ability to utilize proline as sole energy source is common to pathogenic *Candida* species

**Fig 1:**
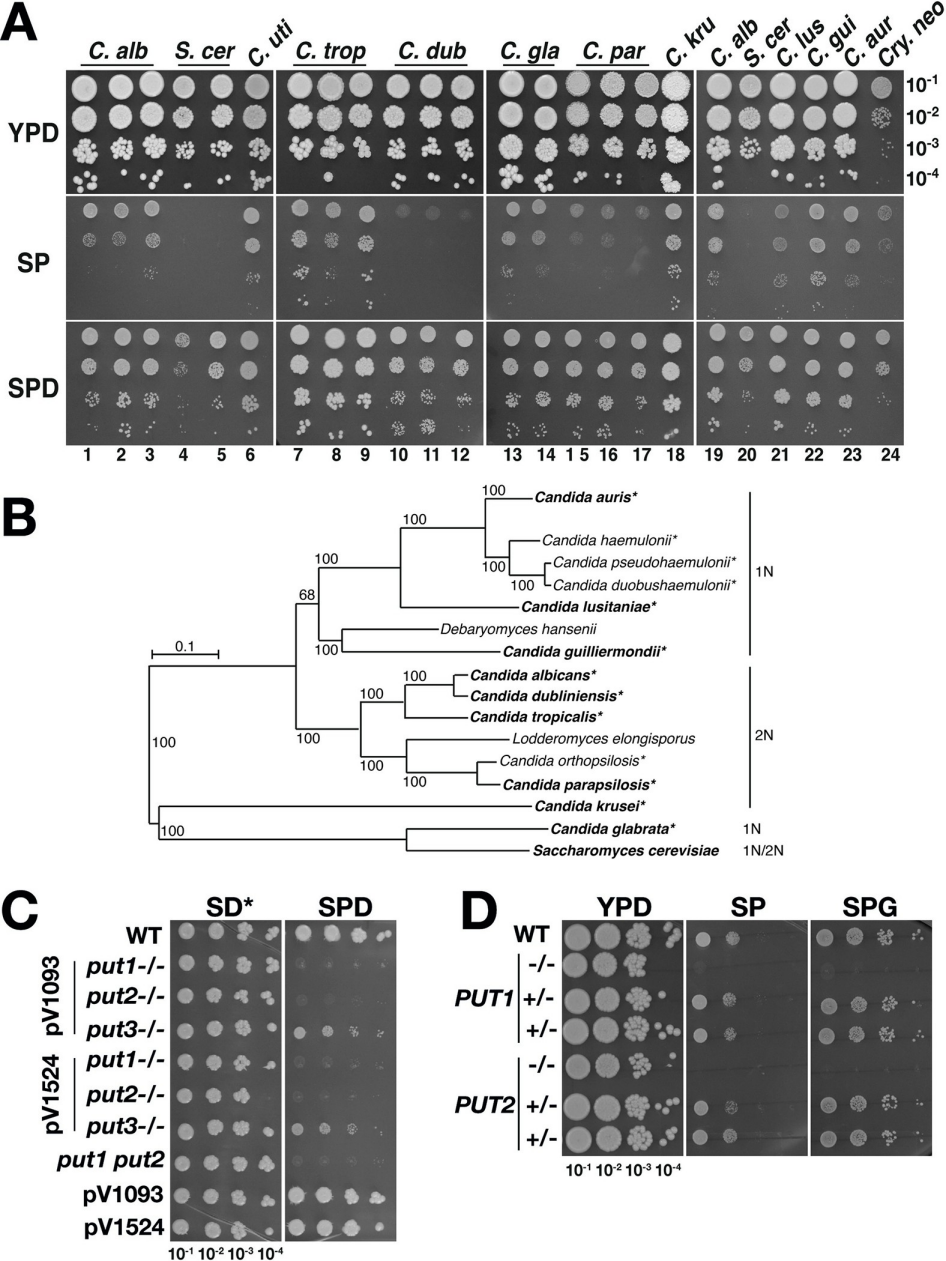
Proline as sole energy source supports growth of diverse pathogenic *Candida* species. (**A**) Proline utilization by members of the *Candida* pathogenic species complex. Serial dilutions of cells were spotted on YPD, SP (10 mM proline), and SPD (10 mM proline, 2% glucose) and then grown at 30°C for 48 h. Strains and dilution lanes: *C*. *albicans* (SC5314, PLC124 and MAY7), 1–3; *S*. *cerevisiae* (haploid S288c and diploid Σ1278b), 4–5; *C*. *utilis* (F608), 6; *C*. *tropicalis* [SM1541 and ATCC750 (from two laboratories)], 7–9; *C*. *dubliniensis* [SMI718 and Wü284 (from two laboratories)], 10–12; *C*. *glabrata* (CBS138 and Peu927), 13–14; *C*. *parapsilosis* (ATCC22019 from three laboratories), 15–17; *C*. *krusei* (ATCC6258), 18; *C*. *albicans* (SC5314), 19; *S*. *cerevisiae* (S288c), 20; *C*. *lusitaniae* (DSM 70102), 21; *C*. *guilliermondii* (ATCC6260), 22; *C*. *auris* (CFG552), 23; and *Cryptococcus neoformans* (NEQS), 24. (**B**) Phylogenetic tree of the *Candida* pathogenic species complex (adapted from [20]) with the indicated species ploidy state [haploid (1N) and diploid (2N)] and their capacity to cause infections in humans (*). (**C**) *PUT1* and *PUT2* are essential for proline utilization. Serial dilutions of cells were spotted on buffered (pH = 6) SD* and SPD and then grown at 30°C for 4 days. SD* contains 5 mM of ammonium sulfate compared to 38 mM in standard SD. Strains: WT (SC5314); pV1093-derivatives: *put1*-/- (CFG149), *put2*-/- (CFG143) and *put3*-/- (CFG150); pV1524-derivatives: *put1*-/- (CFG154), *put2*-/- (CFG318) and *put3*-/- (CFG156); *put1*-/- *put2*-/- (*put1 put2*, CFG159) derived using both pV1524 and pV1093; and control strains pV1093 (CFG181) and pV1524 (CFG182) carrying the vector without guide RNA. (**D**) Genetic reconstitution to assess the accuracy of CRISPR/Cas9 induced *put1-/-* and *put2-/-* mutations. The reconstituted strains [*PUT1*+/- (CFG379, CFG380); *PUT2*+/- (CFG381, CFG382)] generated by transforming *put1-/-* (CFG154) and *put2-/-* (CFG318) strains with their respective wildtype gene fragments (see S1 Fig for details) were grown on YPD, SP, and SPG as in (**C**).

*C*. *albicans*, as well as other *Candida* spp., have evolved in the low sugar environment of mammalian hosts and are rarely found living free in nature. Unlike the model yeast *S*. *cerevisiae*, *C*. *albicans* possesses mitochondria with a complete repertoire of electron transport complexes (ETC Complexes I-V) and is well-adapted to utilize proline as an energy source [10]. We examined the possibility that other fungi of the *Candida* pathogenic species complex [20, 21] have evolved the ability to exploit proline as an energy source (Fig 1). The laboratory *C*. *albicans* strain SC5314 (dilution 1) and two clinical isolates PLC124 (dilution 2) and MAY7 (dilution 3) grew on SP, a minimal synthetic medium containing 10 mM proline as sole source of nitrogen and carbon (Fig 1A). In contrast, the haploid S288c (dilution 4) and Σ1278b-derived diploid (dilution 5) *S*. *cerevisiae* strains did not grow on SP, but consistent with their ability to use proline as a nitrogen source, they did grow on SPD, which is SP supplemented with 2% glucose (Fig 1A). *S*. *cerevisiae* isolated from blood samples obtained from patients with fungal infections were also tested, and similar to the laboratory strains, they were unable to grow on SP.

Strikingly, many members of the *Candida* pathogenic species complex were found to utilize proline as a sole nitrogen and carbon source (Fig 1A). *C*. *dubliniensis*, a diploid yeast that is phylogenetically close to *C*. *albicans* (Fig 1B), exhibited poor growth on SP (dilutions 10–12), whereas *C*. *tropicalis* exhibited robust growth (dilutions 7–9). Remarkably, *C*. *glabrata*, a haploid yeast that is phylogenetically close to *S*. *cerevisiae* and thought to lack an energy-conserving mitochondrial NADH-dehydrogenase (ETC-CI; reviewed in [22]) grew on SP (dilutions 13–14). Other members of the complex, such as *C*. *parapsilosis* (dilutions 15–17), *C*. *lusitaniae* (dilution 21), *C*. *krusei* (dilution 18), *C*. *guilliermondii* (dilution 22), and the more recently characterized multi-drug resistant species *C. auris* [20] (dilution 23) exhibited growth on SP, albeit to varying degrees. Interestingly, the weaker growth of *C*. *dubliniensis* and *C*. *parapsilosis*, both diploid species like *C*. *albicans*, correlates with their being considered less virulent in nature [21, 23, 24]. *C*. *utilis*, a rare cause of human infection [25], also utilized proline efficiently, consistent with it possessing a functional mitochondrial ETC-CI (dilution 6) [26]. The data indicate that the ability to use proline as an energy source is a common attribute among pathogenic *Candida* spp., as well as in *Cryptococcus neoformans* (dilution 24), the latter a pathogenic yeast commonly found in HIV patients [27]. We included *C*. *neoformans*, although distantly related to *Candida* spp., since proline catabolism has been linked to its pathogenicity ([28], reviewed in [1]).

To establish the role of PUT in pathogenic growth, we considered *C*. *albicans* as a paradigm representative of the *Candida* spp. complex. Consistent to our previous report [10], independently generated *put1-/-* and *put2-/-* strains failed to grow on SPD where proline is utilized as sole nitrogen source (Fig 1C). Strains transformed with empty vectors (i.e., pV1093 [29] and pV1524 [30]) retained the ability to grow on SPD. To verify that the Put^-^ phenotypes were linked to modifications at the expected loci, wildtype *PUT1* or *PUT2* fragments were introduced into the *put1-/-* and *put2-/-* strains, respectively, and Put^+^ revertants were selected on SPD (S1A Fig). The heterozygous revertants regained the ability to grow on SP and SPG (SP+1% glycerol) where proline is used as an energy source (Fig 1D); restoration of the wildtype alleles was confirmed by PCR (S1B Fig). We also inactivated *PUT1* and *PUT2* in a *cph1*Δ/Δ *efg1*Δ/Δ non-filamenting *C*. *albicans* strain [31] by CRISPR and in *C*. *glabrata* using a modified *SAT1*-flipper technique [32] and the strains were similarly unable to grow on SPD (S1C Fig). Clearly, Put1 and Put2 are components of a non-redundant catabolic pathway that is essential for proline utilization. Throughout the remaining text, homozygous *put* mutants, i.e., *put1-/-*, *put2-/-* and *put3-/-* are designated as *put1*, *put2* and *put3*, respectively.

### Proline catabolism is induced by proline in a Put3-dependent and -independent manner

**Fig 2:**
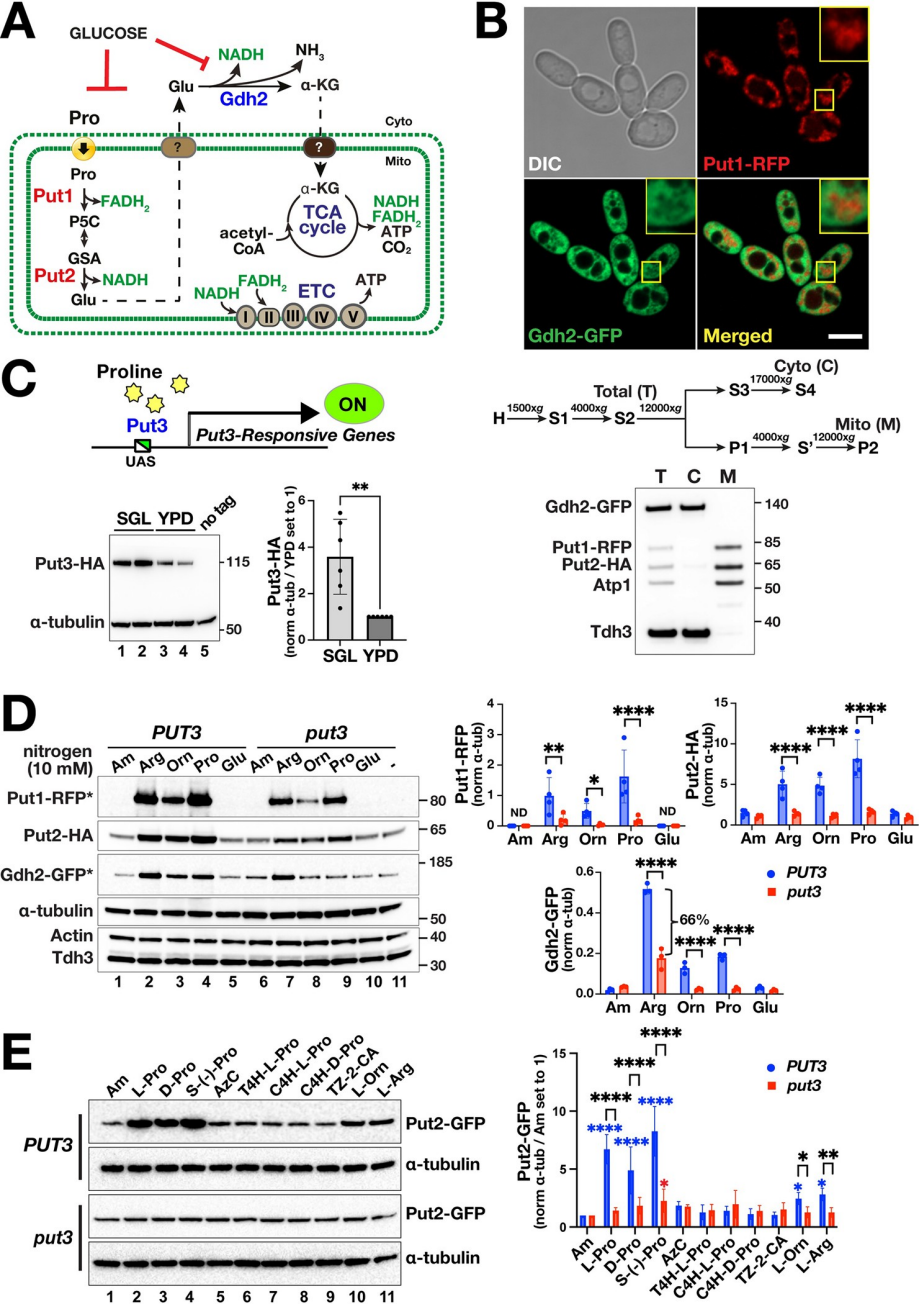
Proline catabolism is induced by proline in a Put3-dependent and -independent manner. (**A**) Schematic of proline catabolism in *C*. *albicans*. Proline is transported into mitochondria via unknown transporter and converted to glutamate in two enzymatic steps catalyzed by Put1 and Put2, which generates reduced electron carriers that are oxidized in the ETC forming ATP. Glutamate is deaminated in the cytoplasm by Gdh2. High glucose represses mitochondrial functions and Gdh2 expression. (**B**) Microscopy and subcellular fractionation of proline catabolic enzymes. (Upper panel) Representative confocal (Airyscan) image of strain CFG407 grown in YPG for 4 h at 37°C; (Lower panel) Subcellular fractionation analysis of strain CFG433 grown as in upper panel. Homogenized cell extracts (H) were fractionated according to the indicated centrifugation scheme [supernatant (S) and pellet fractions (P)]. The Total (T), cytosolic (C) and mitochondrial (M) fractions were analyzed by immunoblotting. Put1-RFP and Put2-HA co-fractionate with the mitochondrial marker (Atp1) while Gdh2-GFP co-fractionates with the cytosolic Tdh3. (**C**) Put3 expression is influenced by the growth medium. (Upper panel) Schematic diagram of proline-dependent activation of Put3-responsive genes. (Lower panel, left) Strains expressing Put3-HA (CFG187, lanes 1,3; CFG188, lanes 2,4) were grown to log phase in SGL and YPD as indicated and processed for immunoblotting. (Lower panel, right) Put3-HA signals normalized to α-tubulin and then to YPD set to 1 are presented (mean ± SD, n = 6; **p <0.01 by student *t*-test). (**D**) Put3-dependent and -independent induction of PUT enzymes. (Left panels) Immunoblot analysis of extracts prepared from *PUT3* (CFG441) and *put3* (CFG443) cells in SGL cultures 1 h after adding 10 mM of the indicated nitrogen sources. The Put1-RFP and Gdh2-GFP signals (*) were enhanced for display via the high slider in Image Lab, BioRad. (Right panels) Quantification of blots on the left. Indicated signals were normalized to α-tubulin as loading control (mean ± SD, n≥3; ****p <0.0001, **p <0.01, *p <0.05 by two-way ANOVA with Sidak’s post hoc test). Note that Put1-RFP is not detected (ND) in the absence of proline. (**E**) Put3 exhibits specificity for proline. (Left panels) Strains *PUT3* (CFG259) and *put3* (CFG301) were grown and processed as in (**D**) after adding 10 mM of the indicated compounds: Ammonium sulfate (Am); L-proline; D-proline; S-(-)-proline; Azetidine carboxylate (AzC); T-4-hydroxy-L-proline; C-4-hydroxy-L-proline, C-4-hydroxy-D-proline; Thiazolidine-2-carboxylic acid; L-ornithine; and L-arginine. Put2-GFP signals normalized to α-tubulin and then to Am set to 1 are shown (mean ± SD, n = 4) and were statistically treated as in (**D**). Black asterisks indicate the level of significance based on pairwise interstrain effects of each compound (*Put3* vs *put3*). Blue (*Put3*) and red (*put2*) asterisks indicate the level of significance based on intrastrain comparisons with each compound relative to Am as baseline; analyzed by one-way ANOVA with Dunnett’s multiple comparison test.

In *C*. *albicans*, as in *S*. *cerevisiae*, proline catabolism is induced by the presence of proline and, due to being mitochondrial localized, is sensitive to glucose repression (reviewed in [8]) (Fig 2A). In contrast to *S*. *cerevisiae*, *PUT1* and *PUT2* expression in *C*. *albicans* is not under nitrogen catabolite repression; their expression is induced by proline in a Put3-dependent manner even in cells grown in the presence of 38 mM ammonium [10, 33]. Proline is transported into mitochondria by an undefined process and the glutamate formed is directly or indirectly transported to the cytoplasm where it is deaminated to α-ketoglutarate in a reaction catalyzed by cytoplasmic glutamate dehydrogenase (Gdh2) [17, 18]. To investigate the mechanisms regulating proline catabolism, we created a reporter strain co-expressing Put1-RFP, Put2-HA, and Gdh2-GFP (S1D Fig), which enabled the simultaneous analysis of these three enzymes in a single lysate by immunoblot (see S1 Table for strains and S1 Fig for scheme of strain construction). Subcellular fractionation and microscopy confirmed the localization of the tagged constructs (Fig 2B). Consistent with our previous findings [17], Put1-RFP and Put2-HA localized to mitochondria and Gdh2-GFP localized to the cytoplasm.

Put3 is a transcription factor in *S*. *cerevisiae* that contains an N-terminal Zn (II)_2_Cys_6_ binuclear cluster DNA binding domain and a C-terminal domain that undergoes a proline-dependent conformational change that activates *PUT* gene expression [34, 35]. As in *S*. *cerevisiae*, the expression of these genes in *C*. *albicans* is positively regulated by Put3 (*PUT3*; C1_07020C) in response to proline [33]. However, inactivation of *PUT3* did not fully abolish the growth of *C*. *albicans* on SPD (Fig 1C)[10], indicating that the catabolic enzymes can be expressed independent of Put3. Put3-HA expression is not repressed by ammonium but is sensitive to glucose; Put3-HA is readily detected in cells grown in synthetic glycerol and lactate (SGL) medium containing glycerol and lactate as carbon sources and 38 mM ammonium sulfate as nitrogen source, whereas its expression is significantly lower in YPD containing 2% glucose (Fig 2C). Consistently, the *PUT3* promoter has two predicted Mig1/Mig2 binding motifs at -947 and -502 relative to the ORF (PathoYeastract [36]); Mig1 and Mig2 are partially redundant orthologues that mediate glucose repression in *C. albicans* [37].

We assessed the expression of Put1-RFP, Put2-HA, and Gdh2-GFP in *PUT3* (CFG441) and *put3* (CFG443) cells 1 h after different nitrogen sources (10 mM) were added to exponentially growing cultures in SGL (Fig 2D; also in S2A and S2B Fig). Put1-RFP was not detected in control cultures in the absence of additional nitrogen (-; lane 11) or with ammonium (Am; lane 1) (Fig 2D). In contrast and as reported [10], basal Put2-HA levels were expressed in both strains (lanes 1,11). All three reporters in *PUT3* cells were significantly induced upon the addition of 10 mM proline (lane 4). Arginine and ornithine, which can be metabolically converted to proline [10], also induced their expression (lanes 2,3). The lower induction of Put1-RFP in ornithine spiked cultures is likely due to the SPS-sensor dependency of its uptake (lane 3) [10]. Previous work, exploiting ChIP-Seq to identify Put3-regulated genes in *C*. *albicans*, did not identify *GDH2* as a main target [33]; however, these studies were conducted using cells grown in the presence of high glucose (YPD), a condition that we find represses the expression of *PUT3* (Fig 2C) and *GDH2* [17]. Interestingly, the levels of PUT enzymes including Gdh2 were induced by very low concentrations of proline (78 μM) (S2D Fig). Unexpectedly, the addition of glutamate or glutamine, which in mammals are precursors of proline biosynthesis [2, 14], did not induce Put1 expression (Figs 2D and S2A). In addition, the proline-dependent expression of Put1 occurred independent of Put2 (S2C Fig). Strikingly, proline induced the expression of Put1-RFP and Put2-HA in *put3* cells, albeit to a significantly lower level than in the *PUT3* strain (Fig 2D, lanes 4,9; S2B Fig). The low but significant Put3-independent expression of these enzymes is consistent with the ability of the *put3* strains to grow on SPD when proline merely serves as a nitrogen source (Fig 1C). Although the proline- or ornithine-dependent induction of Gdh2 was abolished in the *put3* strain, arginine still induced Gdh2 expression at a level ~66% of *PUT3* (Fig 2D; lanes 2,7). Apparently, additional proline- and arginine-sensitive factors contribute to the reporter expression.

Next, we tested the substrate specificity of Put3 by examining the capacity of proline and different proline analogs to induce Put2-GFP in *PUT3* (CFG259) and *put3* (CFG301) strains (Fig 2E). To avoid potential indirect effects of analog toxicity, we monitored the expression levels 1 h after addition of the compounds. The ability of Put3 to induce Put2 exhibited high specificity for L-proline, S-(-)-proline (a racemic pure form of L-proline) and D-proline (lanes 2–4), whereas none of the analogs (lanes 5–9), even the closely-related hydroxyproline compounds (lanes 6–8), induced expression. As previously observed (Fig 2D) and consistent with being catabolized to proline, the addition of ornithine and arginine resulted in the induction of Put2-GFP (lanes 10–11). Similar results were obtained using the triple tagged reporter *PUT3* (CFG441) and *put3* (CFG443) strains (S2B Fig).

### Proline is toxic in cells unable to catabolize it

**Fig 3:**
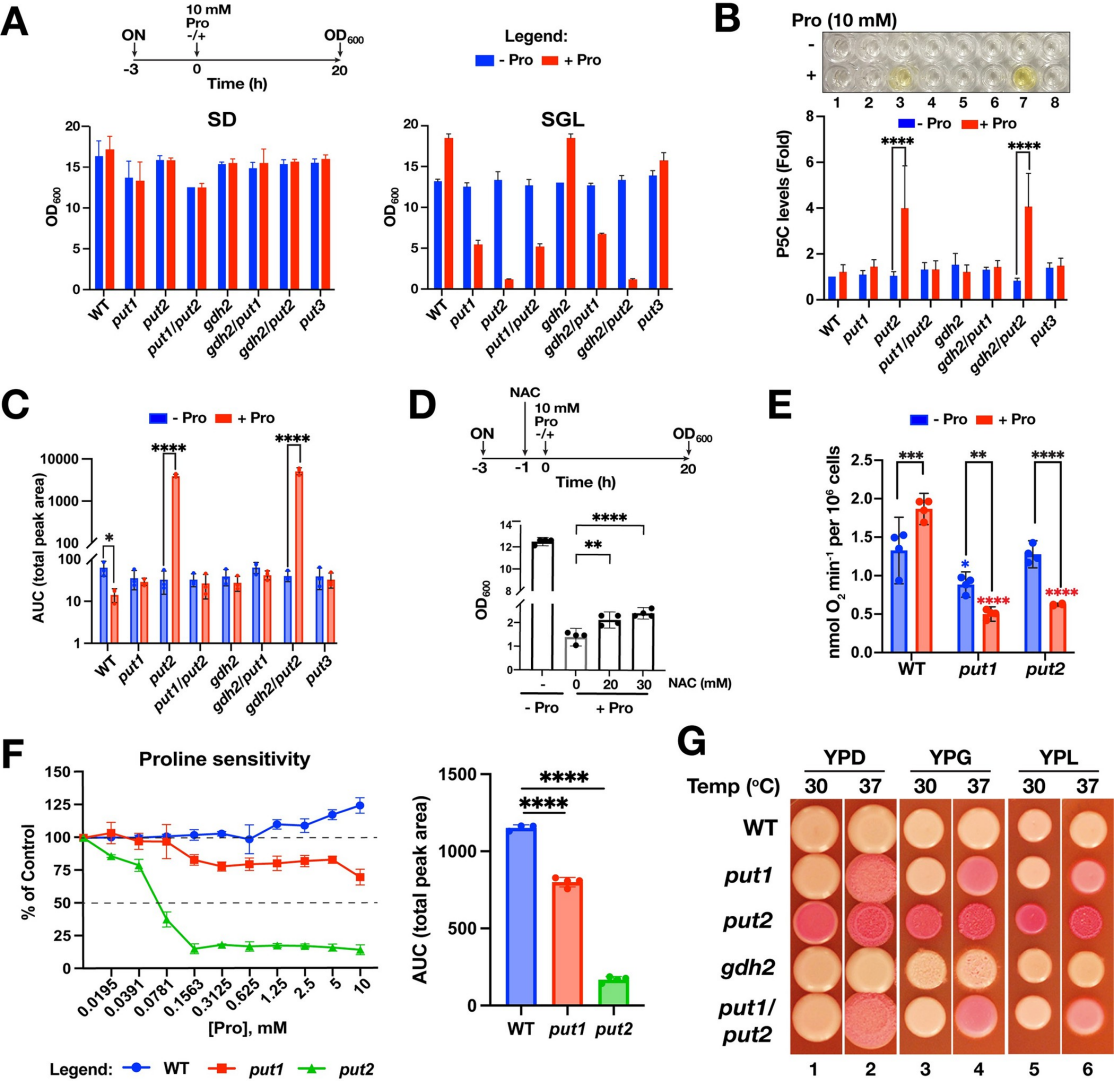
Proline is toxic in cells unable to catabolize it. (**A**) SD or SGL cultures of the indicated strains were treated with either 10 mM proline (red) or equal volume of H_2_O (blue), and then growth (OD_600_) measured after 20 h. Results presented as mean ± SD (n = 3). Scheme is on the top panel (see Methods for details). (**B**) Inactivation of *PUT2* leads to P5C accumulation in the presence of proline. P5C levels measured in the indicated mutants 2 h after treating without or with 10 mM proline. The data are presented as fold change of P5C levels relative to wildtype strain grown without proline set to 1 (mean ± SD; n = 5). (**C**) Inactivation of *PUT2* generates elevated ROS in the presence of proline. Strains were grown as in (**B**) but the levels of ROS determined 6 h after the addition proline (or ddH_2_O) using the luminol-HRP assay. The data are presented as area under the curve (AUC) (mean ± SD; n = 3). (**D**) Proline hypersensitivity of *put2* is largely independent of ROS accumulation. CFG318 grown as in (**A**) in SGL, and 1 h prior to proline addition cultures were diluted 1:1 with SGL containing freshly dissolved n-acetylcysteine (NAC) to the indicated concentration. Results presented as mean with 95% CI (n = 4). (**E**) Proline suppresses respiration in *put* mutants. Proline (10 mM; + Pro) or an equal volume of H_2_O (-Pro) was added to exponentially growing SGL cultures 4 h prior to measuring oxygen consumption (OCR). Data presented as mean with 95% CI (n = 4). (**F**) Cells carrying *put2* mutation are hypersensitive to submillimolar concentrations of proline. (Upper panel) Growth (as OD_600_) of the indicated strains in SGL with increasing concentrations of proline was measured after 24 h. Untreated control (set at 100%) and growth inhibition of 50% (IC_50_) are both shown as broken horizontal lines. (Lower panel) Area under the curve (AUC) obtained from each curve presented in the upper panel. All data shown are presented as mean ± SD (n≥3). (**G**) Proline catabolic mutants exhibit reduced vitality. Cells were spotted on the indicated medium containing the viability indicator Phloxine B and then incubated for 3 days at 30- or 37°C. Strains used in this figure: WT (SC5314); *put1* (CFG154); *put2* (CFG318); *put3* (CFG156); *put1 put2* (CFG159); *gdh2* (CFG279); *gdh2 put1* (CFG364); *gdh2 put2* (CFG366). Data presented were analyzed either by one-way (**D**, **E, F**) or two-way ANOVA (**A**, **B**, **C**, **D, E**) followed by post hoc test (****p<0.0001, ***p <0.001, **p <0.01, *p <0.05). For (**A**), all pairwise comparisons for SGL have *p*<0.0001 except *put3* (*p*<0.001). For (**E**), black asterisks indicate pairwise effect of proline addition while blue or red asterisks indicate interstrain effects on respiration relative to wildtype treated without (blue) or with (red) proline.

We considered the possibility that mutations inactivating proline catabolism could affect mitochondrial function and assessed growth under repressing (2% glucose; SD) and non-repressing (1% glycerol, 1% lactate; SGL) conditions. Under repressing condition, the mutants *put1*, *put2* and *gdh2* grew similarly regardless of whether 10 mM proline was present or not (Fig 3A, upper panel). However, when grown under non-repressing conditions (SGL), the addition of 10 mM proline resulted in striking growth inhibition of *put1* and *put2* cells (Fig 3A, lower panel). In the absence of proline, *put1* and *put2* mutants grew as wildtype, indicating that they are respiratory competent. These findings indicate that proline inhibits growth of cells that cannot catabolize it. Cells lacking Put2 exhibited extreme hypersensitivity to proline, only a minimal increase in OD_600_ was observed after 20 h. The proline hypersensitivity exhibited by *put2* mutant is partially rescued by the introduction of *put1* but not *gdh2* (Fig 3A), suggesting that the primary growth inhibitory effect of proline is linked to catabolic intermediates formed by Put1 and that are metabolized further by Put2, i.e., either P5C or GSA. Consistent with proline being an excellent energy source, the addition of proline enhanced the growth of the wildtype and *gdh2* strains. Aligned with our previous findings [10], the additive effect of proline on growth was more pronounced in non-repressing SGL than in repressing SD, clearly highlighting the effect of mitochondrial activity on proline utilization. Similar results were obtained on solid media (S3A Fig). The lack of proline inhibition of cells grown in high glucose media is linked to glucose repression of mitochondrial activity [10] (reviewed in [8]). Consistent with this notion, Put1 and Put2 protein levels remained elevated in wildtype cells 1 h after the addition of 10 mM proline in SGL and low in SD (S3B Fig). However, upon extended culture times (72 h), when glucose becomes limiting, the toxic effects of proline became apparent in *put2* cells, which gave rise to heterogenous colonies that varied greatly in size (S3C Fig). We further tested the inhibitory effects of proline in strains grown on synthetic SEM medium containing a non-repressing level of glucose (0.2%) and glutamate as nitrogen source (S3D Fig). In the absence of proline, all the strains formed macrocolonies surrounded by a halo of invasive hyphal cells. In the presence of proline (10 mM), the *put1* and *put2* strains exhibited slow growth and formed macrocolonies without hyphal halos. Consistent with our previous observations, the *put2* strain exhibited a more severe growth defect, and *put1* mutations are epistatic to *put2*.

### Proline inhibits growth of proline catabolic mutants

P5C is an unstable intermediate postulated to inhibit mitochondrial respiration and concomitantly to enhance ROS formation [38]. To test this notion, we used an improved protocol relying on complex formation between P5C and o-aminobenzaldehyde (o-AB) [39] to quantify P5C in *C*. *albicans* strains grown in SGL with and without proline. Initially, we had difficulty reproducibly measuring P5C; success was achieved by direct lysis of whole cells under acidic conditions (TCA) to stabilize P5C. The results confirmed that in the presence of proline, P5C levels are significantly elevated in *put2* but not in *put1 put2* double mutant cells; the level of P5C/o-AB complex is high enough in the proline-supplemented media to give a yellow tinge in the reaction solution (Fig 3B). Next, we measured ROS production using the luminol-HRP system. In the absence of proline, ROS levels were similar in all strains. However, upon proline addition, the ROS production increased dramatically in *put2* and *put2 gdh2* cells, but not in the *put1 put2* double mutant (Fig 3C). These results are consistent with Put1 acting upstream of Put2. The levels of ROS in *put2* cells treated with proline were significantly reduced in the presence of 10 mM N-acetylcysteine (NAC), a well-characterized ROS scavenger (S3E Fig). We grew cells in the presence of 2- and 3-fold higher doses of NAC to test whether the observed proline hypersensitivity of *put2* cells was coupled to increased ROS (Fig 3D). Although a significant dose-dependent increase in growth was observed in the presence of NAC relative to the control (- NAC), NAC failed to rescue the growth to a level comparable to cells grown in the absence of proline (Fig 3D). Likewise, cell permeable ROS scavengers like Mito-TEMPO [40] and TIRON [41] did not facilitate growth (S3F Fig). We conclude, as previously suggested [38], that the proline hypersensitive phenotype of *put2* is linked to a P5C-mediated respiratory block with ROS accumulation providing a secondary effect.

To test this notion directly, we measured oxygen consumption in *put1* and *put2* cells grown in the presence or absence proline. Relative to wildtype, the respiration rate of *put2* was reduced significantly when proline was present (Fig 3E). Interestingly, although Put1 levels were below the limit of detection in the absence of proline (Fig 2D), we observed a significant reduction in oxygen consumption relative to wildtype in *put1* without added proline, a rate that was further reduced in the presence of proline (Fig 3E). Using purified P5C, Nishimura et al. showed that *S*. *cerevisiae* is sensitive to P5C with an IC_50_ value of 23.8 μM [38]. We determined the IC_50_ of proline in *put2* cells in a microplate format and determined it to be 78 μM (Fig 3F); note that significant growth inhibition was already apparent at 39 μM proline (Figs 3F and S3G), a concentration that is 7-fold lower than the mean physiological level in human plasma (276 μM) [42]. At the apparent IC_50_ proline concentration (78 μM) Put1, Put2, and Gdh2 are robustly expressed in wildtype (S2D Fig). Growth of *put1* cells was clearly inhibited in the presence of 156 μM proline, however, the cells retained the ability to grow above 50% of the control even in the presence of 10 mM proline (i.e., IC_50_>10 mM) (Fig 3F, top panel). Quantification of growth inhibition, calculating the area under the curve (AUC), clearly shows the inhibitory effect of proline in *put* cells (Fig 3F, lower panel). Together our observations suggest that proline itself is growth inhibitory, and the capacity to metabolize proline is requisite to alleviate this effect.

### Defective proline utilization is linked to reduced vitality

Next, we assessed whether the growth inhibitory effects arising from defective proline catabolism affected the vitality of cells cultured in/on complex media with an abundance of competing nitrogen sources (amino acids, peptides), a scenario that better reflects conditions in a host. The term vitality reflects the overall physiological capabilities of cells [43], which can be impaired due to proline toxicity (Fig 3). Under repressing conditions of mitochondrial function (high 2% glucose) the *put* mutants grew similarly to wildtype (YPD; S4A and S4B Fig). By contrast, when grown under non-repressing conditions in/on YPG and YPL, containing non-fermentable carbon-sources glycerol and lactate, respectively, the *put2* strains exhibited poor growth (S4A and S4B Fig). In liquid YPG, the *put2* cells exhibited defects in cell separation forming trimera (S4A Fig, see inset), a phenotype associated with stress [44]. Notably, upon prolonged incubation on YPD (10 days), the colonies derived from cells carrying *put2* were distinctly yellow in appearance (S4C Fig). We posited that the yellow color was due to P5C accumulation, which is known to mediate cell death in multiple kingdoms [38, 45].

We used Phloxine B (PXB) to qualitatively assess cell vitality of cells in growing in macrocolonies. PXB crosses biological membranes and accumulates in cells that lack the metabolic energy to extrude it [43, 46]. Consequently, colonies with many metabolically inactive or dead cells accumulate PXB and take on a red appearance, the degree of redness reflects the number of impaired cells [47]. Macrocolonies formed by *put2* mutants accumulated significant amounts of PXB on YPD, YPG and YPL (Fig 3G). Although the *put1* and *put1 put2* mutants appear to grow as well as wildtype, we observed visible PXB uptake after 2–3 days in mature macrocolonies when grown at 37°C, whereas PXB is restricted to the colony periphery when grown at 30°C. PXB did not accumulate in the *gdh2* mutant on any of the media and temperatures tested (Fig 3G). We are aware that PXB uptake has been used as an indicator of the opaque phenotype in *C*. *albicans* strains carrying *efg1*Δ/Δ [48]. However, we observed PXB accumulation in Put deficient stains constructed in the non-filamenting *cph1*Δ/Δ *efg1*Δ/Δ background (S4D Fig), clearly indicating that PXB uptake is not exclusively linked to the opaque phenotype. Consistent with increased mitochondrial activity in low glucose, PXB uptake increased substantially in the *cph1*Δ/Δ *efg1*Δ/Δ *put2* strain grown in the presence of low glucose (compare YPD_0.2%_ vs standard YPD with +2% glucose) (S4D Fig; lane 1,3).

We attempted to obtain a more quantitative assessment of cell death using propidium iodide (PI) staining [43, 46]; however, we found that the *put* mutants flocculate in liquid culture, precluding measurements by FACS. The degree of flocculation, more pronounced in *put1* than *put2*, increased as the cultures became saturated (S4E and S4F Fig). The put^-^ cells grew normally until they entered the saturated phase where aggregation resulted in highly erratic measurements (S4G Fig). In yeast, flocculation is a known adaptation response to nutrient stress [49]. Consistent with this notion, the *put* mutants entering the phase have significantly lower levels of ATP than wildtype (S4H Fig). By microscopy, we observed an increased number of PI^+^ cells in 48 h-old YPD cultures of *put1* and *put2* but not *gdh2* strains (S4I Fig). These observations correlate with the relief of glucose-mediated repression of mitochondrial function, which facilitates proline toxicity, and as a consequence, the inability to generate sufficient energy to sustain life.

### Proline catabolism is required for invasive growth

**Fig 4:**
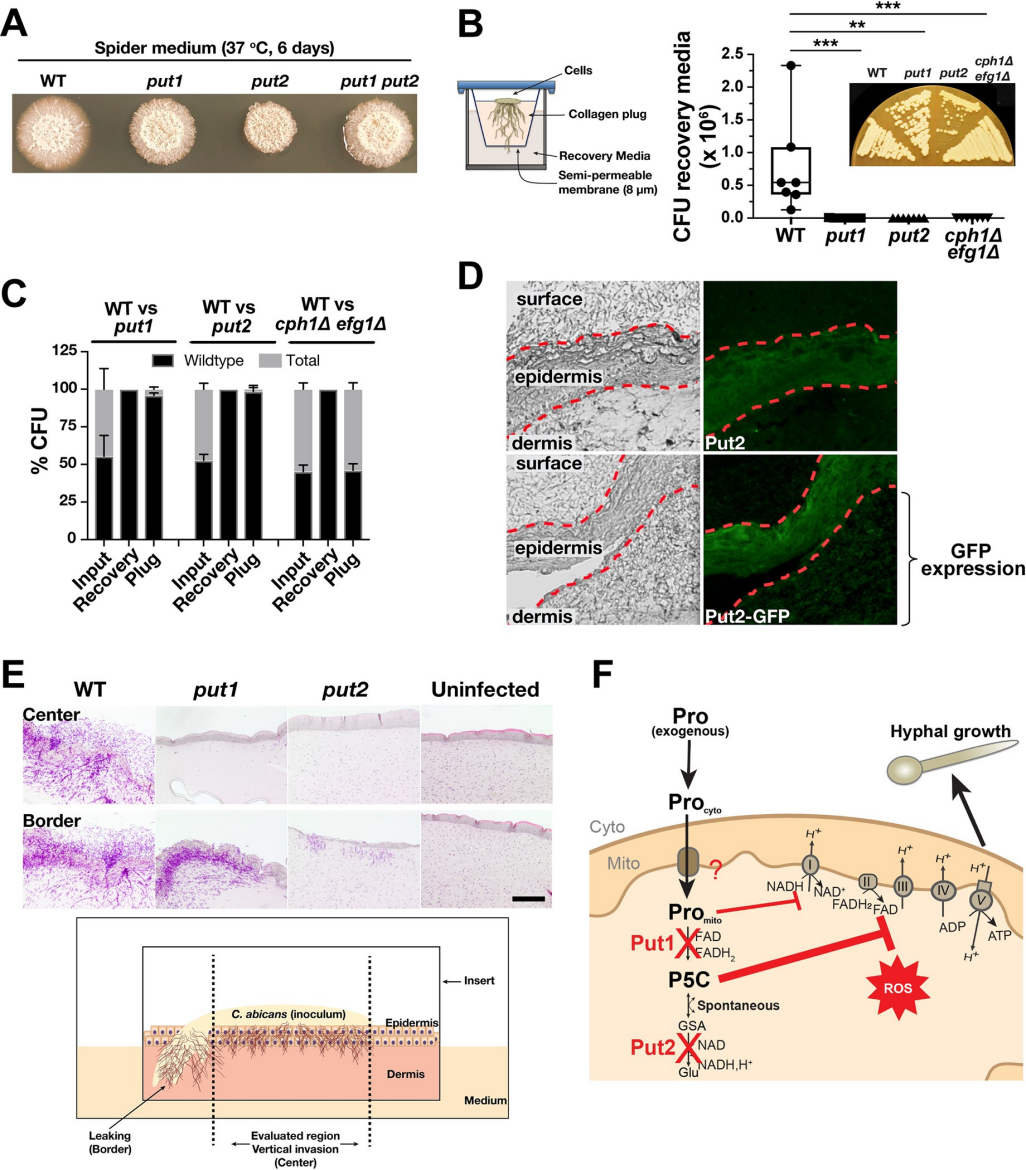
Proline catabolism is required for invasive growth. (**A**) Macrocolonies of the indicated strains grown on Spider medium for 6 days at 37°C. (**B**) Schematic diagram of the collagen invasion assay. Cells were added on the top of the collagen gel (PureCol EZ) in a transwell insert and were allowed to invade the plug for 14 days until they reach the recovery media which was analyzed for CFU (see Methods). Box and whiskers plot of CFUs derived from 7 biological replicates analyzed by Kruskal-Wallis test followed by Dunnett’s post hoc test (***p <0.001, **p<0.01). (Inset) Cells atop the collagen matrix after the 14-day growth were restreaked on YPD to determine cell viability. (**C**) The *put* mutants display reduced fitness compared to wildtype in a competitive collagen invasion assay. Cells as in (**B**) were mixed at a 50:50 ratio before adding on top of the collagen. Cells were recovered from input, recovery media and plug (see Methods). The strain genotypes were inferred by growth based-assays on SPD or Spider media to determine the WT:mutant ratio. (**D**) Proline catabolism is activated during invasive growth into reconstituted human skin. *PUT2* (PLC016) and *PUT2-GFP* (CFG219) cells were applied on the top of the stratified epidermal layer as indicated, and 2 days post infection, invasive growth was monitored by fluorescence microscopy. The dashed red lines demarcate the surface and dermal faces of the epidermal layer. Keratinocytes in the epidermal layer exhibit autofluorescence. Note the enhanced Put2-GFP signal in the epidermal and underlying dermal layers. (**E**) Proline catabolism is required for invasive growth through reconstituted human skin. (Upper panels) Periodic Acid-Schiff (PAS) staining of skin model 2 days after infecting with the indicated strains as in (**A**). (Lower panel) Schematic diagram of the infection model depicting the center and border areas. Compared to WT, *put1*and *put2* cells exhibit essentially a non-invasive phenotype in the center, similar to the uninfected control, and the greatly reduced capacity of *put2* cells to grow invasively is clearly evident. Strains used in figures **A**, **B**, **C**, or **E**: WT (SC5314), *put1* (CFG154), *put2* (CFG318), *put3* (CFG156), *put1 put2* (CFG159), and *cph1*Δ/Δ *efg1*Δ/Δ (CASJ041). **(F)** Schematic illustration of proline-mediated inhibition of *put1* and *put2* hyphal growth. The presence of exogenous proline from the environment is transported to the mitochondria where it either accumulate in *put1* or is converted to P5C in *put2* cells, respectively. Proline and P5C inhibit mitochondrial respiration suppressing hyphal growth. The high levels of ROS in *put2* is not the primary cause of poor growth (see Fig 3D).

We recently showed that *C*. *albicans* cells rely on proline catabolism to induce and energize hyphal growth in phagosomes of engulfing macrophages [10]. Relative to the wildtype, *put* mutants exhibit defects in producing long invasive hyphal filaments around the periphery of macrocolonies growing on Spider medium (Fig 4A), which contains mannitol as primary carbon source [50]. The diminished filamentous growth was more pronounced in *put2* (Fig 4A), which likely reflects the toxic effects of incomplete proline catabolism (S3D Fig). To further test the role of proline catabolism in powering invasive growth, we developed a collagen invasion assay (Fig 4B). Cells, applied on top of a collagen plug (Purecol EZ in DMEM/F12 medium) in a transwell, were monitored for their ability to induce filamentous growth, pass through the membrane (8 μm pore) at the bottom of the transwell and to reach the recovery medium (complete DMEM). The results were clear, the invasion process was dependent on the ability of cells to catabolize proline. In contrast to wildtype, the *put* mutants where recovered at extremely low levels (3 of 7 replicates had < 20 CFU) or even undetected (4 of 7 replicates with 0 CFU) in the recovery medium. As a control, a non-filamenting *cph1*Δ/Δ *efg1*Δ/Δ strain was used, and as expected, did not invade the collagen matrix. Next, we performed pairwise competition experiments mixing equal numbers of wildtype and mutant cells before applying them on the collagen plug (Fig 4C). At the end of the 14-day incubation period we quantified the total number cells in the plug and recovery medium, and determined the number of wildtype (WT) cells based on their ability to grow on SPD. For competition with *cph1*Δ/Δ *efg1*Δ/Δ control cells, we quantified wrinkled (WT phenotype) and smooth colonies (mutant phenotype) on Spider agar incubated at 37°C for 3–4 days. Starting with an input ratio of ~1:1 (WT:*put*), all cells in the recovery medium were wildtype and, in the collagen plug, the *put* mutants were overgrown by wildtype (Fig 4C). By contrast, *cph1*Δ/Δ *efg1*Δ/Δ cells, which are unable to form hyphae but capable of using proline, were recovered on the collagen plug almost at the same proportion as wildtype. The results indicate that proline catabolism is required by *C*. *albicans* to grow and invade collagen matrices.

To test these findings in a more complex and physiologically relevant assay system, we used an *in vitro* 3D skin model supplemented with functional immune cells as an infection platform [51]. First, we assessed whether proline is actively utilized by wildtype cells during the invasion process. To address this, we used a wildtype strain expressing Put2-GFP as a reporter strain (Fig 4D). Compared to the untagged Put2 strain that served to control for background fluorescence, significant GFP-dependent signal was observed in fungal cells invading the dermis layer. Consistent with the collagen invasion assay, the *put* mutants exhibited lower invasiveness compared to wildtype (Fig 4E) and filamenting fungal cells were not observed in the center of the skin model. However, *put1* but not *put2* cells filamented at the edge of the skin model, likely due to direct exposure to the filament inducing cell culture medium (see schematic of the model; Fig 4E). These results highlight the critical role of proline catabolism in supporting invasive growth of *C*. *albicans*. Proline and P5C accumulating in *put1* or *put2* mutant, respectively, inhibit mitochondrial respiration, which is essential for hyphal growth (Fig 4F).

We compared the ability of physiologically relevant sources of proline, in addition to collagen, e.g., serum albumin, mucin (from porcine stomach), and hemoglobin, to induce the expression of Put1 and Put2 (S5 Fig). Contrary to our expectation, collagen did not result in elevated expression of Put1, suggesting that the proline is not readily accessible to *C*. *albicans*. Interestingly, mucin, a major glycoprotein that lines mucosal membranes, e.g., the gut where *C*. *albicans* systemic infections can originate [52], robustly induced Put1 and Put2 expression. These results suggest that *C*. *albicans* play a more passive, rather than an active role, in liberating proline from proline-rich proteins, and that other factors, e.g., host processes contribute to the release of amino acids that subsequently become available for assimilation by fungal cells.

### *C*. *albicans* virulence is related to the ability to catabolize proline

**Fig 5:**
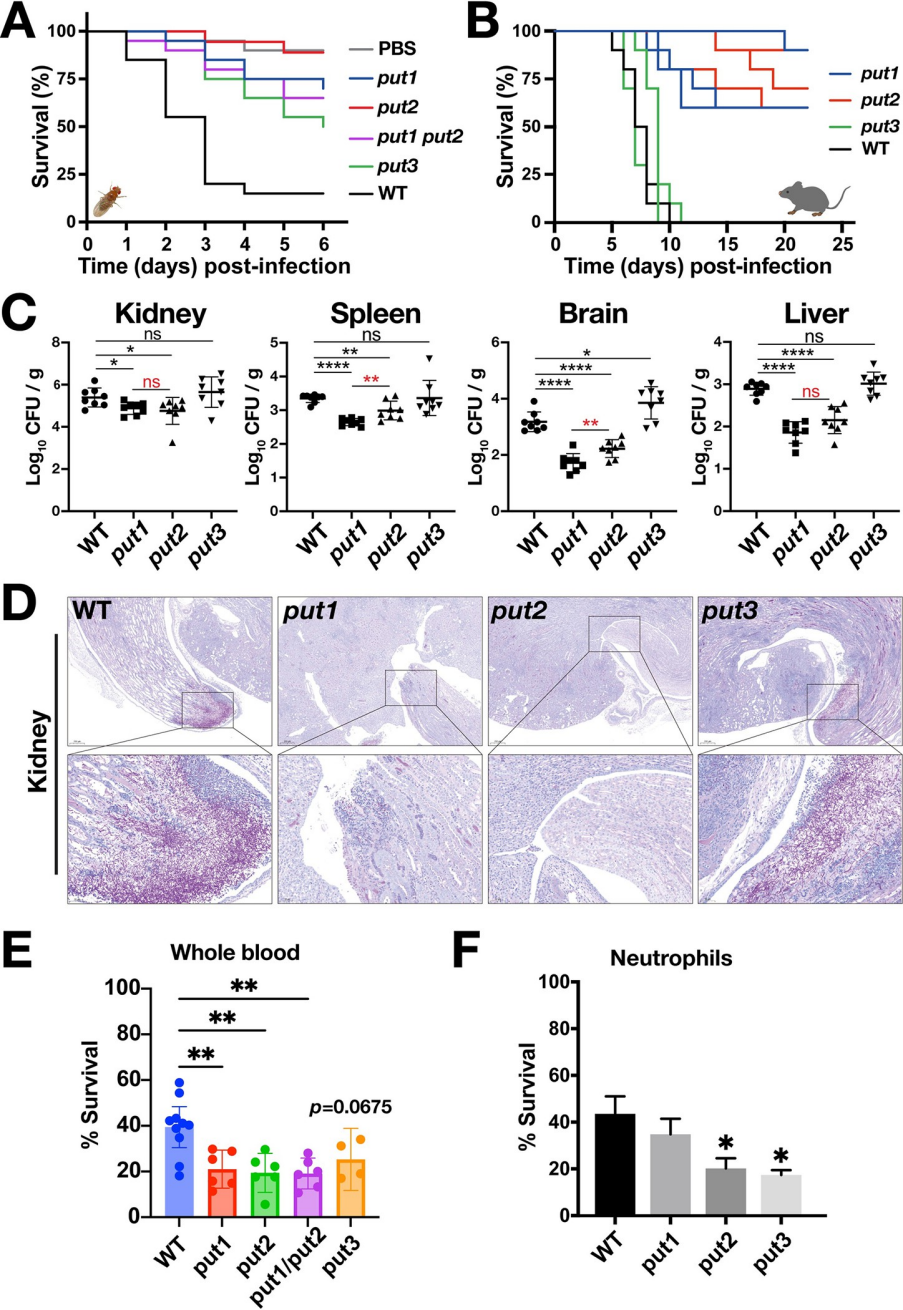
Proline catabolism is required for virulence in *Drosophila* and murine infection models. (**A**) *D*. *melanogaster Bom*^*Δ55C*^ flies were infected with WT and *put* mutants and their survival followed for 6 days. Each curve represents mean of at least 6 independent experiments performed on different days (****p<0.0001 by Log-rank (Mantel-Cox) test). There is no significance difference in the survival of *put1* and *put2* strains; statistical significance was evaluated by comparing the *p* values to the Bonferroni corrected α-value **(B)** Female C57BL/6 mice were intravenously infected with the indicated strains and their survival followed for 23 days. Data from 3 independent experiments performed on different days are shown. As there are 3 curves per strain, area under the curve (AUC) for each curve was determined and the AUCs analyzed by one-way ANOVA followed by Tukey’s post hoc test (WT vs. *put1*, ***; WT vs. *put2*, ****; WT vs. *put3*, ns; *put1* vs. *put2*, ns; *put1* vs. *put3*, ***; *put2* vs. *put3*, ****). **(C)** The fungal loads (CFU) of the indicated organs were determined 5 days post-infection. The results from three independent experiments (infected as in B) are plotted with each symbol represents an individual mouse. Data presented were analyzed either by one-way ANOVA followed by Dunnett’s post hoc test (black) or student *t*-test (red). **(D)** Kidneys of infected mice were removed at day 5 and then processed for PAS staining. Bars = 50 μm. **(E)** Put1 and Put2, but not Put3, are required for survival of *C*. *albicans* in a human blood infection model. Cells (~10^5^ CFU) were added into a blood aliquot and survival was assessed 1h after by plating. Data shown (mean with 95% CI) were obtained from 4–10 independent donors and analyzed by one-way ANOVA with Dunnett’s post hoc test (*p* = 0.0675 for *put3*). **(F)** Human neutrophils isolated from independent donors were co-cultured with WT and *put* mutants and fungal survival measured after 2 h. The data (mean ± SEM) were derived from 3 independent experiments and analyzed either by one-way ANOVA followed by Dunnett’s post hoc test. Strains used in the figure: WT (SC5314), *put1* (CFG154), *put2* (CFG318), *put3* (CFG156), and *put1 put2* (CFG159). Statistical significance: ****p<0.0001, ***p <0.001, **p <0.01, *p <0.05, ns = not significant.

The virulence of *put* strains was assessed in *Drosophila melanogaster* and mouse infection models, and two in vitro cell-based assays. First, an improved *Drosophila* mini-host model was used, exploiting *Bom*^*Δ55C*^ flies [53] that lack 10 *Bomanin* genes on chromosome 2 encoding secreted peptides with antimicrobial property [54]. As shown in the survival curves, the *put* mutants displayed greatly diminished virulence compared to wildtype (Fig 5A). Six days following infection, flies infected with the *put1*, *put3* or *put1*/*put2* showed general survival rates of approximately 70%, 50%, and 65%, respectively. In comparison, only 15% of the flies infected with wildtype survived. The median survival time (in days) for WT and *put3* were 3 and 6 days, respectively, while for *put1*, *put2*, *put1/put2* strains were all undefined since more than 50% of the subjects survived at the end of the study. Interestingly, the *put2* mutant exhibited a similar survival curve to that of PBS control, however, there is no significant difference compared to the survival of *put1* or *put1/put2* mutants (Fig 5A).

Next, C57BL/6 mice were systemically challenged with 10^5^ CFU of wildtype, *put1*, *put2* or *put3* strains. In comparison to wildtype, *put1* and *put2* strains exhibited significantly attenuated virulence (Fig 5B). The median survivals of *put1* and *put2* were undefined since >50% of subjects survived. Comparison of survival curves between these two showed no significant difference. On day 22 post infection, the mean survival rate of mice infected with *put1* or *put2* was 70% and 73.3%, respectively. In contrast to the fly model, the *put3* mutant did not exhibit attenuated virulence; there was no significant difference in the median survival in days (d) of wildtype (8.5d, 7.5d, 7d) and *put3* (9d, 7d, 7d) strains in three independent experiments. This suggests Put3-independent expression of *PUT1* and *PUT2* in mice (Fig 2D). Consistent to the longer survival times, the fungal burden 5 days after injection were significantly lower in the kidney, spleen, brain, and liver of *put1* and *put2* infected mice (Fig 5C). Localized comparison of fungal burden between *put1* and *put2* showed that in certain organs, such as spleen and brain, *put1* was significantly lower compared to *put2* (Fig 5C, red asterisks), whereas in the kidney and liver, there was no significant difference. Using Periodic Acid-Schiff (PAS)-staining, minimal filamentation was observed in the kidney sections of mice infected with *put1* relative to wildtype, and filamentation was virtually absent in the *put2* strain (Fig 5D). Interestingly, the kidney infected with *put3* showed smaller areas of infection compared to wildtype, but nonetheless the fungal burden was similar; a result that may be reconciled if the *put3* mutation results in a higher proportion of yeast-like rather than filamentous cells (Fig 5D).

To test whether proline catabolism is required for *C*. *albicans* survival in blood during systemic infection, we adopted a whole human blood infection model where *C*. *albicans* cells predominantly interact with neutrophils and to a lesser extent monocytes [55]. Neutrophils are the most abundant leukocytes in circulation, and play a critical role in controlling and clearing both mucosal and disseminated fungal infections. The regulated generation of ROS within the phagocytic compartment provides the major fungicidal mechanism [56]. The *put1*, *put2*, and *put1/put2* mutants exhibited significantly lower survival than the wildtype in this model (Fig 5E) suggesting that proline catabolism is required at the earliest response upon contact with blood. The survival of *put3* cells was also reduced (*p* = 0.0675). Next, we examined the survival in co-culture with isolated neutrophils. Consistently, neutrophils effectively reduced the survival of *C*. *albicans* cells, including wildtype (Fig 5F). The requirement for Put3 could be linked to other processes that are essential to survive the neutrophil response. Together, these results are consistent with our evidence that proline catabolism is rapidly activated in *C*. *albicans* during co-culture with neutrophils [57] and with a dual RNASeq study on whole blood that identified *PUT1* and *PUT2* as being rapidly upregulated (Log_2_FoldChange >1.5; 15- and 30-min post-infection) [55]. The consistent requirement for Put1 and Put2 in all infection models demonstrate that proline is actively assimilated *in situ* and that the capacity of *C*. *albicans* cells to utilize proline is a robust predictor of virulence.

### Visualizing acute *C*. *albicans* infections of the kidney in real time in a living host

The data from our murine virulence model (Fig 5B–5D) suggested that *C*. *albicans* cells require proline metabolism to infect kidneys during a systemic infection. Using a similar model of infection, Lionakis et al. showed that the kidney is the primary organ target of infection [58]. Subsequent work by the same group [59] suggested that compared to other organs, filamentation of *C*. *albicans* occurs remarkably fast in the kidney (within 2 h), implicating kidney-specific factors that drive the rapid filamentation. Notably, the kidney is a major hub for arginine and proline biosynthesis and is tightly connected to the metabolic activities in other organs [60–62]; arginine biosynthesis in the kidney is derived from citrulline produced from intestinal breakdown of glutamine [61]. To directly test the significance of proline metabolism and to obtain a detailed understanding of nutrient acquisition, we sought to follow the colonization of kidneys *in situ* in an intact living host. This was accomplished by visualizing *Candida*-host interactions using 2-photon intravital microscopy (IVM). Two-photon intravital microscopy (IVM) uses lasers to excite molecules in the near infrared range, which is more penetrating and less photo-damaging, making it suitable for live tissue imaging. Furthermore, only the molecules in the focal volume are excited, thus background signals are very low [63]. IVM has been successfully applied to study various disease states in live mice, including bacterial infection and cancer [64]. IVM provides resolution at the cellular level, and thus has significant advantages over other *in vivo* imaging methods that rely on bioluminescence to track infections in mice [65].

**Fig 6:**
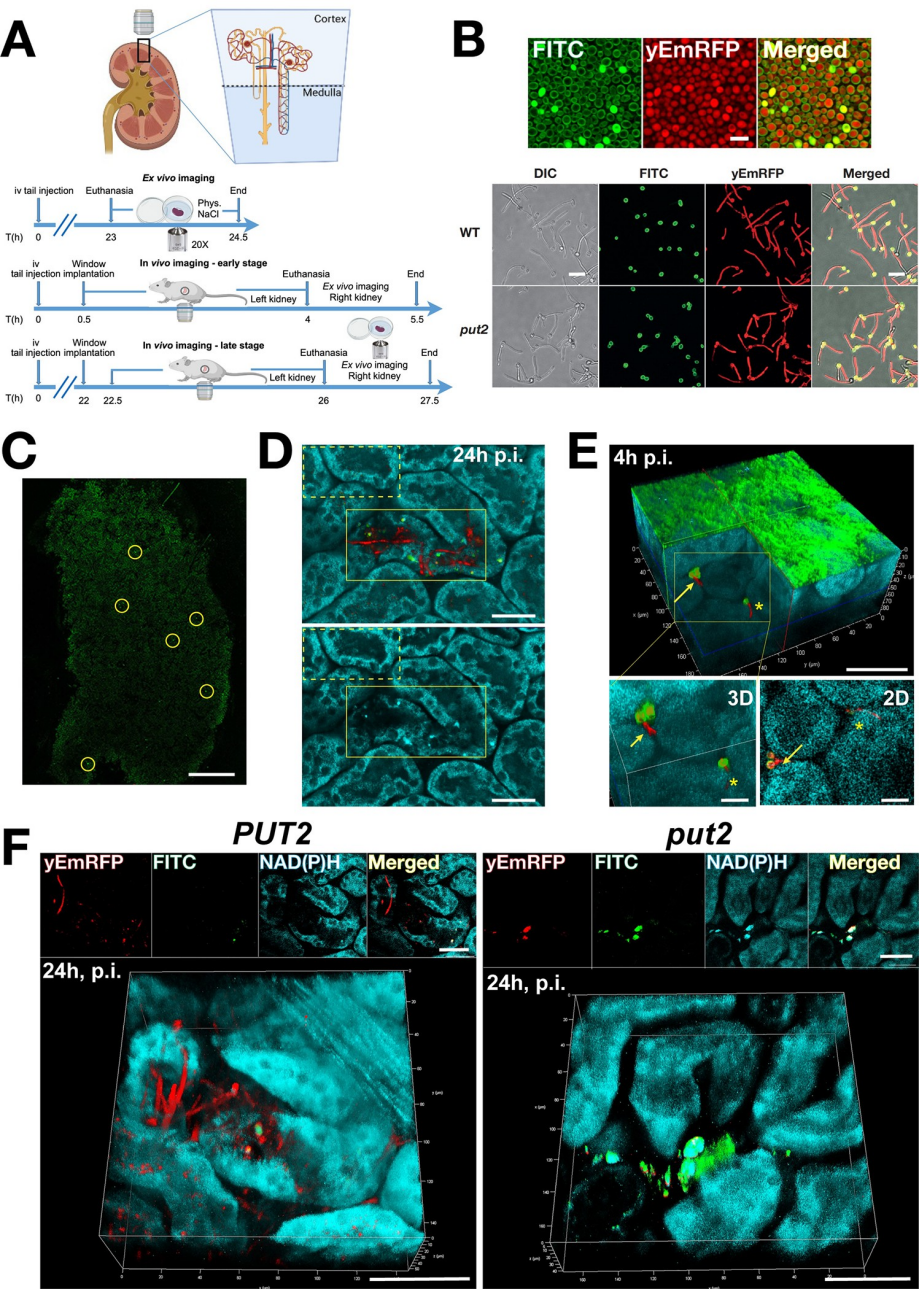
Intravital 2-photon microscopy imaging of acute *C*. *albicans* infections of the kidney in real time in a living host. **(A)** Schematic overview of the imaged area in the kidney and the experimental design for *ex vivo* and *in vivo* imaging, respectively. **(B, top)** A representative aliquot of *C*. *albicans* (PLC096) cells prepared for injection. The micrographs of FITC stained and yEmRFP expressing yeast-form cells were captured with the two-photon microscope used for IVM. Scale bar 10μm. **(B, bottom)** *C*. *albicans* wildtype (PLC096) and *put2* (CFG479) cells constitutively expressing yEmRFP and stained with FITC form hyphae 2h after induction in DMEM SILAC medium supplemented as required to support auxotrophies. FITC fluorescence remains exclusively associated with the mother cells. Scale bar, 20μm. **(C)** Sites of colonization where localized using a spiral scan in the Las-X Navigator-module in the FITC channel. The entire area of the renal surface attached to the glass imaging window was scanned; circles highlight examples of regions of interest (ROI) exhibiting stronger and deviating fluorescence from the background. Each ROI was examined in detail using FITC, yEmRFP and autofluorescence. Scale bar, 500 μm. **(D)** Single plane images from a z-stack of *ex vivo* imaged renal cortex 24 h p.i. with wildtype (PLC096). Upper panel, overlay of 3 channels is shown: yEmRFP (red), FITC (green), autofluorescence of NAD(P)H (teal). Note the FITC-positive fungal mother cells with long extended hyphal filaments (yEmRFP only) growing through and inside tubules. Lower panel, autofluorescence only, allows comparison of the morphologies of renal cells in non-infected (yellow box, dotted line) with infected (yellow box, solid line) areas. Note: the morphology of tubules in the infected area appears disrupted. Scale bar 40μm. **(E)** 3D reconstruction of an intravitally acquired z-stack through 90 μm of the renal cortex of a BALB/cAnNCrl mouse, 4 h p.i. with wildtype (PLC096). Upper panel, the 3D reconstruction was virtually clipped to expose the intertubular localized fungal cells. Overlay of 3 channels is shown: yEmRFP (red), FITC (green), autofluorescence of NAD(P)H (teal). Arrow points to a mother cell (yEmRFP and FITC-positive) with an emerging hypha (yEmRFP positive). The star marks a filament that is penetrating a tubular epithelial cell. The collagen associated with the renal capsule exhibits strong green autofluorescence. Scale bar, 50 μm. Lower panels, higher magnification of the area marked by yellow box in 3D (left) and a representative single 2D plane (right). Scale bars, 20 μm. **(F)** 3D reconstructions of intravitally acquired z-stacks through 40–50 μm of the renal cortex of BALB/cByJ SOPF mice, 24 h p.i. with wildtype *PUT2* (PLC096) (Left) and *put2* (CFG479) (Right) strains constitutively expressing yEmRFP. Panels on top of each stack show single 2D plane images for the individual channels and merged. Scale bar, 50 μm. (Left) *PUT2* mother cells (yEmRFP and FITC positive) with multiple long filaments (yEmRFP positive) were observed growing through and inside tubules (50% of kidneys had ≥1 infection site exhibiting hyphal growth, n = 10 mice). (Right) *put2* mother cells (yEmRFP and FITC positive) accumulated solely in yeast form and did not divide (0% of kidneys exhibited hyphal growth, n = 6 mice). The cartoons (Fig 6A) were created with Biorender.com and reproduced here under academic license (POL).

Optical access to the kidney was achieved by the implantation of an abdominal imaging window (Fig 6A). To observe fungal cells, we utilized *C*. *albicans* wildtype and *put2* strains constitutively expressing a modified version of mCherry (yeast enhanced mRFP, yEmRFP) placed under the control of the strong *ADH1* promoter. The *put2* strain was derived by inactivating *PUT*2 directly in the yEmRFP-expressing wildtype strain using CRISPR/Cas9. To monitor the growth characteristics of these strains, we stained yeast-form cells with FITC, which reacts with primary amines on the cell surface, and monitored the induction of filamentous growth in DMEM SILAC medium (Fig 6B). This medium, normally prepared without arginine and lysine, is routinely used for isotope labeling, however, here, because the yEmRFP expressing strains are derived in an arg-, his-, ura- background, we supplemented the medium as required, using a non-repressing quantity of arginine (10 μM). In this medium, both wildtype and *put2* cells readily filament (Fig 6B); yEmRFP expressing daughter cells emerge from FITC-stained mother cells. It should be noted that DMEM medium, normally prepared with 10% FBS (D10), contains significant amount of arginine/proline (DMEM has 400 μM arginine), which inhibits growth of *put2* cells (Figs 3F and S7).

We then injected *C*. *albicans* wildtype to induce systemic infections in living BALB/cAnNCrl mice; cells prepared for injection were imaged with same settings as for IVM at the two-photon microscope (Fig 6B, upper panels). To locate sites of colonization, the entire area of the renal surface attached to the glass of the imaging window was rapidly scanned at high speed and low resolution to identify putative sites of infection (Regions of Interest; ROI (Fig 6C; overview of FITC channel). Each ROI was examined at high resolution for fungal cell growth using FITC and yEmRFP fluorescence and autofluorescence We started with *ex vivo* imaging of acute excised kidneys at 24 h post injection with wildtype *C*. *albicans*, which helped us to establish the time frame for colonization (Fig 6D). In addition to FITC and yEmRFP fluorescence, the autofluorescence of endogenous fluorophores, such as NAD(P)H and collagen, was captured and used for reconstructing tissue morphology. At 24 h post injection, the kidneys displayed loci with heavy filamentation with hyphae growing inside and through different tubules (Fig 6D, upper panel). As apparent from the NAD(P)H-linked autofluorescence, the cellular morphology of the affected tubules was disintegrated as a consequence the growing filaments (Fig 6D, lower panel). Subsequently, we performed IVM immediately after injection with FITC stained wildtype *C*. *albicans* and imaging window implantation. We were able to visualize the renal cortex through the imaging window and reached the superficial areas consisting of proximal and distal convoluting tubules and peritubular arterioles. As early as 4 h post injection, we detected the initiation of filamentous growth of fungal cells in the renal cortex and in the intertubular space, most likely with capillary or interstitial localization (Fig 6E, upper panel). Daughter cells, expressing yEmRFP but lacking FITC-staining, grew forming germ tubes that initiate hyphal growth and started penetrating epithelial tubule cells (Fig 6E, lower panels).

To compare the virulence of *C*. *albicans* wildtype and *put2* strains, we injected two groups of specific and opportunistic pathogen free (SOPF) BALB/cByJ mice. The SOPF health status proved to be important to minimize individual variation in host responses. At 24 h post injection, between 3 and 15 ROI/mouse injected with wildtype (mean = 9.6 ROI/mouse, n = 10) and between 10 and 17 ROI/mouse injected with *put2* (mean = 13 ROI/mouse, n = 6) were imaged. We found foci with filamentous growth in 50% of the mice injected with the wildtype strain (Fig 6F, left). In contrast, no filamentous growth was observed in mice injected with the *put2* strain. The *put2* cells were found in intertubular locations and were positive for both, yEmRFP and FITC with no sign of filamentation (Fig 6F right; see also supplementary videos S1–S4 Movies). The lack of filamentation and proliferation of the *put2* strain in the kidney is consistent with the reduced virulence of this strain compared to wildtype in BALB/c mice (S6 Fig) and is comparable to the results obtained in C57BL/6 mice (Fig 5B). These observations suggest that proline is accessible in the kidney during the early stages of a kidney infection in a mouse model of systemic candidiasis.

## Discussion

The results documented here advance our understanding regarding the importance of proline catabolism in fungal infections in four ways. First, many *Candida* spp. known to cause mycoses in humans, including the recently described and multidrug resistant *C*. *auris*, have the capacity to catabolize proline as a sole nitrogen and carbon (energy) source. Second, the regulatory mechanisms controlling proline utilization are tightly coupled to mitochondrial function. Third, under respiratory conditions, proline is toxic to cells that lack the ability to catabolize it. This unexpected finding indicates that proline, in addition to the already known toxic intermediate P5C, negatively affects mitochondrial activity. The underlying cause remains to be elucidated; however, it seems likely that when proline accumulates above a critical threshold in mitochondria, it inhibits the activity of a critical ETC component. Fourth, our success in visualizing early events of *C*. *albicans* cells infecting the kidneys of a living host indicate that single cells respond to nutrient cues *in situ*, inducing invasive filamentous growth in a manner dependent on their ability to catabolize proline.

Despite proline being one of the first characterized inducers of morphogenic switching in *C. albicans* [11], we only recently discovered that *C*. *albicans* can catabolize proline as a preferred energy source [10]. The lag in appreciating the physiological significance of proline stems from the over reliance on information obtained in studies with the yeast *S*. *cerevisiae* (reviewed in [13]). Yeast cells are able to use proline as a nitrogen source but not as a sole energy source (Fig 1A) [66]. Accumulating evidence indicates that key regulatory differences exist between *C*. *albicans* and *S*. *cerevisiae*, which likely reflect the microenvironments in which they evolved (reviewed in [8]). *S*. *cerevisiae* is widely found in nature, whereas *C*. *albicans* and other members of the *Candida* spp. complex evolved growing in symbiosis with human hosts and are rarely found living outside of mammalian hosts. The finding that multiple *Candida* spp. can catabolize proline as the sole energy source is striking (Fig 1A). Although the PUT pathways are predicted to be conserved among these species, there were clear differences in how well proline is used. It is intriguing that the ability to use proline correlated with virulence characteristics of the *Candida* species, exemplified by comparing the growth of the two closely related species *C*. *albicans* and *C*. *dubliniensis*, *C*. *albicans* considered to be more virulent [67]. Interestingly, several human pathogens also utilize proline, including prokaryotes, e.g., *Helicobacter pylori*, and eukaryotes, e.g., *Trypanosomes* and *Cryptococcus neoformans* (reviewed in [1]). Future efforts focused on understanding species-specific regulatory differences will be helpful to elucidate the full significance of proline catabolism in pathogenic process.

The regulation of PUT in *C*. *albicans* is complex. Consistent with Tebung et al. [33], we found that proline induces the expression of Put1 and Put2 in a Put3-dependent manner (Fig 2D). However, in contrast, we observed that our *put3* strains exhibit residual growth on proline medium (Fig 1C), which correlates with the observed levels of Put1 and Put2 expression (Fig 2D). We found that Gdh2 expression, similarly to the PUT enzymes, is also regulated by Put3 (Fig 2), but noted that arginine also induced Gdh2 expression independent of Put3, albeit to lower levels, indicating the involvement of other transcription factors. We were surprised that glutamate and glutamine did not induce PUT enzymes (Figs 2D, S2A and S2C). This indicates that in contrast to arginine and ornithine, glutamate and glutamine are not readily converted to proline, which differs from mammalian cells where these amino acids are key precursors of cytoplasmic proline biosynthesis [2, 14]. Interestingly, PRODH expression in mammalian cells is independent of proline and is induced in response to stress through the p53 tumor suppressor protein, proliferator-activated receptor γ (PPARγ), and/or AMP-activated kinase (AMPK) [14].

The physiological source(s) of proline within the host environment needs to be determined. Although proline assimilation may be a fungal-driven process, where fungal cells actively secrete proteases and cytolytic toxins (e.g., candidalysin; [68]), it is possible that *C*. *albicans* cells acquire proline as a result of host-driven processes. Indeed, there is evidence to suggest that the proteolytic activities in the host, during pathological states such as cancer or sarcopenia contribute to proline availability [6, 7, 69], and low levels of fungal DNA and cells have been found in association with human cancers [70], suggesting that fungi are able to benefit through spatial association with cancer cells and accompanying macrophages at sites of metastatic growth, perhaps by cross-feeding fungi with host-derived nutrients. In addition, other members of the microflora can also facilitate extracellular matrix (ECM) degradation by secreting collagenase during infection, such as been shown in *H. pylori* [71]. In our *in vitro* 3D skin model (Fig 4E), the source of proline is likely collagen as dermal fibroblasts and keratinocytes secrete matrix metalloproteinases (MMP) [72]; the resulting peptide fragments can be internalized by fungal cells and degraded to liberate free proline. The catalytic release of proline from peptides intracellularly is carried out by a small subset of proteases called prolidase (or proline dipeptidase) that are capable of hydrolyzing the bond constrained within the pyrrolidine ring [73]. The uncharacterized *C*. *albicans* ORF (C1_14450C; CGD) encodes a putative prolidase [73]. Supporting the notion that host-driven processes are responsible for proline assimilation, we observed that many proline-rich proteins, including collagen, did not induce the expression of Put1 and Put2 even after 72 h of incubation. Consistently, it took 14 days for the wildtype strain to fully invade the collagen matrix (Fig 4B), suggesting that purified collagen does not induce the expression of secreted degradative proteases in *C*. *albicans*. In contrast, mucin, a highly abundant protein in the gut where systemic *Candida* infections are thought to originate [52, 74], significantly induced Put1 and Put2 expression. This suggests that *C*. *albicans* can readily assimilate proline from mucin in the gut.

Our observations that proline inhibits the growth of *put1* cells albeit to a lower extent than in *put2* cells (Fig 3A) are consistent with previous reports in yeast [75] and plants [76]. Since proline accumulates in isolated mitochondria from yeast, plants and rat liver [77–79], we presume that proline also accumulates in the mitochondria of *put1* where it inhibits an unidentified mitochondrial target. In absence of exogenous proline, the basal respiration of *put1* mutant is reduced (Fig 3E), presumably by interfering with the normal function of ETC. Consistent with this notion, in mammalian cells proline dehydrogenase (Put1) has been shown to associate with ETC Complex II at the inner mitochondrial membrane [15]. Interestingly, the growth phenotype exhibited by *put1* and *put1/put2* mutants in/on YPD also appear to overlap with some of the reported phenotypes of ETC complex mutants. In YPD, the ETC mutants exhibit an “explosive energetic phenotype”, which is characterized by elevated respiration (OCR) that appears related to increased Snf1/AMPK activity [40]. This process is inefficient and acts as a temporary remedy to generate ATP to support growth, and cells that cannot sustain this process undergo cell death, which correlates with our data showing the increase in phloxine uptake in *put* mutants (Fig 3G). Cells lacking P5C dehydrogenase (*put2*) are substantially more sensitive to proline as a consequence of P5C accumulation (Fig 3B). Proline as a substrate for ROS formation is supported by several studies using isolated mitochondria, cancer cells and *Drosophila* (reviewed in [80]). The high rate of mitochondrial electron transfer associated with proline catabolism may enhance ROS formation. However, such does not appear to be the case in *C*. *albicans*, as the addition of proline reproducibly decreased ROS in the wildtype cells (Fig 3C). This may reflect a low reactivity of Put1 towards oxygen, which contrasts with PutA from *Helicobacter pylori* and *H. hepaticus* [81]. Regardless, our data show that ROS is not the primary contributor to proline hypersensitivity of *put2*, as ROS scavengers failed to fully rescue growth (Figs 3D and S3F). Consistent with this notion, it took 6 hours after proline addition before measurable levels of ROS could be reproducibly assayed. It is possible that as *C*. *albicans* cells detect stress they employ a number of detoxification mechanisms that include superoxide dismutase (SOD) [82], and possibly, the *MPR1*-encoded protein N-acetyltransferase (Mpr1) homologue that detoxifies P5C or GSA by acetylation in yeast [83]. We traced the inhibitory effects of P5C to defective mitochondrial respiration (Fig 3E). Consistently, *put2* mutants are unaffected by proline when grown under mitochondrial repressing conditions in the presence of high glucose (2%) [10]. Our findings are well-aligned with Nishimura et al. [38], who showed that strains lacking mitochondrial DNA (*rho*^0^) exhibit diminished P5C hypersensitivity.

The attenuated virulence of *put* mutants in both insect and murine infection models (Fig 5A and 5B), suggests that proline, arginine and/or ornithine are accessible and that proline is actively used in hosts as an energy source. However, our finding that proline itself (Fig 3F) inhibits growth of *C*. *albicans* cells that lack the ability to catabolize it, complicates the interpretation that these mutants simply cannot generate energy from proline to support growth. In *Drosophila*, arginine is present at high levels in hemolymph [84]. In mice, the mean physiological levels of proline, arginine, and ornithine in blood are 269 μΜ, 137 μΜ, and 198 μΜ, respectively [85], while in human blood plasma, the levels are 245 μΜ, 44 μΜ, and 182 μΜ, respectively [86]. In the *in vitro* 3D skin model (Fig 4E), proline is made available from the degradation of collagen and keratin by dermal fibroblasts and keratinocytes that secrete matrix metalloproteinases (MMP) [72]. However, the fact that proline inhibits mitochondrial respiration in these mutants is consistent to previous reports that filamentation is dependent on mitochondrial function (Fig 4F) [87]. Phagosomes represent a microenvironment thought to be nutrient poor, but suggest the presence of significant levels of proline as judged by robust Put3-dependent expression in phagocytized *C*. *albicans* cells [10, 17]. In support of our data, proline also induces *ICL1* [33], which encodes for the glyoxylate cycle enzyme isocitrate lyase (Icl1; EC:4.1.3.1) that is well-known to be robustly induced in phagocytized *C*. *albicans* and non-*albicans Candida* species [88, 89]. Consequently, phagocytized *put* mutants are likely to experience proline-dependent inhibition in addition to the inability to catabolize proline. It is noteworthy that in contrast to Put1, Put2 is expressed at relatively high basal levels in the absence of proline (Fig 2D), which is likely beneficial to cells in that they are prepared to oxidize the toxic intermediate P5C (GSA) when proline for as low as 78 μM (S2D Fig) becomes available. Some disparities observed in the *put* mutants in various models can be due to variations in proline availability and tissue-specific responses that are beyond the scope of this work.

IVM enabled unprecedented and unanticipated discoveries of the host-pathogen interactions during early stages of infection of kidneys in a living host. Previous work showed that compared to other organs, *C*. *albicans* filament faster in the kidney suggesting the presence of kidney-specific cues. Interestingly, the kidney is a hub for arginine/proline biosynthesis, which likely increases local concentrations of arginine/proline that contribute to rapid filamentation in *C*. *albicans* [60–62]. Two-photon microscopy has previously been used to image fungal infections in translucent mouse pinna (ears) [90], however, to our knowledge, our studies represent the first in which *C*. *albicans* infections have been imaged under physiological *in situ* conditions in a complex intravital organ (Fig 6). The use of *C*. *albicans* strains constitutively expressing yEmRFP and stained with FITC enabled us to distinguish mother from growing daughter cells. IVM provided the means to critically examine several assumed, but untested, parameters regarding kidney infections. We found filamentation events in the renal cortex, although these events seem to be rare in the early phase of infection. Similar to a bacterial model of infection, where only a few *Escherichia coli* cells were found to be necessary to establish a kidney infection [64], we found that single fungal cells were able to grow and filament (Fig 6E), suggesting that multiple fungal cells are not required to initiate a colonization event. We observed that the primary locus for filamentation differed from the sites where non-filamenting *Candida* cells accumulated. Interestingly, filamenting cells localized to the intertubular space, most likely attached to the tubular cells- or capillary wall (Fig 6E). Another parameter of interest is that we observed *C*. *albicans* cells transmigrating from the capillary through different endothelial barriers (capillary and tubular) into the lumen of the tubules (Fig 6D and 6F), thus enabling us to literally observe the establishment of candiduria stemming from fungal cells growing in the kidney (pyelonephritis) as a consequence of a systemic infection. Candiduria in ICU patients is often considered an indicator of invasive candida infections [91, 92]. The inability of *C*. *albicans put2* mutant to grow and form hyphae under the same *in situ* conditions as the wildtype strain (Fig 6F) conforms to the notion that proline is available in significant quantity at the sites of infection. In addition to proline toxicity, tissue resident (CX_3_CR1^+^) renal macrophages accumulate in the kidney and offer protective role during the early stage of systemic infection [59]. This, together with our previous finding that *put* mutants also exhibit reduced survival following co-culture with macrophages [10] aligns well with reduced filamentation and lower fungal burden in the kidney of *put2*-infected mice (Figs 5D and 6F). Our future focus will be to develop better reporter strains expressing more robust fluorophores to examine the metabolic changes in *C*. *albicans* in real time by IVM. The knowledge gained from the successful application of intravital microscopy opens up the new possibilities to address fundamental questions regarding virulent fungal growth with broad biological implications.

In summary, the data presented in this work indicate that proline catabolism provides an important source of energy that facilitates *C*. *albicans* virulence and likely that of other *Candida* spp. Importantly, *C*. *albicans* cells must synchronize the activities of the catabolic enzymes, since proline is toxic, as is the intermediate P5C, making this pathway appropriate to target for the design of antifungal drugs. Small molecules that block Put1 and/or Put2 activity would be expected to inhibit fungal growth. Finally, there is growing evidence that proline serves as an energy source during diverse disease states affecting the host, including cancer and stress, which are risk factors for *Candida* infections. Given that *Candida* infections are common among immune compromised individuals, proline derived from host-driven degradative processes may be key to understanding fungal virulence. Further work is needed to dissect the complex host-pathogen interactions that impinge on proline catabolism.

## Materials and methods

### Ethics statement

All procedures using animals performed at the Experimental Core Facility, Stockholm University, Stockholm, Sweden were approved by the Stockholm Ethics committee (License nr. 9700–2018). Mice were housed in individually ventilated GM500 cages (Tecniplast) under constant humidity (50–60%) and temperature (21 ± 2°C) and with a constant (year-around) 12-h light/dark cycle. The mice were provided aspen bedding (Tapvei, article no 2212), diet deficient in phytoestrogens (Altromin 1324 variant P, article no 30047), water *ad libitum* and the following enrichment: paper play tunnel (Scanbur, article no 20-CS3B02), aspen gnawing stick (Tapvei, article no 44219999S-brick) and paper nesting material (Scanbur, article no 20-CS1A09). Mice were maintained under Specific Pathogen Free (SPF) conditions according to the FELASA guidelines (2014). For animal experiments performed in Shanghai, China, all procedures were performed in compliance with the protocol approved by the Institutional Animal Care and Use Committee (IACUC) at the Shanghai Institute of Immunity and Infection, Chinese Academy of Sciences, China (License nr. A2021003). The animal care and husbandry conditions are comparable to those at Stockholm University, Stockholm, Sweden. Mice strains and suppliers are listed in S1 Table.

For experiments involving human peripheral blood, all investigations were conducted according to the principles expressed in the Declaration of Helsinki. The Karolinska University Laboratory provided samples of peripheral blood collected from healthy volunteer blood donors. The established donation protocol for collection of human blood for purposes other than medical treatment was followed. This protocol does not require specific ethical approval; the small volumes of blood were provided with explicit consent for use in this study, the samples were labeled with donation number for traceability according to legal requirements, but no names or other personal data was provided. Upon receipt in our laboratory, the samples were de-identified and could no longer be traced to an individual. Neutrophils were isolated from the blood of healthy volunteers in compliance with the local ethical committee (Regionala etikprövningsnämnden i Umeå) as approved in permit Dnr 09–210 M with fully informed written consent of donors.

### Organisms, media, and culture

Most of the yeast strains (*Candida* spp. and *S*. *cerevisiae*) and plasmids (*E*. *coli*) reported in this work originated or were derived from the Ljungdahl (POL) strain collection (S1 Table. Key reagents and resources). *C*. *albicans* clinical isolates and non-*albicans Candida* species were obtained from several laboratories: Ute Römling (UT), Valerie Diane Valeriano (VDV), Oliver Bader (OB), Matthew Anderson (MA), Steffen Rupp (SR), Constantin F. Urban (CU) and Karl Kuchler (KK). CRISPR/Cas9 plasmids pV1093 and pV1524 were donated by Valmik Vyas (VV). All yeast and *E*. *coli* strains were stored at -80°C as 15% glycerol stock and recovered as needed on permissive media. Yeast strains were maintained on solid YPD [1% yeast extract, 2% peptone, 2% glucose (dextrose), and 2% Bacto agar] as a streak from single colonies after recovery from -80°C glycerol stocks. For yeast selection, YPD was supplemented with nourseothricin (Nou) at the indicated concentrations (200, 100 and 25 μg/ml) from a filtered stock solution (200 mg/ml) prepared in H_2_O. Where indicated, 2% glucose in YPD is replaced with 1% glycerol (YPG), 1% lactate (YPL), 0.2% glucose (YPD_0.2%_), 1% acetate (YPAc), or 2% maltose (YPM). In some specific growth assays, synthetic minimal media were used with the indicated carbon and nitrogen source: SD [0.17% YNB without amino acids and ammonium sulfate (YNB), 38 mM ammonium sulfate (Am, 5 g/L), and 2% glucose], SD* (same as SD but the Am was reduced to 5 mM), SPD (0.17% YNB, 10 mM proline, 2% glucose), SPG (0.17% YNB, 10 mM proline, 1% glycerol), SP (0.17% YNB, 10 mM proline), SGL (0.17% YNB, 10 mM proline, 1% glycerol, 1% lactate). All of the media were prepared from sterile stock solutions prepared in ddH_2_O as follows: YP (2X), YNB (1.7%), agar (4%), glucose (40%), glycerol (20%), lactate (20%, pH = 6), sodium acetate (18%), proline (200 mM), arginine (200 mM), ornithine (200 mM), glutamate (200 mM, pH = 6), glutamine (200 mM), and ammonium sulfate (1 M). The pH of lactate and glutamate solutions was titrated using 6M NaOH. All stocks were autoclaved separately except for the amino acids that were filter-sterilized (0.2 μm). Other media modifications or culture conditions are indicated in figure legends or text.

### Genetic manipulation and gene inactivation

All CRISPR/Cas9 plasmids, repair templates (RT) bearing in-frame stop codons and restriction sites, and verification primers used to generate and verify additional *put* strains were described previously [10] and can also be found in **S1 Table. Key reagents and resources** and **S2 Table. Oligonucleotides**. For plasmids, *E*. *coli* from glycerol stocks were recovered directly in liquid Luria-Bertani (LB) broth with 50 μg/ml carbenicillin and/or 50 μg/ml nourseothricin (Nou) and then processed the following day for plasmid extraction. Specific sgRNAs were cloned in either pV1093 and/or pV1524 backbones [29, 30] by blunt-end ligation. Plasmids were verified by sequencing using primer p9. Verified cassettes expressing specific sgRNAs, Cas9, and selection marker, were released from plasmids by *Kpn*I/*Sac*I digestion (~5 μg plasmid/digestion). Digested cassettes were PCR-purified and co-transformed with purified RT (generated by template-less PCR) into the indicated strain using the modified hybrid lithium acetate/DTT-electroporation method described in [10]. Nourseothricin-resistant (Nou^R^) transformants were selected on YPD+200 μg/ml Nou and further clonal purification was made on YPD+100 μg/ml Nou. Purified colonies were verified by colony PCR using the appropriate primers to amplify the mutated gene and then digested with the specific restriction enzyme (*Xho*I) to identify knockouts. For pV1524-derived plasmids, cassette excision was done by growing colonies in liquid YPM and then plated on YPD+25 μg/ml Nou. Nourseothricin sensitive (Nou^S^) pop-outs were verified by streaking on YPD+100 μg/ml Nou. In most cases, single preparations of purified digested cassettes and RT suffice for 10–15 independent transformations and were kept at -20°C until used. For targeted reconstitution, *put1-/-* and *put2-/-* strains were transformed with wildtype *PUT1* and *PUT2* gene fragments and proline-utilizing (Put^+^) colonies were selected on SPD resulting in CFG379/CFG380 (*PUT1*+/-) and CFG381/ CFG382 (*PUT2*+/-), respectively. The following primer pairs were used for routine PCR verification: *put1-/-* (p1/p2), *put2-/-* (p3/p4), *put3-/-* (p5/p6), *PUT1+/-* (p7/p2), *PUT2+/-* (p8/p4).

*C*. *glabrata PUT1* (CAGL0M04499g) and *PUT2* (CAGL0M04499g) gene deletion were done in the ATCC2001 (CBS138) background strain using the modified SAT1 flipper technique [93, 94]. Briefly, a YEp352-*NAT1*-Cg*PUT1*urdr and YEp352-*NAT1*-Cg*PUT2*urdr plasmids were generated by Gibson assembly [95]. Fragments (~10 ng/kb) composed of 500 bp of up- and downstream regions of *PUT1* or *PUT2* coding sequence (CDS), a FRT-FLP-*NAT1*-FRT fragment from pSFS3b [32], and an *E*. *coli* replication origin and an ampicillin resistance marker from YEp352-*SAT1* were assembled using a 2x Gibson assembly master mix (New England Biolabs). The deletion cassettes released by digestion using FastDigest *Nco*I and *Pvu*I, PCR-purified, and transformed into *C*. *glabrata* by electroporation. Transformants were verified for correct cassette integration and deletion by colony PCR using DreamTaq Green DNA Polymerase.

### Reporter strain construction

The triple tagged *C*. *albicans* reporter strain co-expressing Gdh2-GFP, Put1-RFP, and Put2-HA was constructed via a series of homologous recombination transformations (S1D Fig) with specific cassettes described previously [17]. The GFP tag uses *URA3* as marker, while both the RFP and HA tagging cassettes that were derivatives of the SAT1-flipper cassette [93] use Nou^R^ as a recyclable resistance marker. Starting from strain CFG407, which already co-expresses Gdh2-GFP and Put1-RFP, this strain was subjected to another round of transformation with a ~4.8 kb *PUT2-HA-CaSAT1* cassette amplified from pFA6a-3xHA-SAT1-FLP using primers p10/p11 generating the Nou^R^ strain CFG430. Cassette excision was made by growing CFG430 in YPM generating the Nou^S^ pop-outs (CFG433). To increase the flexibility of this strain for western blot works, we created strain CFG438 by transforming CFG433 with pJA21 digested with *Kpn*I/*Sac*I, which releases the *P*_*ADH1*_*-RFP-CaSAT1* fragment, enabling CFG438 to constitutively express free RFP. CFG438 was popped-out to excise the cassette generating the Nou^S^ strain CFG441. For the creation of Put2-GFP strain expressing free RFP, CFG219 (*PUT2/PUT2-GFP-URA3*) strain was transformed with *Kpn*I/*Sac*I-digested pJA21 to generate CFG237 (Nou^R^) and then popped-out to obtain CFG259 (Nou^S^). Strains CFG441 and CFG259 were used as backgrounds for CRISPR/Cas9-mediated inactivation of *PUT3* using plasmid pFS084 resulting to strains CFG443 and CFG301, respectively. For the creation of Put3-HA expressing strains, SC5314 was transformed with the *PUT3-HA-CaSAT1* cassette amplified from pFA6a-3xHA-SAT1-FLP using primers p19/p20, which then generate the strains CFG187/CFG188 (independent clones from two separate transformations).

PCR conditions for cassette amplification using either Ex-TAQ polymerase (Takara) or Phusion polymerase (Thermo Fischer Scientific) in a 300 μl (50 μl x 6) reaction volume were as follow: 1) Initial melting: 98°C, 30s; 2) 3-step: Melting: 98°C, 10s; Annealing: 55°C, 30s; Extension: 72°C, 5 min; 3) 2-step: Melting: 98°C, 10s; Extension: 68°C, 5 min; 4) Final extension: 68°C, 10 min; and 5) 4°C. After amplification, samples were pooled and then an aliquot run on 1% agarose to assess successful cassette amplification. Pooled PCR samples were purified and then stored at -20°C until used. Purified cassettes (0.2–0.5 μg) were transformed into the indicated strains and then the correct clones were screened by colony PCR, complemented by immunoblotting and/or fluorescence microscopy. In most cases, a single preparation of purified tagging cassettes is good for 10–15 independent transformations and are kept at -20°C until used. The following primer pairs were routinely used for PCR verification of cassette integration: *PUT1-RFP-CaSAT1* (p14/p16), *PUT2-HA-CaSAT1* (p12/p13), *GDH2-GFP-URA3* (p17/p18), *P*_*ADH1*_*-RFP-CaSAT1* (p15/p16), *PUT3-HA-CaSAT1* (p5/p13).

### Protein expression analysis

To analyze the expression of PUT enzymes in the presence of different nitrogen sources, overnight SGL cultures of the indicated strains were diluted into 3 ml of fresh SGL medium at OD_600_ ≈ 0.3. Cultures were grown to log phase with vigorous shaking for 6 h at 30°C and then a ~0.8 ml aliquot was added into an Eppendorf tube containing the required volume of proline (or other nitrogen source) to achieve the required 10 mM inducing concentration. Induction was performed on a table-top thermo-shaker (900 rpm, 30°C) for 1 h. After induction, whole cell lysate was prepared by NaOH/trichloroacetic acid (TCA) precipitation; briefly, cells were mixed with 280 μl of ice-cold 2 M NaOH with 7% β-mercaptoethanol (βME) to the tube and then incubated for 15 min on ice. Lysed cells were mixed with an equal volume of cold 50% TCA to precipitate proteins overnight at 4°C. Protein precipitates were collected by centrifugation at 17,000*g* (4°C, 10 min), solubilized completely in the required volume of 2X SDS sample buffer containing 5% βME and 167 mM of Tris, and then boiled at 95–100°C for 5 min. Samples were either processed immediately for western blot or stored at -20°C until run. For Put3 expression analysis, strains (CFG187/CFG188) expressing Put3-HA were first grown overnight in either YPD or SGL. The following day, cultures were diluted to OD_600_ ≈ 0.3 in their respective medium where they are allowed to grow exponentially for 4 h (YPD) and 6 h (SGL) at 30°C before collecting cells for whole cell lysate preparation by NaOH/TCA method and western blot.

For induction of PUT enzymes during growth on protein as sole nitrogen source, strain CFG433 was grown in modified Hanks Balanced Salt Solution (HBSS) [NaCl (138 mM), KCl (5.33 mM), KH_2_PO_4_ (0.44 mM), Na_2_HPO_4_ (0.3 mM), MgCl_2_ (0.5 mM), MgSO_4_ (0.41 mM), CaCl_2_ (1.26 mM), NaHCO_3_ (4 mM), HEPES (25 mM, pH = 7.4), Biotin (2 μg/ml), 3.8 mM glucose, and 0.83 mM lactate] containing the following nitrogen sources: collagen (from human placenta), human serum albumin, hemoglobin (from bovine blood), mucin (from porcine stomach Type II) or 10 mM ammonium sulfate. These proteins were aseptically dissolved in HBSS at 0.5 mg/ml prepared fresh for every experiment. This HBSS was modified to reflect the level of glucose and lactate in interstitial fluid [96]. To maintain sterility, this medium was supplemented with Penicillin and Streptomycin (Penn/Strep) commonly used in cell cultures; 100–200 μl aliquots of these solutions were spotted on YPD for sterility check. We used collagen from human placenta (Type IV) since it is readily soluble in this buffer. Other types of collagen (e.g., from bovine Achilles tendon or rat tail) were inappropriate for direct comparison since they require acetic acid for dissolution. To start the experiment, a single CFG433 colony from freshly streaked YPD plates were pre-cultured in 3 ml of SGL medium for 8 h and then afterwards diluted in 50 ml of fresh SGL at OD_600_ ≈ 0.05 followed by growth for 16 h at 30°C. Cells were washed twice with ddH_2_O and then resuspended in 0.5 ml of ddH_2_O to concentrate the cells. Washed cells were added to 3 ml of medium in a 6-well microplate at OD_600_ ≈ 1. Plates were incubated static in a humidified incubator at 37°C with 5% CO_2_ for 1 or 3 days. After the indicated timepoint, cells were recovered gently using rubber scraper and transferred into a 15 ml falcon tube. The wells were washed twice with 3 ml of ice cold ddH_2_O and the washings pooled into the same 15 ml tube. The cells were washed twice and resuspended in 1 ml of cold ddH_2_O. A 0.8 ml aliquot of this is subjected to whole cell lysis by NaOH/TCA method and the rest analyzed by OD_600_. Protein pellets were solubilized in appropriate volume of 2X SDS sample buffer calculated from OD_600_, boiled, and then subjected to immunoblotting to detect the indicated proteins.

### Immunoblot

Denatured proteins were first resolved in 4–12% Bis-Tris or 3–8% Tris-Acetate pre-cast gel in 1X NuPAGE SDS running buffer (MES, MOPS, or Tris-Acetate) for ~1.5 h at 150V and then (electro)transferred onto a nitrocellulose membrane for 1 h in a 1X Tris-glycine transfer buffer with 20% ethanol (120V, ~1.5 h) in a Bio-Rad Mini Trans-Blot Cell apparatus. After protein transfer, membranes were blocked with gentle agitation in 10% skimmed milk (MSK) solution in TBST for 1 h. Target proteins in the membrane were probed either individually or simultaneously using an optimized antibody cocktail prepared in 5% MSK/TBST solution; primary antibody cocktail (overnight incubation at 4°C): α-GFP (1:3000), α-mCherry (1:6000), α-Tdh3 (1:5000), α-actin (1:5000) and secondary antibody cocktail (1 h incubation at room temperature): goat α-mouse poly-HRP (1:15000), goat α-rabbit poly-HRP (1:10000), α-HA-HRP (1:15000), α-tubulin-HRP (1:2500). After extensive washing with TBST, membranes were added with chemiluminescent substrate (SuperSignal West Dura Extended Duration Substrate) to detect the immunoreactive bands using the Azure 280 or ImageQuant LAS 500 detection system. Signal quantification on TIFF files were made using Image Lab (Bio-Rad).

### Subcellular fractionation

For subcellular fractionation analysis, *C*. *albicans* cells (CFG433) were collected from overnight YPD culture (100 ml), washed twice with ddH_2_O, and then inoculated into 100 ml of prewarmed YPG (in 500 ml flask) at OD_600_ ≈ 2. Cultures were grown under aeration (>150 rpm) for 4 h at 37°C before harvesting cells for cytosolic and mitochondrial fractionation according to the protocol by Meisinger et al. (2006)[97] with minor modifications described in our previous work [17]. Briefly, spheroplasts prepared using Zymolyase-100T were homogenized in a glass-Teflon homogenizer and then the diluted lysate (L) was subjected to two low-speed centrifugation steps to remove cell debris and unbroken cells. The supernatant obtained after the second centrifugation step (4,000*g*, 5 min, 4°C; SL 40R, Thermo Scientific) was labeled as the total cell lysate (T) and subjected further to high-speed centrifugation at 12000*g* (JA-14.50, Beckman Coulter) for 15 min at 4°C. The resulting crude mitochondrial (pellet) and cytosolic (supernatant) fractions were further processed to remove contaminating components from either the cytosol or mitochondria, respectively (See Fig 1B for scheme). One (1) ml of crude cytosolic fraction was spun down on a table top centrifuge at 17,000*g* for 10 min at 4°C and the resulting supernatant (C) was used. To further purify the crude mitochondrial fraction, the pellet was washed twice without resuspension with 20 ml of cold homogenization buffer and then the pellet resuspended gently in 1 ml of ice-cold SEM buffer; the mitochondrial suspension was first centrifuged at low speed (4000*g* for 10 min) and the resulting supernatant was centrifuged at 12000*g* for 10 min to collect the pellet highly enriched in mitochondria (M). Protein suspensions with normalized concentration (20 mg/ml) were diluted in 2X SDS sample buffer and then processed for immunoblot (see previous section) but with the following antibody cocktail: primary: α-GFP (1:3000), α-mCherry (1:6000), α-Tdh3 (1:5000), α-ATP5a (1:2000), and secondary: goat α-mouse poly-HRP (1:15000), goat α-rabbit poly-HRP (1:15000), α-HA-HRP (1:15000).

### Liquid growth assay

For growth in liquid synthetic minimal medium in tubes, the indicated strains picked from single colonies were pre-cultured overnight in SD or SGL at 30°C with aeration. The following day, cultures were refreshed in the same medium at a starting OD_600_ ≈ 0.3 and volume of 6 ml. After 3 h of growth, cultures were split equally into two tubes (3 ml/tube) and then added with either proline (10 mM) or equal volume of ddH_2_O. Spiked cultures were incubated for 20 h prior to OD_600_ measurement using portable cell density meter (WPA CO 8000, Biochrom, UK). For growth with antioxidants (N-acetyl cysteine (NAC), Mito-TEMPO, or TIRON), cells were grown for 2 h before splitting the cultures (3 ml/tube). These cultures were diluted 1:1 with 3 ml of fresh medium without or with 2X strength of the compound (dissolved or added directly in the medium followed by 0.2 μm filter-sterilization). Cultures were incubated for another 1 h before spiking with proline (10 mM) or ddH_2_O and the growth determined 20 h later. For growth in rich complex medium, cells from overnight YPD cultures were washed at least 2X with ddH_2_O and then inoculated into 4 ml of YPD, YPG, YPL, and YPAc at a starting OD_600_ ≈ 0.01 after which growth was recorded 24 h later. When flocculation is to be observed, cultures at the indicated timepoints were vortexed and allowed to stand for at least 3 min prior to photograph.

For high throughput growth assays in microplate format, cells of the indicated genotypes were pre-grown in SGL and then diluted directly into the same medium at OD_600_ ≈ 0.05. Using a multi-channel pipette, 100 μl aliquots of this adjusted cell suspension were aseptically transferred into 96 well plate followed by the addition of proline at the indicated final concentrations (0.0195 mM– 10 mM) prepared from 200 mM stock. Growth (as absorbance at 600 nm) was monitored every 5 min in a TECAN microplate reader set at 30°C with continuous linear shaking (1440 rpm). The same procedure was applied for growth in other media (e.g., YPD) but without proline addition.

For minimum inhibitory concentration (MIC) determination, strains were grown in SGL containing different proline concentrations as described in the preceding paragraph, but plates were incubated in a standard orbital shaking incubator (Infors HT Multitron Incubator Shaker) set at 30°C and 130 rpm for 24 h before measuring growth in a TECAN microplate reader as an endpoint read. Due to the tendency of *put* mutants to aggregate, wells were mixed using a multichannel pipette prior to reading. The MIC is defined as the lowest proline concentration that gives rise to ≥50% growth inhibition of the proline-free control.

### Assessment of fungal growth on solid media

To assay growth on solid medium by drop plate, cells were collected from overnight YPD or SD cultures, washed twice in ddH_2_O, and then adjusted to OD_600_ ≈ 1. Five microliters of 10-fold serially diluted cell suspension in ddH_2_O prepared in sterile 96-well microplate were spotted onto the solid medium. Spots were allowed to dry before incubating the plates inverted at 30°C for 48 h (2 days) and photographed. Filamentation on solid Spider medium was carried out as previously described [17]. Briefly, cells from overnight YPD cultures were collected, washed three times in PBS, and then a 5 μl aliquot of OD_600_ ≈ 1 cells was spotted on the surface of Spider medium (1% nutrient broth, 1% mannitol, 0.2% K_2_HPO_4_, 1.35% agar, pH = 7.2) first reported by Liu *et al*.[50]. Macrocolonies were examined and photographed after 6 days at 37°C. Other specific growth conditions are indicated elsewhere in the text.

### Fungal cell vitality assay

To assay cell vitality on macrocolonies, Phloxine B (PXB) was used [43, 46, 98]; PXB accumulates in dead or metabolically inactive cells. Briefly, cells from overnight YPD cultures were harvested, washed twice in ddH_2_O, adjusted to OD_600_ ≈ 1, and then a 5 μl aliquot was spotted on the indicated solid medium with 10 μg/ml of Phloxine B followed by incubation at 30- or 37°C for 3 days. PXB plates were prepared by aseptically adding the appropriate volume of PXB (10 mg/ml in ddH_2_O stock) to molten agar (~75–85°C) and held in this temperature for several minutes (>10 min) to ensure sterility. For cells grown in liquid cultures, propidium iodide (PI) was used as a viability stain; PI is a membrane impermeant dye that is excluded from viable cells and that intercalates to DNA of dead necrotic cells to exhibit bright red fluorescence. Briefly, cells were grown as indicated in the specific text and then a 1 ml aliquot of the culture was mixed with 1 μl of PI (1 mg/ml in H_2_O) to a final concentration of 1 μg/ml. Within 3 min after adding the dye, the cells were examined under fluorescence microscope (Cell Observer or Axio Observer 7) in the red and DIC channels. Images were captured from at least 3 different frames.

### P5C quantification

From a 30 ml log phase SGL culture (OD_600_ ≈ 1.5–2.5) of each indicated strain, 6 ml aliquot were transferred to separate tubes containing proline (10 mM final concentration) or ddH_2_O. Cultures were incubated for 2 h at 30°C with aeration and then a 100 μl aliquot was transferred to each well of a round bottom 96-well microplate placed on ice. Exactly 100 μl of 10% TCA was added to each well and were allowed to incubate for 15 min protected from light. The direct extraction of P5C in TCA is crucial as P5C is stable in acidic condition [38]. Using a multichannel pipette, a 50 μl volume of 2-aminobenzaldehyde (2-ABZ) solution in 99.5% ethanol was added to each well and then incubated on ice for another 5 min. Plates were centrifuged at room temperature for 5 min at 4,000 rpm (SL 40R, Thermo Scientific). Supernatants (100 μl) were transferred to a fresh 96-well microplate (clear, flat bottom) and then immediately analyzed for absorbance at 444 nm using the Enspire microplate reader (Bio-Rad). All readings were normalized to cell density (OD_600_) and were presented as fold change relative to wildtype strain grown without proline.

### ROS assay

For detection of total ROS in strains grown in the presence or absence of exogenous proline, the chemiluminescent luminol-horse radish peroxidase (HRP) system was used. Luminol (Cat. # 123072, Sigma-Aldrich) was dissolved in DMSO (200 mM stock) while HRP (Type 1; Cat. # P8125, Sigma-Aldrich) was dissolved in PBS (300 units/ml stock). From a 30 ml log phase SGL culture (OD_600_ ≈ 1.5–2.5), a 6 ml aliquot was spiked with either 10 mM proline (final concentration) or equal volume of ddH_2_O. Cultures were grown for 6 h at 30°C and then a 100 μl aliquot was transferred to each well of a flat-bottom Nunc 96-well white microplate. For control samples, H_2_O_2_ (50 mM; positive control), N-acetyl cysteine (NAC, 10 mM; general ROS scavenger), MitoTempo (MT, 100 μM; mitochondrial-specific superoxide scavenger) was added to the indicated tubes 30 min (i.e., after 5.5 h) before transferring to microplate. Using a multi-channel pipette, a 100 μl of 2X strength luminol-HRP mixture in 100 mM HEPES (pH = 7.4) was added to each well and then mixed; the final concentration of these components per well are as follows: luminol (200 μM), HRP (0.12 units), and HEPES (50 mM). Plates were kept at room temperature for 10 min protected from light before reading the luminescence in Enspire microplate reader (Biorad). Signals were acquired in plate mode set at 30°C, 1 sec integration time and repeated every 3 min for at least 45 min. All readings were normalized to cell density (OD_600_) and results were presented as both signal accumulation over time and area under the curve (AUC).

### Oxygen consumption

Cells of the indicated strains were first grown aerobically to log phase (OD_600_ ≈ 1.5–2.5) in a baffled flask containing 20 ml of SGL. From this culture, 4 ml were added to tubes containing proline (10 mM final concentration) or ddH_2_O. Cultures were incubated for 4 h in a shaking incubator (30°C) to allow for enzyme expression and then analyzed immediately for oxygen consumption. Oxygen consumptions were measured at 30°C using a high-resolution oxygraphic system (Oxytherm+R system, Hansatech Instruments Ltd) calibrated using air-saturated ddH_2_O and sodium sulfite (Na_2_SO3) for maximum and zero oxygen levels, respectively. Data acquisition was performed using OxyTrace+ Windows software every 1 sec. Briefly, 1.7 ml of pre-warmed (30°C) cellular respiration buffer (1xPBS supplemented with 407 μΜ MgSO_4_, 493 μΜ MgCl_2_, 1.26 mM CaCl_2_, 0.2% BSA) was added into the oxygen electrode chamber and then the signal allowed to stabilize for ~3 min with stirrer speed set at 75 rpm. Using a 1 ml syringe with long needle (Sterican; 23G x 2 3/8", Ø0.60 x 60 mm), a 0.3 ml aliquot of the culture is added into the oxygen electrode chamber via the injection port and then the signal acquired for ~5 min. While reading, an aliquot of the culture is also diluted for viable cell count. Rates (in nmol ml^-1^ min^-1^) were obtained from the slopes (3 min) derived using the line of best fit function and then normalized to cell density.

### ATP quantification

For measurement of intracellular ATP content, a bioluminescence-based detection kit (#A22066; Molecular Probes, Invitrogen) was used. Colonies of the indicated strains from freshly streaked glycerol stocks were resuspended in 300 μl of ddH_2_O and diluted in 6 ml of fresh YPD at OD_600_ ≈ 0.01 followed by growth for 16 h at 30°C. Cells were then harvested, washed twice with sterile ice-cold Tris Buffered Saline (TBS; 50 mM Tris-HCl, pH 7.5, 150 mM NaCl), and then resuspended in TBS. Cell density was adjusted to OD_600_ ≈ 20 in a 1 ml total volume. Cells were then harvested at 10,000*g* for 3 min (4°C) before re-suspending the entire pellet in a modified TCA buffer containing 100 mM Tris-HCl (pH = 8.0), 2% trichloroacetic acid (TCA), 25 mM ammonium acetate, and 4 mM EDTA. TCA concentration was reduced to 2% from the previously used concentration of 10% [10] to achieve a final concentration of < 0.01% TCA following dilution in the luciferase reaction. Cell suspension was transferred to pre-chilled tubes containing glass beads and then subjected to bead beating (Bio-Spec; 5 × 1 min, 4 M/s with 2 min on ice between pulses). Cell lysates were collected and a portion of the supernatant was first diluted 20X using Tricine buffer (pH = 7.5) and then analyzed for ATP following the manufacturer’s instruction. Luminescence was analyzed using microplate reader (Berthold) with 1 sec integration time. A portion (400 μl) of the same lysate was used to determine total protein concentration. Briefly, the lysate was mixed with 77 μl of 50% TCA to bring back the TCA concentration to 10%. After 15 min incubation on ice, precipitated proteins were collected by centrifugation at max speed (17000*g*) for 10 min (4°C). Supernatant was discarded and then the pellet mixed with 30 μl of 0.2 M NaOH. The neutralized pellet was dissolved further in 370 μl of RIPA buffer (pH = 8.0) to resolubilize the protein. After lysate clarification at 12, 000 rpm for 10 min (4°C) the supernatant was diluted and then analyzed for protein using the bicinchoninic acid (BCA; Sigma) assay. Results presented are average of ATP normalized to total protein concentration analyzed from 4–5 biological replicates performed in duplicate.

### Collagen matrix invasion assay

Prior to start of the assay, a 400-μl aliquot of ice-cold PureCol EZ gel (Cat. # 5074; Sigma-Aldrich) was added onto each transwell (PET membrane, pore size = 8 μm; VWR International, PA, USA) in a 24-well microplate and then allowed to solidify for 1 h at 37°C in a humidified CO_2_ incubator. For fungal cell preparation, cells from log phase YPD culture were harvested, washed twice in PBS, and then diluted to OD_600_ ≈ 0.1. A 1 μl aliquot of the adjusted cell suspension was carefully added directly on top of the collagen. Each transwell was aseptically transferred to a separate well containing 1 ml of complete D10 medium acting as the receiving medium. For competition assay, cell suspension of both wildtype and the *put* mutant were mixed 1:1 and then a 1 μl aliquot was added directly on top of the matrix. Fungal cells were allowed to invade the matrix for 14 days in a humidified chamber set at 37°C with 5% CO_2_. In each assay, the *cph1*Δ/Δ *efg1*Δ/Δ strain (CASJ041)[31] was used as non-filamenting control. For assessment of individual strains, the receiving medium was serially diluted and CFU determined. For competition assay, at least 50 colonies on plates from appropriate dilutions were scored for growth on SPD plate to determine the wildtype or *put* mutants (i.e., growth = wildtype; no growth = *put* mutant) and were compared against the input ratio. For wildtype or *cph1*Δ/Δ *efg1*Δ/Δ scoring, colonies were spotted on Spider medium and grown for up to 6 days at 37°C where the wildtype strain undergoes heavy wrinkling due to filamentous growth. Results presented are from 5–7 biological replicates.

### Reconstituted human epithelial (skin) (RHE) model

For the generation and infection of human in vitro skin model derived from S1F (immortalized human dermal fibroblasts) and Ker-CT (immortalized human keratinocytes) cell lines, the procedure was performed essentially as described [51] with minor modifications with respect to media exchange to keep the glucose levels down enabling relief from glucose repression in *C*. *albi*cans. In this specific work, media replacement, which is usually performed directly prior to infection, was halted 3 days prior to infection.

### Confocal (Airyscan) Microscopy

For microscopic analysis, strain (CFG407) expressing both Gdh2-GFP and Put1-RFP was grown as in for subcellular fractionation i.e., 4 h growth in YPG at 37°C. Cells were collected, washed, and then resuspended in PBS before viewing the cells using laser scanning confocal microscope (LSM800, 63x oil; Airyscan mode) in the green and red channels excited with 488 nm and 561 nm lasers, respectively, with Differential Interference Contrast (DIC) taken separately. Z-stack confocal images were obtained and the raw Airyscan images were processed using the Zen Blue software’s Airyscan processing function.

### *Drosophila* virulence assay

The *Bom*^*Δ55C*^ *Drosophila* stock was maintained on standard cornmeal agar medium at 25°C. This fly strain was originally obtained from Bloomington stock center and maintained in Prof. Ylva Engström laboratory, Stockholm University. The *Bom*^*Δ55C*^ mutant flies were collected and transferred to 29°C for three days prior to injection of fungal cell suspensions. Infection of *D*. *melanogaster Bom*^*Δ55C*^ flies was performed as described [99]. Flies were injected with approximately 500 cells/fly and then maintained in separate vials where fly survival was monitored for at least six days at 29°C.

### Human whole blood infection

Freshly extracted whole human blood samples were obtained from Blodcentralen (Odenplan, Stockholm, Sweden). The sample tubes contained EDTA as anticoagulant. For infection, the protocol by Kammer et al. [55] was adopted with minor modifications. Briefly, fungal cells were first grown overnight in YPD at 30°C and then subcultured in fresh medium the following day to obtain exponentially growing cells. Cells were collected, washed twice with PBS, and suspended to 1 x 10^7^ cells/ml. A 10 μl aliquot, ≈ 1 x 10^5^ cells, were added to 400 μl of whole blood, mixed gently, and incubated for 1 h in a shaking heat block (Eppendorf) set at 37°C and 400 rpm. After 1 h, blood cultures were vortexed and aliquots were serially diluted in ddH_2_O (to lyse human cells) and plated on prewarmed YPD agar. Colonies (CFU) growing on the agar were counted after 2 days at 30°C. The percent survival was calculated by comparing the CFU after 1h to CFU of the inoculum.

### Neutrophil killing assay

To assess the sensitivity of the proline catabolic mutants towards neutrophil attack, fungal cells were co-cultured with human neutrophils freshly isolated from peripheral blood of healthy human donors using Histopaque 1119 (Sigma-Aldrich) described in [57]. Neutrophils in RPMI1640 medium without phenol red were seeded at 100,000 cells/well (24-well plate) and then incubated in a humidified chamber (37°C, 5% CO_2_) for at least 30 min to allow the neutrophils to adhere to the culture dish. For the preparation of fungal inocula, *C*. *albicans* cells were first grown overnight in YPD, subcultured to log phase, washed, and then adjusted to ~10^6^ CFU/ml in RPMI. *C*. *albicans* cells were mixed with neutrophils at MOI of 5:1 (*Candida*:neutrophils) and then co-cultured for 2 h (t = 2) in the humidified chamber after which the neutrophils were lysed to release fungal cells. Lysates were diluted and plated on YPD agar to determine viable fungal count. For t = 0, neutrophils were immediately lysed after adding the fungal cells. The % killing after t = 2 was calculated based on the CFU obtained initially at t = 0.

### Mouse infection model

For the murine model of hematogenous disseminated candidiasis, the wildtype *C*. *albicans* SC5314 and the three *put* mutants were individually inoculated to YPD broth and grown overnight at 30°C. Cells were harvested and washed three times with phosphate-buffered saline (PBS), and counted using hemocytometer. A group of 6–8-week female C57BL/6 mice (n = 10) were intravenously inoculated with each of the indicated strains, using an inoculum of 2x10^5^ CFU per mice. The mice were monitored once daily for weight loss, disease severity and survival. The survival curves were statistically analyzed by the Kaplan-Meier method (a log-rank test, GraphPad Prism). The fungal burden was assessed by counting CFU. Mice were euthanized on the 5^th^ day after infection. The organ tissues, including kidneys, spleen, brain and liver, were collected, homogenized and appropriately diluted for plating on Sabouraud Dextrose Agar (SDA) medium. After incubation at 30°C for 48 h, colony counts were performed and data were statistically analyzed. For histological analyses, the mice were euthanized on the 5^th^ day after infection and the kidneys were fixed in 4% paraformaldehyde at room temperature. The sections were stained with periodic acid-schiff (PAS) and photographed.

For systemic infection in BALB/cAnNCrl mice performed in Stockholm, a group of at least ten 6- to 8-week-old mice per strain were infected via the lateral tail vain with 5x10^5^ CFU of *C*. *albicans* wildtype (SC5314) or *put2* (CFG318) mutants. Survival was scored daily; moribund mice were sacrificed and death was recorded the following day. For assessment of fungal burden in the kidney, mice from each group were sacrificed (2 days) and kidneys removed and homogenized in PBS by bead-beating using glass plating beads (4.5 mm). CFU counts were determined from diluted tissue homogenates plated on YPD agar with 30 μg/ml chloramphenicol. Survival curves between wildtype and *put2*-infected mice were analyzed by the Kaplan-Meier method whereas the difference in fungal burden was analyzed by student *t*-test, both performed in GraphPad Prism.

### Intravital and *ex vivo* two (2)-photon microscopy

BALB/cAnNCrl and BALB/cByJ SOPF mice (8–10 weeks old) were injected into the tail vein with 5x10^5^ CFU of *C*. *albicans* wildtype or *put2* (both constitutively expressing a modified version of mCherry (yEmRFP) under the control of the *ADH1* promoter) in sterile PBS. Prior to injection, cells were pre-stained with FITC (1 mg/ml in carbonate buffer, pH = 9.6) for 30 min at 30°C protected from light and then washed three times with PBS before adjusting cells to 5 x 10^5^ cells per 100 μl. Where indicated, samples of the FITC-stained cells were inoculated into either complete DMEM medium (DMEM high glucose supplemented with 10% FBS and penicillin/streptomycin) or DMEM SILAC medium [DMEM SILAC Flex supplemented with 4.5 g/l glucose, penicillin/streptomycin, 25 mM HEPES (pH = 7.4), auxotrophic supplements (10 μM arginine, 80 μg/ml uridine), and used without FBS] to induce filamentation in a humidified chamber set at 37°C, 5% CO_2_ for 2–3 h before taking photograph in a fluorescent microscope with filters appropriate for FITC and mCherry. For *ex vivo* imaging, the animals were sacrificed 24 h post injection, the kidneys were removed, submerged in physiological saline and imaged immediately for max. 90 min. For intravital imaging, mice were immediately or 22–24 h post injection anesthetized with isoflurane, abdominal imaging windows were implanted above the left kidney, transferred immediately to the microscope and imaged for a maximum of 4h under isoflurane anesthesia; during imaging, mice were lying on a heating blanket and their body temperature was constantly monitored, and 100μl/h physiological saline was injected subcutaneously to prevent dehydration. After the last image acquisition, the animals were euthanized by cervical dislocation. Intravital and *ex vivo* microscopy was performed using a Leica SP8 DIVE inverted confocal laser scanning microscope (Leica Microsystems, Wetzlar, Germany), equipped with a pulsed multiphoton laser INSIGHT DUAL X3 with 80MHz repetition rate (Spectra Physics, MKS Instruments, Inc., Andover, Massachusetts, USA) and 4Tune spectral non-descanned detectors. Excitation occurred with a laser power of 5–8% and at 1075nm (mCherry), at 930nm (FITC) and at 780nm (autofluorescence), fluorescence was collected at 580-680nm (mCherry), at 492-550nm (FITC) and at 380-460nm (autofluorescence). Images were acquired with HC PL APO 20x/0.75 CS2 objective lenses (Leica Microsystems, Wetzlar, Germany). The whole surface of kidneys, attached to the glass of the imaging windows for intra vital or the cover slip for ex-vivo imaging (both equal in size), were scanned using the spiral scan mode at low pixel resolution and high speed in the navigator function of the LAS-X imaging software. All spots indicating the presence of Candida cells in either the FITC or yEmRFP channel were checked in detail at higher resolution. All spots with FITC and yEmRFP positive spheres (3–5μm size) were imaged in xyz at high resolution. Image acquisition, deconvolution and data analysis was performed using the software platform LAS X and integrated Hyvolution function (Leica microsystems, Wetzlar, Germany), enabling deconvolution of with Hygens Essential (Scientific Volume Imaging, Hilversum, Netherlands).

### Statistical analysis

Data obtained in this work were analyzed using GraphPad Prism version 9. Specific statistical treatment applied is described in the figure description. In addition, the type of error bars (SEM, SD, or CI (95%)) is dependent on the type of analysis performed. Statistical analysis was performed using the means of at least 3 independent experiments, and statistical significance was determined using unpaired *t*-test, regular one-way analysis of variance (ANOVA) or Kruskal-Wallis (non-parametric) test followed by the indicated post hoc multiple comparison test, two-way ANOVA with Sidak’s post hoc test. For survival analysis, the Log-rank (Mantel-Cox) test was used; multiple comparison of survival curves was analyzed by comparing the *p* values of compared curves to the Bonferroni-corrected α value. The following set of notations were used to describe statistical significance: *p<0.05, **p<0.01, ***p<0.001, ****p<0.0001, ns = not significant.

## Supporting information

S1 Fig Strain construction and targeted reconstitution. (**A**) Targeted reconstitution: *put1-/-* (CFG154) and *put2-/-* (CFG318) strains were transformed with wildtype *PUT1* and *PUT2* fragments, respectively, and proline-utilizing (Put^+^) colonies were selected on SPD. As control, the *PUT2* and *PUT1* fragments were introduced into the *put1-/-* and *put2-/-* strains, respectively; no transformants were obtained. (**B**) Verification of the reconstructed *PUT1* and *PUT2* alleles. The colonies with arrows (**A, B**) were purified and their genomes analyzed by PCR-RD. The heterozygosity at the indicated gene locus was confirmed, i.e., *PUT1+/- (PUT1+/put1-)* and *PUT2*+/- (*PUT2+/put2-*). Primers (shown in blue) facilitate the amplification of both the wildtype Alleles 1 and *Xho*I containing CRISPR/Cas9 inactivated Alleles 2. The amplified fragments were digested with *Xho*I and fragment lengths were analyzed by electrophoresis (1% agarose gel). The fragments with the inactivated alleles are cleaved by *Xho*I resulting in two bands (indicated by the brackets), whereas the reconstructed wildtype fragment is refractory to *Xho*I digestion and runs as a single band (arrow). (**C**) *C*. *albicans cph1*Δ/Δ *efg1*Δ/Δ (WT, CASJ041), *cph1*Δ/Δ *efg1*Δ/Δ *put1-/-* (CFG344), *cph1*Δ/Δ *efg1*Δ/Δ *put2-/-* (CFG345) and *C*. *glabrata* (WT, CBS138), Cg*put1*Δ (GFS003), Cg*put2*Δ (GFS005) strains were spotted on non-selective (SD) and selective (SPD) media as indicated. The inactivation of *PUT* genes resulted in the lack of growth on selective media, indicating that inability of the mutants to use catabolize proline. (**D**) Schematic diagram of steps required to construct the triply-tagged reporter strain. The starting parental strain was CAI4 (*ura3/ura3*); the complete genotypes of the strains are listed in Methods. PCR-verification of reporter constructs used two pairs of primer pairs described in Methods. Sizes of fragments amplified with primer pairs flanking the tag inserts: *GDH2-GFP* (1.6 kb), *PUT1-RFP* (2.1 kb), *PUT2-HA* (2.3 kb), *P*_*ADH1*_*-RFP* (1.2 kb).
(TIF)

S2 Fig Put3-dependent and -independent induction of PUT enzymes. (**A**) Exponentially growing cultures of triple tagged reporter strains CFG441 (*PUT3*) and CFG443 (*put3*) in SGL were induced with 10 mM of the indicated nitrogen source for 1 h. Cell extracts were prepared and the expression of the Put1, Put2 and tubulin was analyzed by immunoblot using an optimized antibody cocktail (see Methods). Note that Put1-RFP is not detected in cultures containing either glutamate or glutamine. (**B**) Specificity of Put3 to proline. Strains, growth and analysis as in (**A**) of cultures induced with the addition of 10 mM of the indicated compounds: Ammonium sulfate (Am); L-ornithine; L-arginine; L-proline; S-(-)-proline; T-4-hydroxy-L-proline; and Azetidine carboxylate (AzC). Gdh2-GFP signals (*) were separately enhanced (lower panel) via the high slider in Image Lab (BioRad). (**C**) Glutamate is not readily metabolized to proline. Strain CFG469 was grown and induced as in (**A**) with 10 mM ammonium sulfate (Am), glutamate or proline as indicated. Note that Put1-RFP was not detected (ND) in Am or glutamate induced cultures. The immunoblots are representative of at least three independent experiments. (**D**) PUT enzyme expression is sensitive to low inducing levels of proline. Cells were grown as in (**B**) but induced with 78 μM proline for 1 and 2 h. The blot shown is representative of at least 3 independent experiments.
(TIF)

S3 Fig Proline catabolic mutants are sensitive to exogenous proline. (**A**) Serially diluted *C*. *albicans* cells of the indicated genotypes (same as in Fig 1C**)** were spotted onto buffered synthetic minimal medium (pH = 6) containing 5 mM ammonium sulfate (Am) as nitrogen source and either 1% glycerol or 2% glucose as carbon source. Excess proline (10 mM) was added as indicated. Plates were photographed after 4 days of growth at 30°C. (**B**) Strain CFG433, grown to log phase in SGL or SD, was induced with 10 mM proline for 1 h, and cell extracts were analyzed by immunoblot. The signals from Put1-RFP and Put2-HA were normalized to α-tubulin. Data presented are from 4 biological replicates (Ave. with 95% CI; ****p<0.0001 by student *t*-test). (**C**) Cells from 72 h-old SD cultures of the indicated strains (same as in Fig 3A**)** were plated for single colonies on YPD and grown for 2 days. The colonies from the *put2* mutant are heterogenous in size, both large and small colonies (black arrows) are evident. Images were representative of at least 3 biological replicates. Scale 1 = cm. (**D**) Five μl of cell suspensions of the indicated strains (same as in Fig 3A**)** were spotted on buffered SEM medium (pH = 6) containing 10 mM glutamate with and without 10 mM proline as indicated. The resulting macrocolonies were photographed after 72 h at 30°C. (**E**) N-acetylcysteine (NAC) reduced ROS in *put2* cells treated with proline. Experiments were performed as in Fig 3C but 30 min prior to reading the luminol-HRP signal, 10 mM NAC was added to cultures to sequester ROS. Data are presented as mean with 95% CI (n = 3). (**F**) ROS (superoxide) scavengers failed to rescue the growth of *put2* in the presence of exogenous proline. CFG318 was grown as in Fig 3D with and without the indicated concentrations of Mito-TEMPO or TIRON. The results are the average of at 4 biological replicates (with 95% CI). (**G**) Growth of WT (SC5314) and *put2* (CFG318) strains in SGL in a 96-well microplate in the presence of the indicated concentrations of proline. Data is presented as mean ± SD (n = 4).
(TIF)

S4 Fig Cells lacking the capacity to catabolize proline exhibit reduced vitality. (**A**) Growth of *put* mutants after 24 h in the indicated media containing different carbon sources [2% glucose (YPD), 1% glycerol (YPG), 1% lactate (YPL)]. Note that *put2* cells grown in YPG have noticeably higher number of cells forming trimera a phenotype indicative of stress (black arrows, inset). Each bar represents the mean ±SD (n = 4). Results per carbon source were analyzed by one-way ANOVA with Dunnett’s posthoc test relative to wildtype (****p <0.0001, ***p <0.001, **p <0.01, *p <0.05). (**B**) Ten-fold serial dilutions of cell suspensions (OD_600_ ≈ 1) were spotted on YPD, YPG or YPL grown for 2 days at 30°C. (**C**) Strains were streaked on YPD and grown for 10 days at 30°C. Note the increase in yellow hue in *put2* and *gdh2 put2* strains. (**D**) Five microliters of *cph1*Δ/Δ *efg1*Δ/Δ (CASJ041) or its derivatives (*put1*, CFG344; *put2*, CFG345; *gdh2*, CFG352) were spotted on the indicated plates containing Phloxine B and incubated for 3 days at 30- or 37-°C. (**E**) Flocculation in YPD cultures was assessed after 16 and 48 h of growth at 30°C. Cultures were vigorously vortexed and let stand immobile for 3 min and photographed. **(F)** Microscopic inspection of 16 h-old cultures as in (**E**) showing onset of flocculation in *put* mutants (Scale bar = 10 μm). **(G)** Growth curves of WT, *put1* and *put2* in YPD medium grown in a 96-well microplate for 20 h. Data is presented as mean ± SD (n = 3). (Inset) The *put1* well showed aggregated cells which is reflected in the erratic reading in the saturated phase. (**H**) Intracellular ATP of *put* mutants entering the saturated phase is lower than the wildtype. ATP was extracted from cells grown for 16 h in liquid YPD as in (**E**). ATP concentrations presented were normalized to total protein content (mean with 95% CI; **p <0.01, *p <0.05 by one-way ANOVA with Dunnett’s posthoc test). (**I**) Propidium iodide (PI) staining of cells from 48 h-old cultures as in (**E**). Results are representative of at least three independent experiments. Strains used: WT (SC5314); *put1* (CFG154), *put2* (CFG318); *put3* (CFG156); *put1 put2* (CFG159); *gdh2* (CFG279); *gdh2 put1* (CFG364); *gdh2 put2* (CFG366).
(TIF)

S5 Fig Expression of Proline Utilization enzymes during growth in the presence of different protein sources. CFG433 cells from SGL were inoculated into a modified HBSS medium containing glucose and lactate plus the indicated protein as sole nitrogen source (0.5 mg/ml) and incubated at 37°C for 24 h (**A**) and 72 h (**B**). Cell extracts were prepared and analyzed by immunoblot and developed to detect Put1 and Put2 as indicated. Legend: Am (Ammonium sulfate), Coll (Collagen), Hb (Hemoglobin), Muc (Mucin), Alb (Human serum albumin). Results (mean±SD, n = 4) were analyzed by one-way ANOVA with Dunnett’s posthoc test relative to Am (****p <0.0001). Proline derived from mucin appears to be easily assimilated by *C*. *albicans*.
(TIF)

S6 Fig Mutations inactivating proline catabolism attenuate virulence in BALB/cAnNCrl mice. Upper panel, female BALB/cAnNCrl mice were infected via the lateral tail vain with 5x10^5^ CFU of *C*. *albicans* wildtype (SC5314) or *put2* (CFG318) mutant. Each curve in the plot is the average of 3 independent experiments (10 mice/strain). Mice infected with *put2-/-* survived longer compared to wildtype (****p<0.0001 by Log-rank (Mantel-Cox) test). Lower panel, the fungal burden in kidneys extracted from mice 2 days after infection. Box and whiskers plot showing significantly lower fungal burden in the kidney of mice infected with *put2* mutant compared to wildtype (**p = 0.0039 by student *t-*test).
(TIF)

S7 Fig Filamentation of wiltype and *put2* strains in DMEM+10% FBS. Wildtype (PLC096) and *put2* (CFG479) cells from a YPD pre-culture were stained with FITC and added to 2 ml of regular DMEM (high glucose) medium with 10% FBS and penicillin/streptomycin (D10) to induce hyphal growth for 3 h and photographed. The images shown were merged FITC and yEmRFP channels.
(TIF)

S1 Table Key reagents and resources. (DOCX)

S2 Table Oligonucleotides. (DOCX)

S1 Movie Animation of a 3D reconstruction of an intravitally acquired z-stack through the renal cortex of a BALB/cByJ SOPF mouse, 24h p.i. with wildtype *C*. *albicans*, expressing yEmRFP and stained with FITC, merged channels: yEmRFP (red), FITC (green), Autofluorescence of NAD(P)H (teal) (as in Fig 6F, left). (MP4)

S2 Movie Animation of a 3D reconstruction of an *ex vivo* acquired z-stack through the renal cortex of a BALB/cByJ SOPF mouse, 24h p.i. with wildtype *C*. *albicans*, expressing yEmRFP and stained with FITC, merged channels: yEmRFP (red), FITC (green), Autofluorescence of NAD(P)H (teal). (MP4)

S3 Movie Animation of a 3D reconstruction of an intravitally acquired z-stack through the renal cortex of a BALB/cByJ SOPF mouse, 24h p.i. with *put2 C*. *albicans*, expressing yEmRFP and stained with FITC, merged channels: yEmRFP (red), FITC (green), Autofluorescence of NAD(P)H (teal) (as in Fig 6F, right). (MP4)

S4 Movie Animation of a 3D reconstruction of an *ex vivo* acquired z-stack through the renal cortex of a BALB/cByJ SOPF mouse, 24h p.i. with *put2 C*. *albicans*, expressing yEmRFP and stained with FITC, merged channels: yEmRFP (red), FITC (green), Autofluorescence of NAD(P)H (teal). (MP4)
