# Supplementary figures and images for: Proline catabolism is a key factor facilitating Candida albicans pathogenicity
Source: PLoS Pathog. 2023 Nov 2;19(11):e1011677. doi: 10.1371/journal.ppat.1011677 (PMC10621835; doi:10.1371/journal.ppat.1011677)

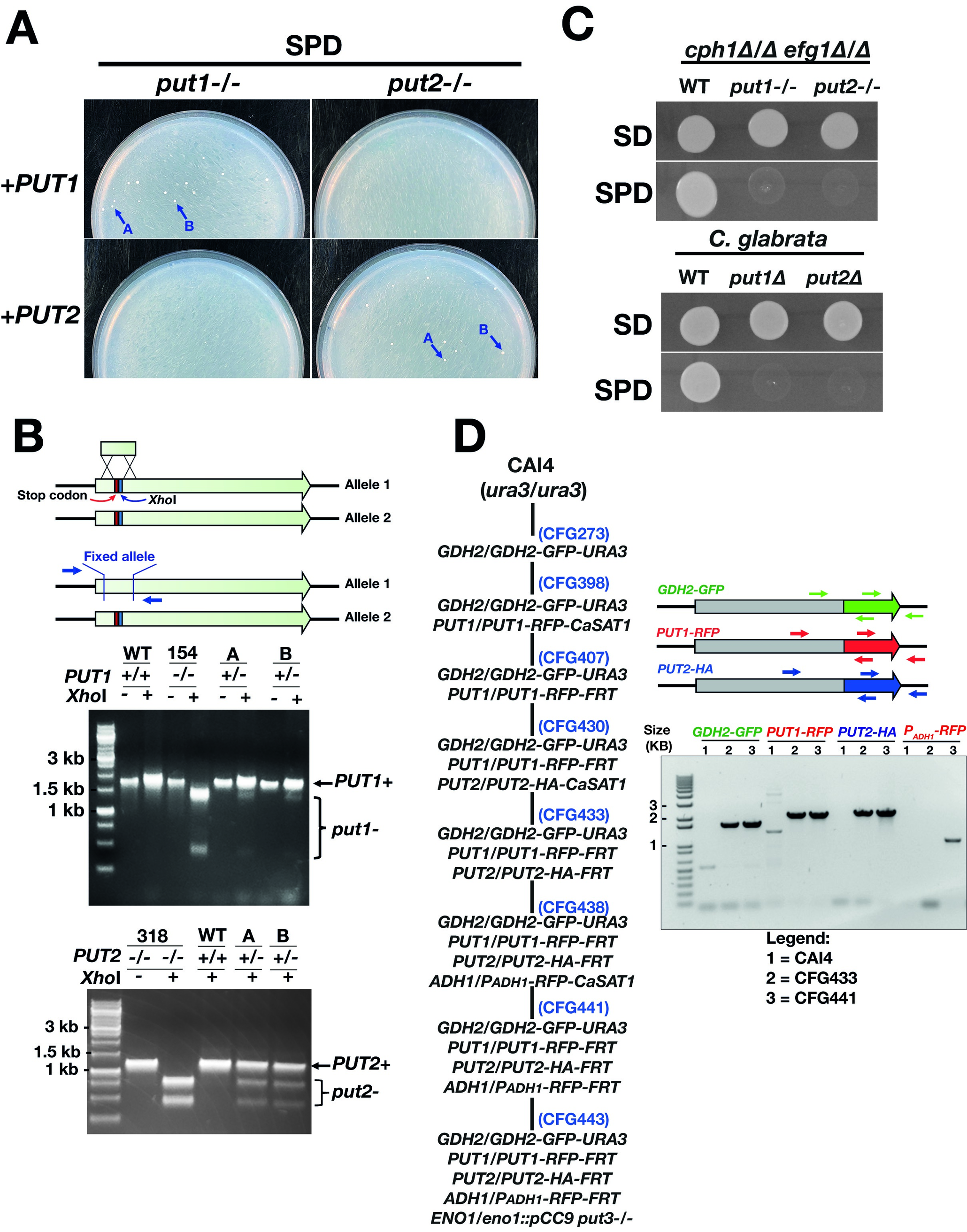

Supplement: S1 Fig — (A) Targeted reconstitution: put1-/- (CFG154) and put2-/- (CFG318) strains were transformed with wildtype PUT1 and PUT2 fragments, respectively, and proline-utilizing (Put+) colonies were selected on SPD. As control, the PUT2 and PUT1 fragments were introduced into the put1-/- and put2-/- strains, respectively; no transformants were obtained. (B) Verification of the reconstructed PUT1 and PUT2 alleles. The colonies with arrows (A, B) were purified and their genomes analyzed by PCR-RD. The heterozygosity at the indicated gene locus was confirmed, i.e., PUT1+/- (PUT1+/put1-) and PUT2+/- (PUT2+/put2-). Primers (shown in blue) facilitate the amplification of both the wildtype Alleles 1 and XhoI containing CRISPR/Cas9 inactivated Alleles 2. The amplified fragments were digested with XhoI and fragment lengths were analyzed by electrophoresis (1% agarose gel). The fragments with the inactivated alleles are cleaved by XhoI resulting in two bands (indicated by the brackets), whereas the reconstructed wildtype fragment is refractory to XhoI digestion and runs as a single band (arrow). (C) C. albicans cph1Δ/Δ efg1Δ/Δ (WT, CASJ041), cph1Δ/Δ efg1Δ/Δ put1-/- (CFG344), cph1Δ/Δ efg1Δ/Δ put2-/- (CFG345) and C. glabrata (WT, CBS138), Cgput1Δ (GFS003), Cgput2Δ (GFS005) strains were spotted on non-selective (SD) and selective (SPD) media as indicated. The inactivation of PUT genes resulted in the lack of growth on selective media, indicating that inability of the mutants to use catabolize proline. (D) Schematic diagram of steps required to construct the triply-tagged reporter strain. The starting parental strain was CAI4 (ura3/ura3); the complete genotypes of the strains are listed in Methods. PCR-verification of reporter constructs used two pairs of primer pairs described in Methods. Sizes of fragments amplified with primer pairs flanking the tag inserts: GDH2-GFP (1.6 kb), PUT1-RFP (2.1 kb), PUT2-HA (2.3 kb), PADH1-RFP (1.2 kb). (TIF) [file ppat.1011677.s001.tif]

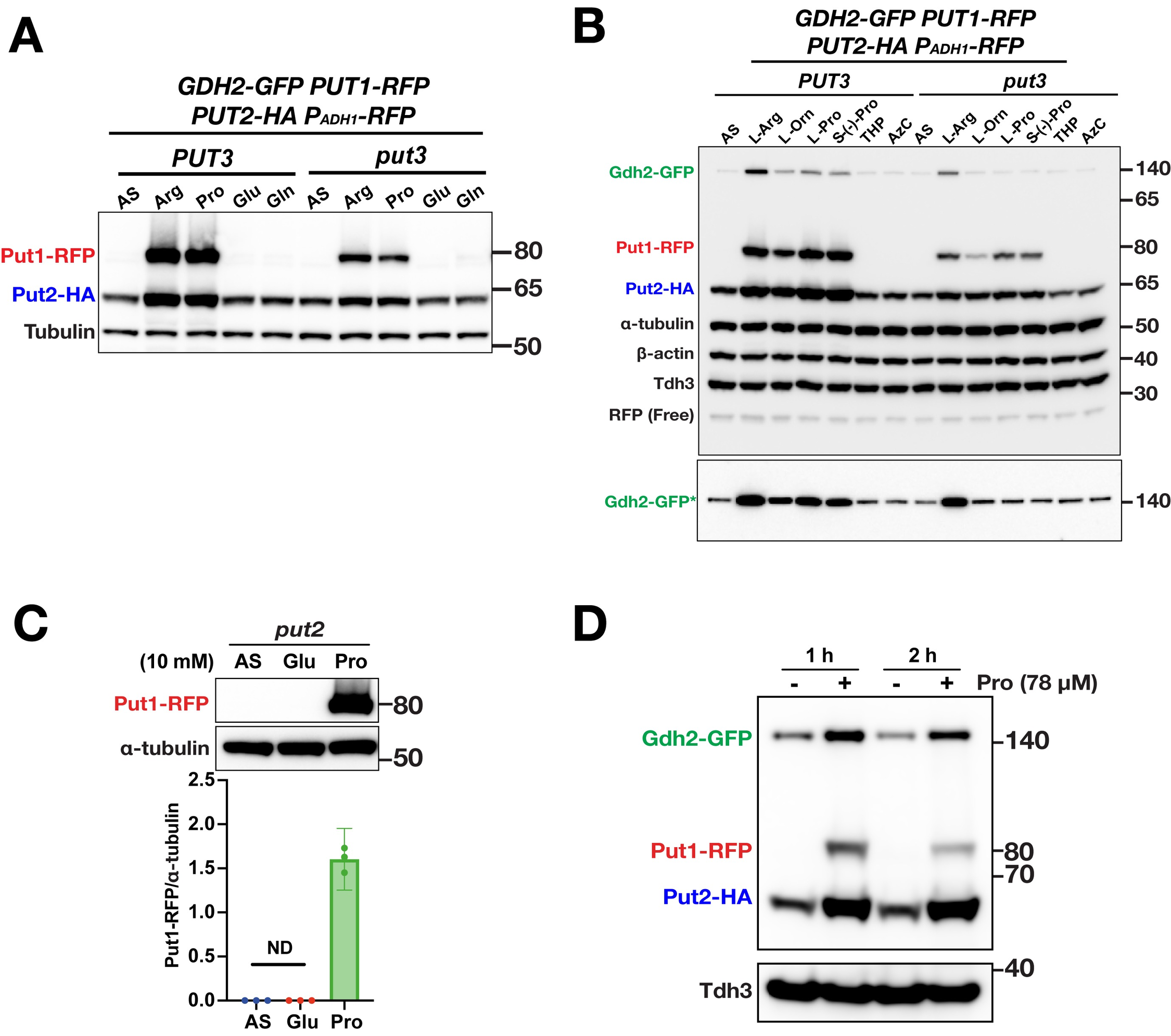

Supplement: S2 Fig — (A) Exponentially growing cultures of triple tagged reporter strains CFG441 (PUT3) and CFG443 (put3) in SGL were induced with 10 mM of the indicated nitrogen source for 1 h. Cell extracts were prepared and the expression of the Put1, Put2 and tubulin was analyzed by immunoblot using an optimized antibody cocktail (see Methods). Note that Put1-RFP is not detected in cultures containing either glutamate or glutamine. (B) Specificity of Put3 to proline. Strains, growth and analysis as in (A) of cultures induced with the addition of 10 mM of the indicated compounds: Ammonium sulfate (Am); L-ornithine; L-arginine; L-proline; S-(-)-proline; T-4-hydroxy-L-proline; and Azetidine carboxylate (AzC). Gdh2-GFP signals (*) were separately enhanced (lower panel) via the high slider in Image Lab (BioRad). (C) Glutamate is not readily metabolized to proline. Strain CFG469 was grown and induced as in (A) with 10 mM ammonium sulfate (Am), glutamate or proline as indicated. Note that Put1-RFP was not detected (ND) in Am or glutamate induced cultures. The immunoblots are representative of at least three independent experiments. (D) PUT enzyme expression is sensitive to low inducing levels of proline. Cells were grown as in (B) but induced with 78 μM proline for 1 and 2 h. The blot shown is representative of at least 3 independent experiments. (TIF) [file ppat.1011677.s002.tif]

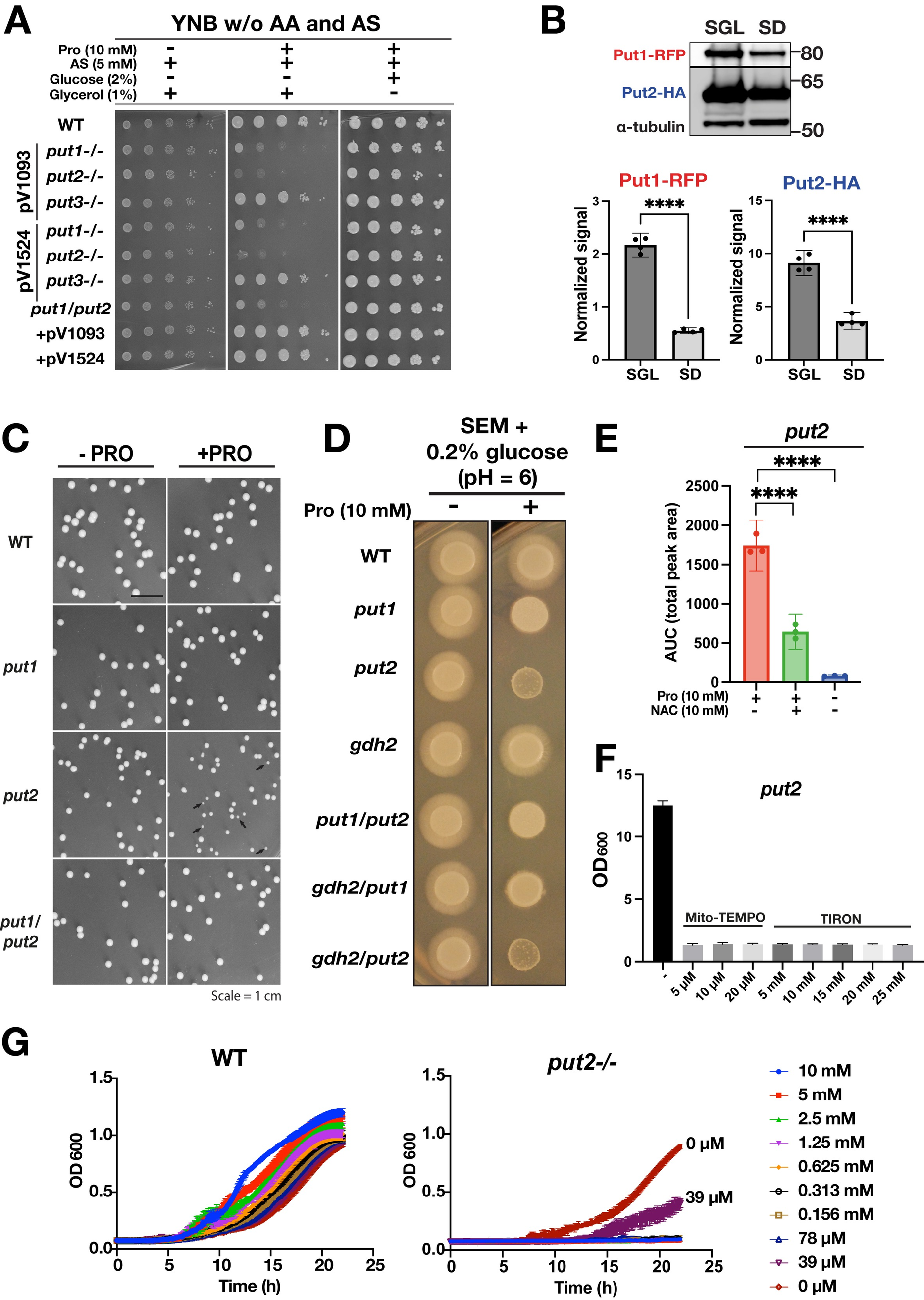

Supplement: S3 Fig — (A) Serially diluted C. albicans cells of the indicated genotypes (same as in Fig 1C) were spotted onto buffered synthetic minimal medium (pH = 6) containing 5 mM ammonium sulfate (Am) as nitrogen source and either 1% glycerol or 2% glucose as carbon source. Excess proline (10 mM) was added as indicated. Plates were photographed after 4 days of growth at 30°C. (B) Strain CFG433, grown to log phase in SGL or SD, was induced with 10 mM proline for 1 h, and cell extracts were analyzed by immunoblot. The signals from Put1-RFP and Put2-HA were normalized to α-tubulin. Data presented are from 4 biological replicates (Ave. with 95% CI; ****p<0.0001 by student t-test). (C) Cells from 72 h-old SD cultures of the indicated strains (same as in Fig 3A) were plated for single colonies on YPD and grown for 2 days. The colonies from the put2 mutant are heterogenous in size, both large and small colonies (black arrows) are evident. Images were representative of at least 3 biological replicates. Scale 1 = cm. (D) Five μl of cell suspensions of the indicated strains (same as in Fig 3A) were spotted on buffered SEM medium (pH = 6) containing 10 mM glutamate with and without 10 mM proline as indicated. The resulting macrocolonies were photographed after 72 h at 30°C. (E) N-acetylcysteine (NAC) reduced ROS in put2 cells treated with proline. Experiments were performed as in Fig 3C but 30 min prior to reading the luminol-HRP signal, 10 mM NAC was added to cultures to sequester ROS. Data are presented as mean with 95% CI (n = 3). (F) ROS (superoxide) scavengers failed to rescue the growth of put2 in the presence of exogenous proline. CFG318 was grown as in Fig 3D with and without the indicated concentrations of Mito-TEMPO or TIRON. The results are the average of at 4 biological replicates (with 95% CI). (G) Growth of WT (SC5314) and put2 (CFG318) strains in SGL in a 96-well microplate in the presence of the indicated concentrations of proline. Data is presented as mean ± SD (n = 4). (TIF) [file ppat.1011677.s003.tif]

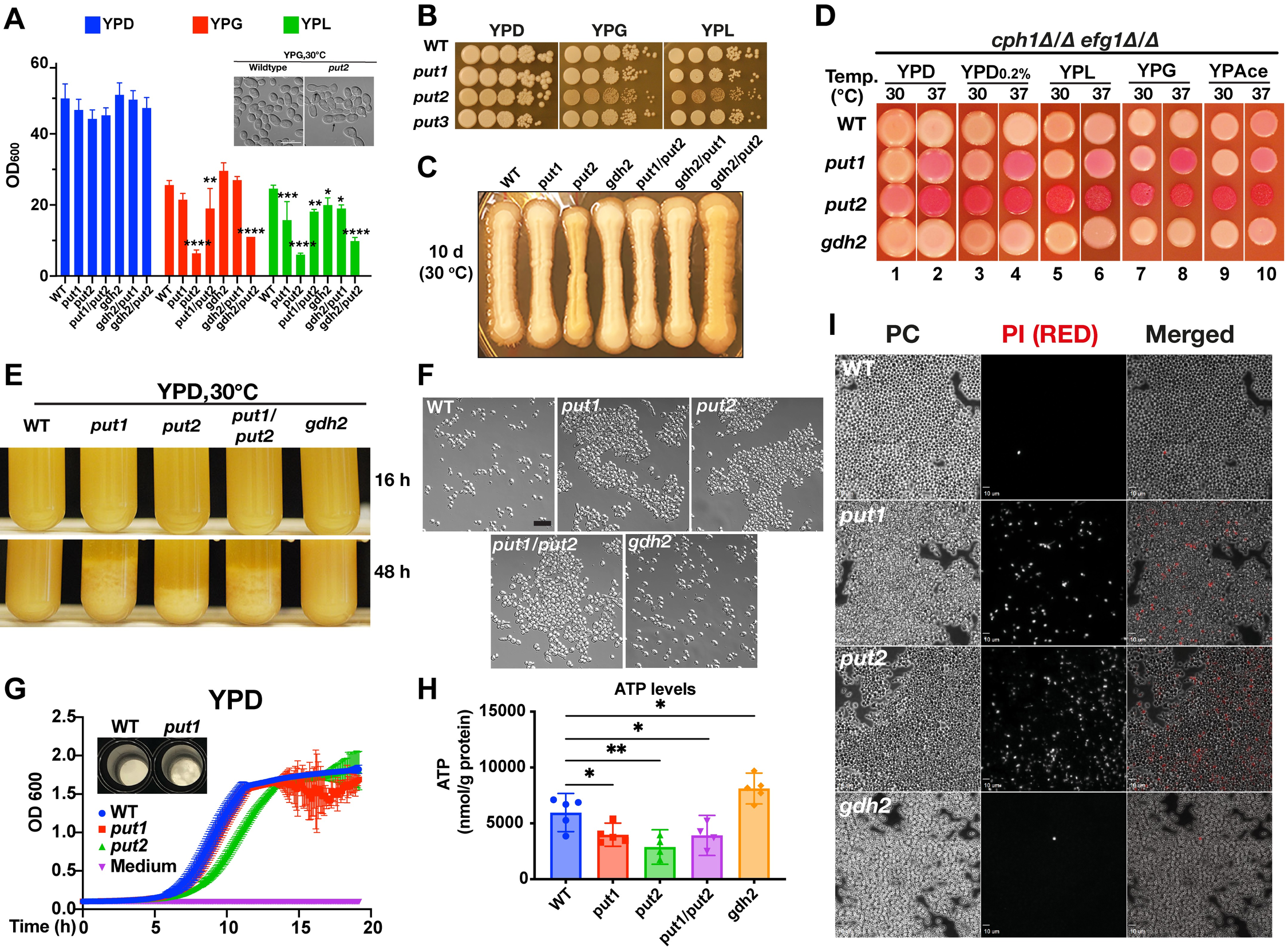

Supplement: S4 Fig — (A) Growth of put mutants after 24 h in the indicated media containing different carbon sources [2% glucose (YPD), 1% glycerol (YPG), 1% lactate (YPL)]. Note that put2 cells grown in YPG have noticeably higher number of cells forming trimera a phenotype indicative of stress (black arrows, inset). Each bar represents the mean ±SD (n = 4). Results per carbon source were analyzed by one-way ANOVA with Dunnett’s posthoc test relative to wildtype (****p <0.0001, ***p <0.001, **p <0.01, *p <0.05). (B) Ten-fold serial dilutions of cell suspensions (OD600 ≈ 1) were spotted on YPD, YPG or YPL grown for 2 days at 30°C. (C) Strains were streaked on YPD and grown for 10 days at 30°C. Note the increase in yellow hue in put2 and gdh2 put2 strains. (D) Five microliters of cph1Δ/Δ efg1Δ/Δ (CASJ041) or its derivatives (put1, CFG344; put2, CFG345; gdh2, CFG352) were spotted on the indicated plates containing Phloxine B and incubated for 3 days at 30- or 37-°C. (E) Flocculation in YPD cultures was assessed after 16 and 48 h of growth at 30°C. Cultures were vigorously vortexed and let stand immobile for 3 min and photographed. (F) Microscopic inspection of 16 h-old cultures as in (E) showing onset of flocculation in put mutants (Scale bar = 10 μm). (G) Growth curves of WT, put1 and put2 in YPD medium grown in a 96-well microplate for 20 h. Data is presented as mean ± SD (n = 3). (Inset) The put1 well showed aggregated cells which is reflected in the erratic reading in the saturated phase. (H) Intracellular ATP of put mutants entering the saturated phase is lower than the wildtype. ATP was extracted from cells grown for 16 h in liquid YPD as in (E). ATP concentrations presented were normalized to total protein content (mean with 95% CI; **p <0.01, *p <0.05 by one-way ANOVA with Dunnett’s posthoc test). (I) Propidium iodide (PI) staining of cells from 48 h-old cultures as in (E). Results are representative of at least three independent experiments. Strains used: WT (SC5314); put1 (CFG154 [file ppat.1011677.s004.tif]

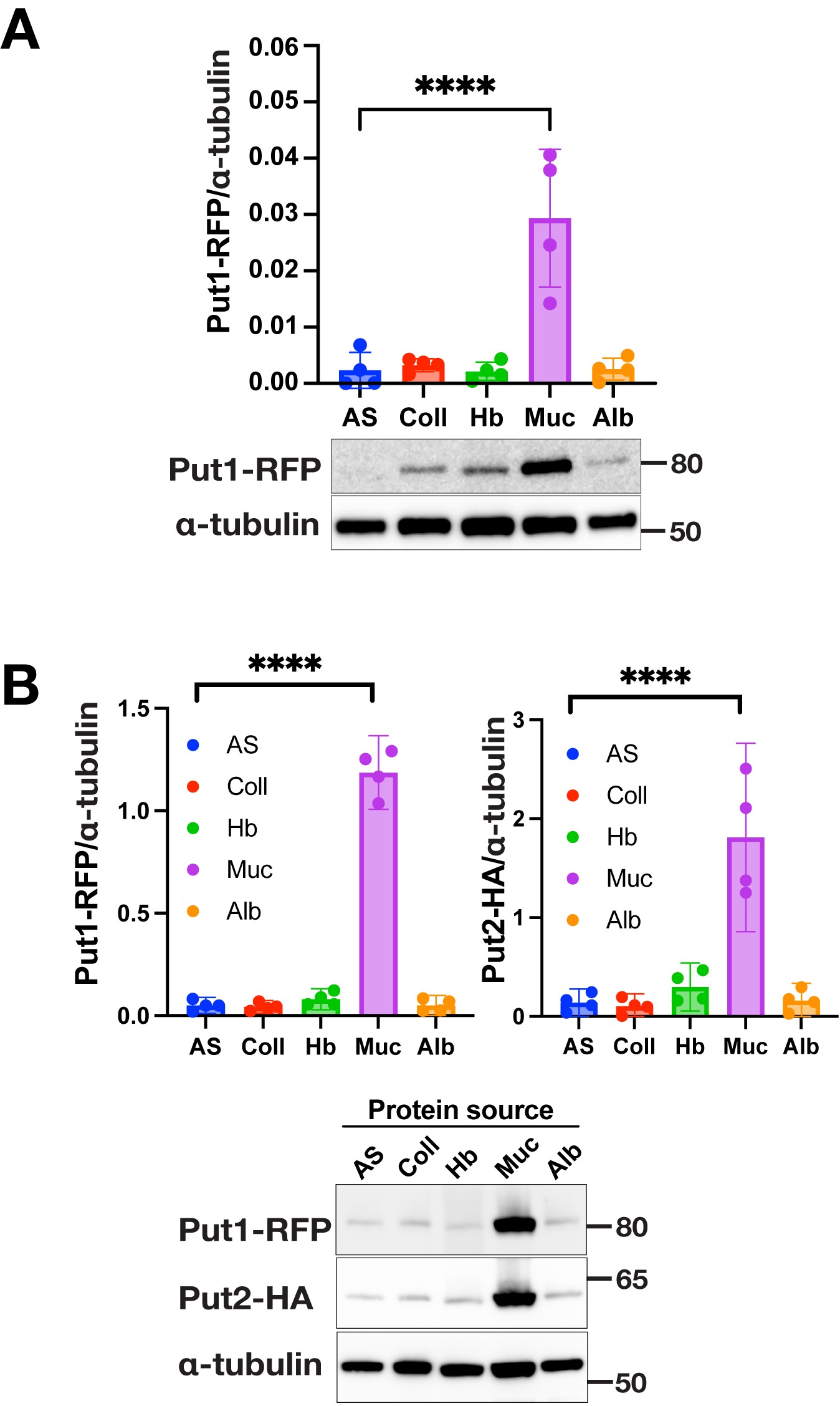

Supplement: S5 Fig — CFG433 cells from SGL were inoculated into a modified HBSS medium containing glucose and lactate plus the indicated protein as sole nitrogen source (0.5 mg/ml) and incubated at 37°C for 24 h (A) and 72 h (B). Cell extracts were prepared and analyzed by immunoblot and developed to detect Put1 and Put2 as indicated. Legend: Am (Ammonium sulfate), Coll (Collagen), Hb (Hemoglobin), Muc (Mucin), Alb (Human serum albumin). Results (mean±SD, n = 4) were analyzed by one-way ANOVA with Dunnett’s posthoc test relative to Am (****p <0.0001). Proline derived from mucin appears to be easily assimilated by C. albicans. (TIF) [file ppat.1011677.s005.tif]

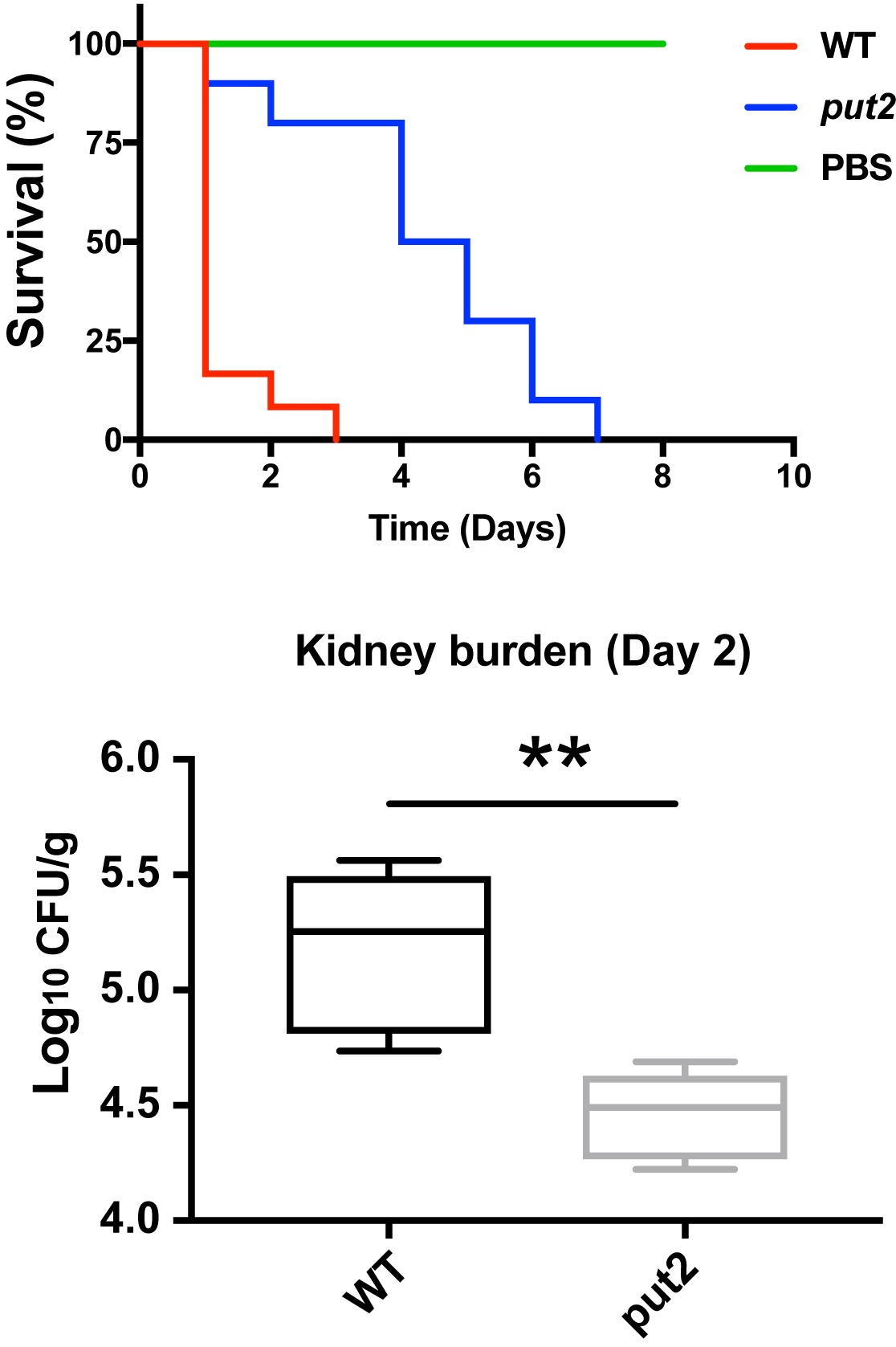

Supplement: S6 Fig — Upper panel, female BALB/cAnNCrl mice were infected via the lateral tail vain with 5x10^5^ CFU of C. albicans wildtype (SC5314) or put2 (CFG318) mutant. Each curve in the plot is the average of 3 independent experiments (10 mice/strain). Mice infected with put2-/- survived longer compared to wildtype (****p<0.0001 by Log-rank (Mantel-Cox) test). Lower panel, the fungal burden in kidneys extracted from mice 2 days after infection. Box and whiskers plot showing significantly lower fungal burden in the kidney of mice infected with put2 mutant compared to wildtype (**p = 0.0039 by student t-test). (TIF) [file ppat.1011677.s006.tif]

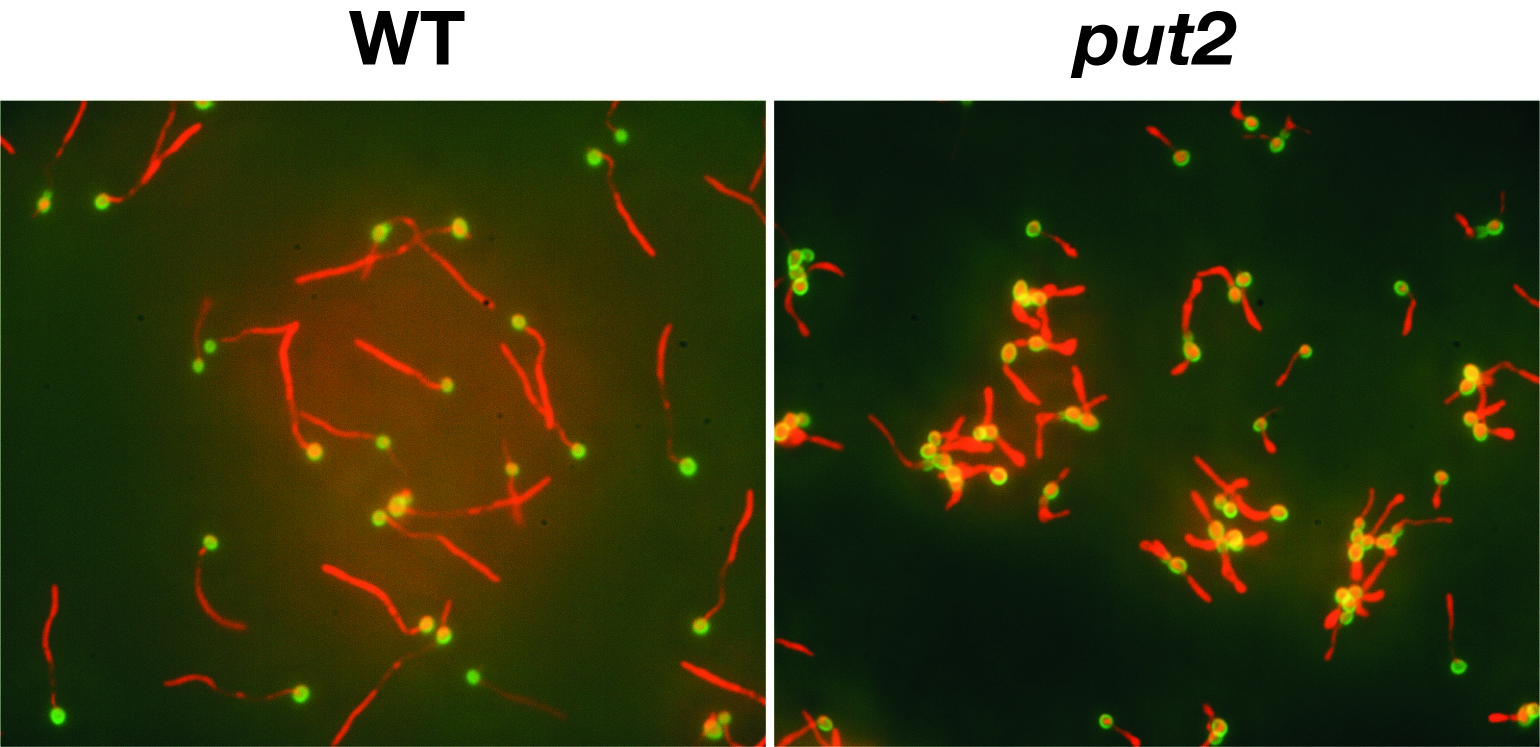

Supplement: S7 Fig — Wildtype (PLC096) and put2 (CFG479) cells from a YPD pre-culture were stained with FITC and added to 2 ml of regular DMEM (high glucose) medium with 10% FBS and penicillin/streptomycin (D10) to induce hyphal growth for 3 h and photographed. The images shown were merged FITC and yEmRFP channels. (TIF) [file ppat.1011677.s007.tif]
