# Supplementary material for: Proline catabolism is a key factor facilitating Candida albicans pathogenicity
Source: PLoS Pathog. 2023 Nov 2;19(11):e1011677. doi: 10.1371/journal.ppat.1011677 (PMC10621835; doi:10.1371/journal.ppat.1011677)
Supplement: S1 Table — (DOCX) [file ppat.1011677.s008.docx]

| **S1 Table. Key reagents and resources** | | |
| --- | --- | --- |
| **Reagent or Resource** | **Source** | **Identifier (Catalogue # or genotype)** |
| **Antibodies** | | |
| Anti-ATP5A [EPR13030(B)], rabbit monoclonal | Abcam | Cat#ab176569 |
| Anti-β-actin, mouse monoclonal | Abcam | Cat#ab8224 |
| Anti-GFP, Living Colors A.v. (JL-8), mouse monoclonal | Takara | Cat#632381 |
| Anti-HA-Peroxidase, High Affinity (clone 3F10) rat monoclonal | Roche | Cat#12013819001 |
| Anti-mCherry, rabbit polyclonal | Abcam | Cat#ab167453 |
| Anti-GAPDH [GT239], mouse monoclonal | Genetex | Cat#GTX627408 |
| Anti-tubulin [YOL1/34] conjugated to HRP, rat monoclonal | Abcam | Cat#ab196583 |
| Goat anti-mouse IgG (H+L) secondary antibody, poly-HRP | Invitrogen | Cat#31430 |
| Goat anti-rabbit IgG (H+L) secondary antibody, poly-HRP | Invitrogen | Cat#31460 |
| **Chemicals, peptides, and recombinant proteins** | | |
| 2-aminobenzaldehyde (O-ABZ) | Sigma-Aldrich | Cat#A9628 |
| Agar | Formedium | Cat#A9628 |
| Albumin from human serum | Sigma-Aldrich | Cat#A1653 |
| Ammonium sulfate | VWR | Cat#21333.296 |
| Carbenicillin disodium salt | AppliChem GmbH | Cat#A1491 |
| cis-4-Hydroxy-L-proline | Sigma-Aldrich | Cat#H1637 |
| cis-4-Hydroxy-D-proline | Sigma-Aldrich | Cat#H5877 |
| Collagen from bovine achilles tendon | Sigma-Aldrich | Cat#C9879 |
| Collagen from human placenta Bornstein and Traub Type IV | Sigma-Aldrich | Cat#C7521 |
| cOmplete Mini, EDTA free (protease inhibitor cocktail) | Roche | Cat#11836170001 |
| D-(+)-Glucose | Sigma-Aldrich | Cat#G7528 |
| D-Proline | Sigma-Aldrich | Cat#858919 |
| DL-Lactic acid, ~90% (T) | Sigma-Aldrich | Cat#69785 |
| DMEM high glucose | Thermo Fisher Scientific | Cat#11965092 |
| DMEM SILAC Flex medium | Thermo Fisher Scientific | Cat#A2493901 |
| DTT - DL-1,4-Dithiothreitol | Acros Organics | Cat#165680050 |
| DreamTaq Green DNA Polymerase | Thermo Fisher Scientific | Cat#EP0702 |
| ExTaq Hot Start Version (250U) | Takara | Cat#RR006A |
| Fastdigest *Kpn*I | Thermo Fisher Scientific | Cat#FD0524 |
| Fastdigest *Nco*I | Thermo Fisher Scientific | Cat#FD0573 |
| Fastdigest *Pvu*I | Thermo Fisher Scientific | Cat#FD0624 |
| Fastdigest *Sac*I | Thermo Fisher Scientific | Cat#FD1133 |
| Fastdigest *Xho*I | Thermo Fisher Scientific | Cat#FD0694 |
| FITC - Fluorescein-5-isothiocyanate | Sigma-Aldrich | Cat#F7250 |
| Gibson Assembly Master Mix | New England Biolabs | Cat# E2611 |
| Glycerol, ≥99.0% (GC) | Sigma-Aldrich | Cat#G7757 |
| Hemoglobin from bovine blood | Fluka | Cat#8449 |
| Histopaque-1119 | Sigma-Aldrich | Cat# 11191 |
| Isoflurane, Attane vet 1000 mg/g | Piramal Healthcare UK Ltd. | Cat#NDC66794017-25 |
| L-Arginine monohydrochloride | Sigma-Aldrich | Cat#A5131 |
| L-Azetidine-2-Carboxylic acid | Sigma-Aldrich | Cat#A0760 |
| L-Glutamic acid | Sigma-Aldrich | Cat# G1251 |
| L-Glutamine | Sigma-Aldrich | Cat#G3126 |
| L-Ornithine | Sigma-Aldrich | Cat#O2375 |
| L-Proline | Sigma-Aldrich | Cat#P0380 |
| Luminol | Sigma-Aldrich | Cat#123072 |
| MitoSOX Red | Invitrogen | Cat#M36008 |
| MitoTEMPO | Sigma-Aldrich | Cat#SML0737 |
| Mucin from porcine stomach Type II | Sigma-Aldrich | Cat#M2378 |
| N-acetyl-cysteine (NAC) | Sigma-Aldrich | Cat#A7250 |
| Nourseothricin (clonNAT) | Jena Bioscience | Cat#AB-102XL |
| NuPAGE 4 to 12%, Bis-Tris, 1.0–1.5 mm, Mini Protein Gels | Thermo Fisher Scientific | Cat#NP0322BOX |
| NuPAGE 3 to 8%, Tris-Acetate, 1.0 mm, Mini Protein Gel | Thermo Fisher Scientific | Cat#EA03752BOX |
| NuPAGE MES SDS Buffer (20X) | Thermo Fisher Scientific | Cat#NP0002 |
| NuPAGE MOPS SDS Buffer (20X) | Thermo Fisher Scientific | Cat#NP0001 |
| NuPAGE Tris-Acetate SDS Running Buffer (20X) | Thermo Fisher Scientific | Cat#LA0041 |
| Peptone | Formedium | Cat#PEP03 |
| Peptone | OXOID | Cat#PEP03 |
| Peroxidase from horseradish | Sigma-Aldrich | Cat#P8125-5K |
| Phloxine B, 85%, high purity biological stain | VWR | Cat#ACRO189470050 |
| Phusion High-Fidelity DNA Polymerase | Thermo Fisher Scientific | Cat#F530L |
| Propidium iodide (1.0 mg/ml in H_2_O) | Thermo Fisher Scientific | Cat#P3566 |
| PureCol EZ gel | Sigma-Aldrich | Cat#5074 |
| (S)-(-)-Proline | Sigma-Aldrich | Cat#8160190025 |
| Sabouraud Dextrose Agar Medium | ACMEC biochemical | Cat#AC15825 |
| SDS - Sodium dodecyl sulfate | Sigma-Aldrich | Cat#75746 |
| SuperSignal Dura West Extended Duration Substrate | Thermo Fisher Scientific | Cat#34076 |
| Thiazolidine-2-carboxylic acid | Sigma-Aldrich | Cat#467995 |
| TIRON - 4,5-Dihydroxy-1,3-benzenedisulfonic acid disodium salt monohydrate | Sigma-Aldrich | Cat#172553 |
| trans-4-Hydroxy-L-proline | Sigma-Aldrich | Cat#H54409 |
| Yeast extract powder | Formedium | Cat#YEA02 |
| Yeast extract powder | OXOID | Cat#LP0021 |
| Yeast Nitrogen Base without amino acids and ammonium sulfate | BD Difco | Cat#11743014 |
| **Critical commercial assays** | | |
| Molecular Probes  ATP Determination Kit | Thermo Fisher Scientific | Cat#A22066 |
| GeneJet PCR Purification Kit | Thermo Fisher Scientific | Cat#K0702 |
| GeneJet Plasmid Miniprep Kit | Thermo Fisher Scientific | Cat#K0503 |
| Masterpure Yeast DNA Purification Kit | Epicentre | Cat#MPY80200 |
| QIAquick Gel Extraction Kit | Qiagen | Cat#28706 |
| **Recombinant DNA** |  |  |
| Plasmid: pFA-GFPγ-URA3 | [1] | pFA-GFPγ-URA3 |
| Plasmid: pFA6a-3xHA-SAT1-FLP | [2] | pFA6a-3xHA-SAT1-FLP |
| Plasmid: pJA21 | [3] | *P_ADH1_-RFP-CaSAT1* flipper cassette |
| Plasmid: pV1093 | [4] | CRISPR/Cas9 cassette, general vector |
| Plasmid: pV1524 | [5] | CRISPR/Cas9 cassette, general vector |
| Plasmid: pFS080 | [3] | *PUT1* sgRNA inserted into pV1093 |
| Plasmid: pFS083 | [3] | *PUT2* sgRNA inserted into pV1093 |
| Plasmid: pFS084 | [3] | *PUT3* sgRNA inserted into pV1093 |
| Plasmid: pFS088 | [3] | *PUT1* sgRNA inserted into pV1524 |
| Plasmid: pFS092 | [3] | *PUT2* sgRNA inserted into pV1524 |
| Plasmid: pFS090 | [3] | *PUT3* sgRNA inserted into pV1524 |
| Plasmid: pSFS3b | [6] | Donor plasmid for SAT1 flipper fragment used for Gibson assembly |
| Plasmid: YEp352 | [7] | Donor plasmid for *E. coli* Ori and Amp^R^ fragment for Gibson assembly |
| Plasmid: YEp352-*NAT1*-Cg*PUT1*urdr | This study | Plasmid for *C. glabrata* *PUT1* gene deletion |
| Plasmid: YEp352-*NAT1*-Cg*PUT2*urdr | This study | Plasmid for *C. glabrata* *PUT2* gene deletion |
| **Experimental models: Organisms/Strains** | | |
| **Mouse strains** | | |
| C57BL/6 | Zhejiang Vital River Laboratory Animal Technology Co., Ltd. |  |
| BALB/cAnNCrl | Charles River, Germany |  |
| BALB/cByJ SOPF | Charles River, France |  |
| ***Drosophila melanogaster*** **strains** | | |
| *Bom^Δ55C^* | [8] | Toll-regulated Bomanin effectors-deficient flies |
| ***C. albicans* CAI4-derived strains** | | |
| *C. albicans:* PLC016, PMRCA18 | [9] | *ura3::imm434/ura3::URA3* |
| *C. albicans:* CFG219 | [10] | *ura3::imm434/ura3::imm434 iro1/iro1::imm434 PUT2/PUT2-GFP-URA3* |
| *C. albicans:* CFG237 | [10] | *ura3::imm434/ura3::imm434 iro1/iro1::imm434 PUT2/PUT2-GFP-URA3 ADH1/adh1::P_ADH1_*-*RFP-CaSAT1* |
| *C. albicans:* CFG259 | [10] | *ura3::imm434/ura3::imm434 iro1/iro1::imm434 PUT2/PUT2-GFP-URA3 ADH1/adh1::P_ADH1_*-*RFP-FRT* |
| *C. albicans:* CFG301 | This work | *ura3::imm434/ura3::imm434 iro1/iro1::imm434 PUT2/PUT2-GFP-URA3 ADH1/adh1::P_ADH1_*-*RFP-FRT ENO1/eno1::P_ENO1_-CC9-pFS084 put3-/-* |
| *C. albicans:* CFG407 | [10] | *ura3::imm434/ura3::imm434 iro1/iro1::imm434 GDH2/GDH2-GFP-URA3 PUT1/PUT1-RFP-FRT-FRT* |
| *C. albicans:* CFG430 | This work | *ura3::imm434/ura3::imm434 iro1/iro1::imm434 GDH2/GDH2-GFP-URA3 PUT1/PUT1-RFP-FRT-FRT PUT2/PUT2-HA-CaSAT1* |
| *C. albicans:* CFG433 | This work | *ura3::imm434/ura3::imm434 iro1/iro1::imm434 GDH2/GDH2-GFP-URA3 PUT1/PUT1-RFP-FRT-FRT PUT2/PUT2-HA-FRT* |
| *C. albicans:* CFG438 | This work | *ura3::imm434/ura3::imm434 iro1/iro1::imm434 GDH2/GDH2-GFP-URA3 PUT1/PUT1-RFP-FRT-FRT PUT2/PUT2-HA-FRT ADH1/adh1::P_ADH1_*-*RFP-CaSAT1* |
| *C. albicans:* CFG441 | This work | *ura3::imm434/ura3::imm434 iro1/iro1::imm434 GDH2/GDH2-GFP-URA3 PUT1/PUT1-RFP-FRT-FRT PUT2/PUT2-HA-FRT ADH1/adh1::P_ADH1_*-*RFP-FRT* |
| *C. albicans:* CFG443 | This work | *ura3::imm434/ura3::imm434 iro1/iro1::imm434 GDH2/GDH2-GFP-URA3 PUT1/PUT1-RFP-FRT-FRT PUT2/PUT2-HA-FRT ADH1/adh1::P_ADH1_*-*RFP-FRT ENO1/eno1*::*P_ENO1_-CC9-pFS084 put3-/-* |
| ***C. albicans* SC5314-derived strains** | | |
| *C. albicans*: SC5314, PLC005 | [11] | Prototrophic wildtype |
| *C. albicans*: CASJ041 | [12]; KK Collection | *cph1Δ::FRT/ cph1Δ::FRT efg1Δ::FRT/efg1Δ::FRT* |
| *C. albicans*: CFG143 | [3] | *ENO1/eno1::P_ENO1_-CC9-pFS083 put2-/-* |
| *C. albicans*: CFG146 | [3] | *NEUT5/neut5::P_ENO1_-CC9-pFS090 put3- /-* |
| *C. albicans*: CFG149 | [3] | *ENO1/eno1::P_ENO1_-CC9-pFS080 put1-/-* |
| *C. albicans*: CFG150 | [3] | *ENO1/eno1::P_ENO1_-CC9-pFS084 put3-/-* |
| *C. albicans*: CFG154 | [3] | *NEUT5/neut5::FRT put1-/-* |
| *C. albicans:* CFG156 | [3] | *NEUT5/neut5::FRT put3-/-* |
| *C. albicans*: CFG159 | [3] | *NEUT5/neut5::FRT put1-/- ENO1/eno1::P_ENO1_-CC9-pFS083 put2- /-* |
| *C. albicans*: CFG181 | [3] | *ENO1/eno1::P_ENO1_-CC9-pV1093* |
| *C. albicans*: CFG182 | [3] | *NEUT5/neut5::P_ENO1_-CC9-pV1524* |
| *C. albicans*: CFG187 | This work | *PUT3/PUT3-HA-CaSAT1 (Clone 1)* |
| *C. albicans*: CFG188 | This work | *PUT3/PUT3-HA-CaSAT1 (Clone 2)* |
| *C. albicans*: CFG279 | [10] | *NEUT5/neut5::FRT gdh2- /-* |
| *C. albicans*: CFG318 | [3] | *NEUT5/neut5::FRT put2- /-* |
| *C. albicans*: CFG344 | This work | *cph1Δ::FRT/cph1Δ::FRT efg1Δ::FRT/efg1Δ::FRT NEUT5/neut5::FRT put1-/-* |
| *C. albicans*: CFG345 | This work | *cph1Δ::FRT/cph1Δ::FRT efg1Δ::FRT/efg1Δ::FRT NEUT5/neut5::FRT put2-/-* |
| *C. albicans*: CFG352 | [10] | *cph1Δ::FRT/cph1Δ::FRT efg1Δ::FRT/efg1Δ::FRT NEUT5/neut5::FRT gdh2-/-* |
| *C. albicans*: CFG364 | This work | *NEUT5/neut5::FRT gdh2-/- ENO1/eno1::P_ENO1_-CC9-pFS080 put1-/-* |
| *C. albicans*: CFG366 | This work | *NEUT5/neut5::FRT gdh2-/- ENO1/eno1::P_ENO1_-CC9-pFS083 put2-/-* |
| *C. albicans*: CFG379 | This work | *NEUT5/neut5::FRT put1-::PUT1/ put1-* |
| *C. albicans*: CFG380 | This work | *NEUT5/neut5::FRT put1-::PUT1/ put1-* |
| *C. albicans*: CFG381 | This work | *NEUT5/neut5::FRT put2::PUT2/ put2-* |
| *C. albicans*: CFG382 | This work | *NEUT5/neut5::FRT put2::PUT2/ put2-* |
| ***C. albicans* BWP17-derived strains** | | |
| *C. albicans*: PLC096 | [13] | *ura3::imm434/ura3::imm434 iro1/iro1::imm434 his1::hisG/his1::hisG arg4/arg4 ADH1/adh1::P_ADH1_-yEmRFP-URA3* |
| *C. albicans*: CFG474 | This work | *ura3::imm434/ura3::imm434 iro1/iro1::imm434 his1::hisG/his1::hisG arg4/arg4 ADH1/adh1::P_ADH1_-yEmRFP-URA3 NEUT5/neut5::P_ENO1_-CC9-pFS092 put2- /-* |
| *C. albicans*: CFG479 | This work | *ura3::imm434/ura3::imm434 iro1/iro1::imm434 his1::hisG/his1::hisG arg4/arg4 ADH1/adh1::P_ADH1_-yEmRFP-URA3 NEUT5/neut5::FRT put2- /-* |
| ***C. glabrata strains*** | | |
| *C. glabrata*: ATCC2001/CBS138 | ATCC | Prototrophic wildtype |
| *C. glabrata*: GFS003 | This work | *put1Δ::FRT* |
| *C. glabrata*: GFS005 | This work | *put2Δ::FRT* |
| **Other yeast strains** | | |
| *C. albicans*: MAY7 | [14]; MA Collection | Clinical isolate; fluconazole resistant |
| *C. albicans*: PLC124 | POL Collection | From Karolinska Hospital (Solna) isolated from male patient with knee prosthesis |
| *C. utilis*: F608 | POL Collection | Prototrophic wildtype |
| *C. glabrata*: Peu927 | OB Collection | Clinical isolate |
| *C. tropicalis*: SM1541 | UR Collection | Clinical isolate |
| *C. tropicalis*: ATCC750 (1) | VDV Collection | Clinical isolate |
| *C. tropicalis*: ATCC750 (2) | UR Collection | Clinical isolate |
| *C. dubliniensis:* SMI718 | UR Collection | Clinical isolate |
| *C. dubliniensis*: Wü284 (1) | [15]; SR Collection | Clinical isolate |
| *C. dubliniensis*: Wü284 (2) | [15]; CU Collection | Clinical isolate |
| *C. parapsilosis*: ATCC22019 (1) | UR Collection | Clinical isolate |
| *C. parapsilosis*: ATCC22019 (2) | SR Collection | Clinical isolate |
| *C. parapsilosis*: ATCC22019 (3) | VDV Collection | Clinical isolate |
| *C. krusei*: ATCC6258 | SR Collection | Clinical isolate |
| *C. auris*: CFG552 | KK Collection | Clinical isolate |
| *C. lusitaniae*: DSM 70102 | KK Collection | Clinical isolate |
| *C. guilliermondii*: ATCC6260 | KK Collection | Clinical isolate |
| *S. cerevisiae*: S288c | Reference strain | Prototrophic haploid (1N) |
| *S. cerevisiae*: KRY001 | [16] | Σ1278b-derived diploid (2N) |
| *S. cerevisiae*: CFG638 | POL Collection | Clinical isolate from Karolinska Hospital (Huddinge), Sweden |
| *S. cerevisiae*: CFG639 | POL Collection | Clinical isolate from Karolinska Hospital (Huddinge), Sweden |
| *S. cerevisiae*: CFG640 | POL Collection | Clinical isolate from Karolinska Hospital (Huddinge), Sweden |
| *S. cerevisiae*: CFG641 | POL Collection | Clinical isolate from Karolinska Hospital (Huddinge), Sweden |
| *S. cerevisiae*: CBS6308 | [17] | Clinical isolate |
| *S. cerevisiae*: DBVPG1380 | [17] | Clinical isolate |
| *S. cerevisiae*: DBVPG3098 | [17] | Clinical isolate |
| *S. cerevisiae*: DBVPG4460 | [17] | Clinical isolate |
| *Cryptococcus neoformans* | KK Collection | Clinical isolate |
| **Oligonucleotides** | | |
| Oligonucleotides are described in **Table S2** | N/A | N/A |
| **Software and algorithms** | | |
| GraphPad Prism version 9 | GraphPad | GraphPad Software; <https://www.graphpad.com>; RRID: SCR_002798 |
| Huygens Deconvolution | Scientific Volume Imaging | <https://svi.nl/HomePage> |
| ImageJ software (Fiji v. 2.0.0) | NIH | <https://imagej.net/> |
| Image Lab 6.1.0 | Bio-Rad | <https://www.bio-rad.com> |
| LAS-X | Leica microsystems |  |
| OxyTrace+ Windows software | Hansatech Instruments |  |
| Serial Cloner 2.6 | Serial Cloner | <http://serialbasics.free.fr/Serial_Cloner.html> |
| SnapGene 6.1.2 | Dotmatics | <https://www.snapgene.com> |
| Zen Blue software | Zeiss |  |

**Reference**

1. Zhang C, Konopka JB. A photostable green fluorescent protein variant for analysis of protein localization in Candida albicans. Eukaryot Cell. 2010;9(1):224-6. doi: 10.1128/EC.00327-09. PubMed PMID: 19915075; PubMed Central PMCID: PMCPMC2805285.

2. Jenull S, Mair T, Tscherner M, Penninger P, Zwolanek F, Silao FS, et al. The histone chaperone HIR maintains chromatin states to control nitrogen assimilation and fungal virulence. Cell Rep. 2021;36(3):109406. Epub 2021/07/22. doi: 10.1016/j.celrep.2021.109406. PubMed PMID: 34289370; PubMed Central PMCID: PMCPMC8493472.

3. Silao FGS, Ward M, Ryman K, Wallstrom A, Brindefalk B, Udekwu K, et al. Mitochondrial proline catabolism activates Ras1/cAMP/PKA-induced filamentation in Candida albicans. PLoS Genet. 2019;15(2):e1007976. Epub 2019/02/12. doi: 10.1371/journal.pgen.1007976. PubMed PMID: 30742618.

4. Vyas VK, Barrasa, M.I., Fink, G.R. . A Candida albicans CRISPR system permits genetic engineering of essential genes and gene families. Sci Adv. 2015;1(3):1-6. doi: <http://dx.doi.org/10.1126/sciadv.1500248>.

5. Vyas VK, Bushkin GG, Bernstein DA, Getz MA, Sewastianik M, Barrasa MI, et al. New CRISPR Mutagenesis Strategies Reveal Variation in Repair Mechanisms among Fungi. mSphere. 2018;3(2). Epub 2018/04/27. doi: 10.1128/mSphere.00154-18. PubMed PMID: 29695624; PubMed Central PMCID: PMCPMC5917429.

6. Tscherner M, Stappler E, Hnisz D, Kuchler K. The histone acetyltransferase Hat1 facilitates DNA damage repair and morphogenesis in Candida albicans. Mol Microbiol. 2012;86(5):1197-214. Epub 20121017. doi: 10.1111/mmi.12051. PubMed PMID: 23075292.

7. Krauke Y, Sychrova H. Cnh1 Na(+) /H(+) antiporter and Ena1 Na(+) -ATPase play different roles in cation homeostasis and cell physiology of Candida glabrata. FEMS Yeast Res. 2011;11(1):29-41. Epub 20101013. doi: 10.1111/j.1567-1364.2010.00686.x. PubMed PMID: 20942808.

8. Clemmons AW, Lindsay SA, Wasserman SA. An effector Peptide family required for Drosophila toll-mediated immunity. PLoS Pathog. 2015;11(4):e1004876. Epub 2015/04/29. doi: 10.1371/journal.ppat.1004876. PubMed PMID: 25915418; PubMed Central PMCID: PMCPMC4411088.

9. Martinez P, Ljungdahl PO. An ER packaging chaperone determines the amino acid uptake capacity and virulence of Candida albicans. Mol Microbiol. 2004;51(2):371-84. doi: 10.1046/j.1365-2958.2003.03845.x. PubMed PMID: 14756779.

10. Silao FGS, Ryman K, Jiang T, Ward M, Hansmann N, Molenaar C, et al. Glutamate dehydrogenase (Gdh2)-dependent alkalization is dispensable for escape from macrophages and virulence of Candida albicans. PLoS Pathog. 2020;16(9):e1008328. Epub 2020/09/17. doi: 10.1371/journal.ppat.1008328. PubMed PMID: 32936835; PubMed Central PMCID: PMCPMC7521896.

11. Gillum AM, Tsay EY, Kirsch DR. Isolation of the Candida albicans gene for orotidine-5'-phosphate decarboxylase by complementation of S. cerevisiae ura3 and E. coli pyrF mutations. Mol Gen Genet. 1984;198(2):179-82. doi: 10.1007/BF00328721. PubMed PMID: 6394964.

12. Wartenberg A, Linde J, Martin R, Schreiner M, Horn F, Jacobsen ID, et al. Microevolution of Candida albicans in macrophages restores filamentation in a nonfilamentous mutant. PLoS Genet. 2014;10(12):e1004824. doi: 10.1371/journal.pgen.1004824. PubMed PMID: 25474009; PubMed Central PMCID: PMC4256171.

13. Keppler-Ross S, Noffz C, Dean N. A new purple fluorescent color marker for genetic studies in Saccharomyces cerevisiae and Candida albicans. Genetics. 2008;179(1):705-10. doi: 10.1534/genetics.108.087080. PubMed PMID: 18493083; PubMed Central PMCID: PMCPMC2390648.

14. Anderson MZ, Saha A, Haseeb A, Bennett RJ. A chromosome 4 trisomy contributes to increased fluconazole resistance in a clinical isolate of Candida albicans. Microbiology (Reading). 2017;163(6):856-65. Epub 2017/06/24. doi: 10.1099/mic.0.000478. PubMed PMID: 28640746; PubMed Central PMCID: PMCPMC5737213.

15. Morschhauser J, Ruhnke M, Michel S, Hacker J. Identification of CARE-2-negative Candida albicans isolates as Candida dubliniensis. Mycoses. 1999;42(1-2):29-32. doi: 10.1046/j.1439-0507.1999.00259.x. PubMed PMID: 10394844.

16. Davis MM, Alvarez FJ, Ryman K, Holm AA, Ljungdahl PO, Engstrom Y. Wild-type Drosophila melanogaster as a model host to analyze nitrogen source dependent virulence of Candida albicans. PloS one. 2011;6(11):e27434. doi: 10.1371/journal.pone.0027434. PubMed PMID: 22110651; PubMed Central PMCID: PMC3215725.

17. Peter J, De Chiara M, Friedrich A, Yue JX, Pflieger D, Bergstrom A, et al. Genome evolution across 1,011 Saccharomyces cerevisiae isolates. Nature. 2018;556(7701):339-44. Epub 20180411. doi: 10.1038/s41586-018-0030-5. PubMed PMID: 29643504; PubMed Central PMCID: PMCPMC6784862.
