# Supplementary material for: Proline catabolism is a key factor facilitating Candida albicans pathogenicity
Source: PLoS Pathog. 2023 Nov 2;19(11):e1011677. doi: 10.1371/journal.ppat.1011677 (PMC10621835; doi:10.1371/journal.ppat.1011677)
Supplement: S2 Table — (DOCX) [file ppat.1011677.s009.docx]

| **S2 Table. Oligonucleotides** | | | | | |
| --- | --- | --- | --- | --- | --- |
| - **No** | - **Primer Name** | | - **Sequence** | **Reference** | |
| - 1 | | - 161-PUT1-VerF | - CATCGGTTATTATTCTTCTTG | - [1] |  |
| - 2 | | - 162-PUT1-VerR | - GTTTAACCACTTCCAAATAATC | - [1] |  |
| - 3 | | - 244-PUT2-VerF | - CTAGCGGATTAACTATTCGC | - [1] |  |
| - 4 | | - 245-PUT2-VerR | - GGATAATGCGGCTGTAGCAG | - [1] |  |
| - 5 | | - 237-PUT3-VerF | - CGTGCATTACTTCATGTAATC | - [1] |  |
| - 6 | | - 238-PUT3-VerR | - GGACGAAGGTATTGTTTGAGG | - [1] |  |
| - 7 | | - 377-PUT1ver2 | - CGAGACAATCAATGGAAACC | - This work |  |
| - 8 | | - 378-PUT2ver2 | - CCACAGACTGAATGAAGAAG | - This work |  |
| - 9 | | - FS95 | - ggcatagctgaaacttcggc | - [1] |  |
| - 10 | | - 275-C-HATagPUT2F | - CTGGTAGTGGTAACATTTTATCCAGATTTGTTTCTATTAGAAACATTAAAGAAAACTTTTACGAATTGACTGATTTCAAATATCCATCCAATTATCAAaacatcttttacccatacgatg | - [1] |  |
| - 11 | | - 276-C-HATagPUT2R | - GGAAACAACATGAACACCTTATGTAAGAAAACCTCTTCTTAATATAAATATTTACATTCACACATTAACTATATAAAGTAATAACTAATCTCGTTTCTCgcaggttaacctggcttatcg | - [1] |  |
| - 12 | | - p110_FS244HAfor | - CTAGCGGATTAACTATTCGC | - [1] |  |
| - 13 | | - p111_FS340HArev | - cgtcatatggataggatcctg | - [1] |  |
| - 14 | | - 231PUT1Upstr | - CCACTCAATCAATGATCATCC | - This work |  |
| - 15 | | - FS321ADH1upstr | - GAGACCCAATGCAAAGCCAG | - This work |  |
| - 16 | | - JAV35-3'RFPtest | - GAATCTTGAGTAACAGTAAC | - This work |  |
| - 17 | | - 339VerGDH2HAF | - GTTCCATGTGGTGGTAGACC | - [2] |  |
| - 18 | | - 379GFPuniv | - gtccatctttacagtcctgtc | - [2] |  |
| - 19 | | - 277C-HATagPUT3F | - TTATATTTAATACTGCATCGCCTGATGTTGGCACTAGTGTCCATTCAGGGTATTCAAACTTGATAAACCATGAATTTCAAGATTTCATGGATCAATCTaacatcttttacccatacgatg | - This work |  |
| - 20 | | - 278C-HATagPUT3R | - GTTTTTGTAATATATTGTATTATATAGAAAATTTTATTACCATCACAGAATAAATGTACAGACATAAATATATATTTGCCTCACTCCCGCACAATCACTgcaggttaacctggcttatcg | - This work |  |
| - 21 | | - RT-PUT1Top | - GATTATATTCTAAACAATCTTTAAATACATTCAAAAAAGCTACATTTATA**TAA**ctcgag**TGA**TACTACTAC | - [1] |  |
| - 22 | | - RT-PUT1Bot | - GACAGTGACATTTTGTTTATTTGGGGTGTCATGGAAATATCTAAATTGAGTAGTAGTA**TCA**ctcgag**TTA**TATAAATGTAGC | - [1] |  |
| - 23 | | - RT-PUT2Top | - CAGACATACATATTCATTTATAATGTTAAGATCAACTACTCGTAATACATTA**TAA**A**TGA**ctcgagTACTAG | - [1] |  |
| - 24 | | - RT-PUT2Bot | - GTGTGACGAATGATACTTGATGAAACTTTAGTATATCTAGTActcgag**TCA**T**TTA**TAATGTATTAC | - [1] |  |
| - 25 | | - RT-PUT3Top | - CATTCCTTCATTTACTTATATATAATCCGATTCTTGTACAATGGATTCACAAGAGCCT**TAA**ctcgag**TGA**AGAAAATTGC | - [1] |  |
| - 26 | | - RT-PUT3Bot | - CAAGTGGAATGGTATCTGAATTAATTAATGCATTTGCAATTTTCT**TCA**ctcgag**TTA**AGGCTCTTG | - [1] |  |
| - 27 | | - 55-CgPUT1 | - TTCTTTCCTGCGTTATCCCCTGATTCTGTGGATAACCGTACCATGGGATCGCCTGCAGAGATGTTAG | - This work |  |
| - 28 | | - 53-CgPUT1 | - GAGGGGGGGCCCGGTACCCAATTCGCCCTATAGTGAGTCGCTTGTGATACTTGTGACGCTTG | - This work |  |
| - 29 | | - 35-CgPUT1 | - TAGTGAGGGTTAATTGCGCGCTTGGCGTAATCATGGTCATCTGATGTCAAGACTCTTTACGCA | - This work |  |
| - 30 | | - 33-CgPUT1 | - AACGCAGAAAATGAACCGGGGATGCGACGTGCAAGATTACCATAACTTCATCTATCACACGCTTGTG | - This work |  |
| - 31 | | - 5C-CgPUT1 | - CGAATAGTCCTCGAGAAACTGC | - This work |  |
| - 32 | | - 3C-CgPUT1 | - GACCTTGTCTTTGGCAGTGAAG | - This work |  |
| - 33 | | - LOG-PUT1_fo | - CTGATCGACAACTGCTCTAGAATC | - This work |  |
| - 34 | | - LOG-PUT1_re | - GACGTTGTCTGACATACCTAGC | - This work |  |
| - 35 | | - 55-CgPUT2 | - TTCTTTCCTGCGTTATCCCCTGATTCTGTGGATAACCGTACCATGGGCAGCACTCGAAACTCATAAC | - This work |  |
| - 36 | | - 53-CgPUT2 | - GAGGGGGGGCCCGGTACCCAATTCGCCCTATAGTGAGTCGCCAAGGAATAGATCAGAAACAGAC | - This work |  |
| - 37 | | - 35-CgPUT2 | - TAGTGAGGGTTAATTGCGCGCTTGGCGTAATCATGGTCATGATTTCAACTATCTACGCAGTGG | - This work |  |
| - 38 | | - 33-CgPUT2 | - AACGCAGAAAATGAACCGGGGATGCGACGTGCAAGATTACCATCTTACACGACTGAGTGAACATGG | - This work |  |
| - 39 | | - 5C-CgPUT2 | - GAAGCTACTACGGACACAACC | - This work |  |
| - 40 | | - 3C-CgPUT2 | - GACATTTGGGAAGCGGTATGC | - This work |  |
| - 41 | | - LOG-PUT2_fo | - CAGCTCTGATGGGTAACACTG | - This work |  |
| - 42 | | - LOG-PUT2_re | - AGCTTGTTGAAACTTTGCTCG | - This work |  |
| - 43 | | - hk2 | - CGTCAAGACTGTCAAGGAGGG | - This work |  |
| - 44 | | - hk3 | - CATCATCTGCCCAGATGCGAAG | - This work |  |
| - 45 | | - YEp_ic_fwd | - GTAATCTTGCACGTCGCATCC | - This work |  |
| - 46 | | - YEp_ic_rev | - TACGGTTATCCACAGAATCAGGG | - This work |  |
| - 47 | | - SATflipp_fwd | - CGACTCACTATAGGGCGAATTGG | - This work |  |
| - 48 | | - SATflipp_rev | - TGACCATGATTACGCCAAGC | - This work |  |

**Reference**

1. Silao FGS, Ward M, Ryman K, Wallstrom A, Brindefalk B, Udekwu K, et al. Mitochondrial proline catabolism activates Ras1/cAMP/PKA-induced filamentation in Candida albicans. PLoS Genet. 2019;15(2):e1007976. Epub 2019/02/12. doi: 10.1371/journal.pgen.1007976. PubMed PMID: 30742618.

2. Silao FGS, Ryman K, Jiang T, Ward M, Hansmann N, Molenaar C, et al. Glutamate dehydrogenase (Gdh2)-dependent alkalization is dispensable for escape from macrophages and virulence of Candida albicans. PLoS Pathog. 2020;16(9):e1008328. Epub 2020/09/17. doi: 10.1371/journal.ppat.1008328. PubMed PMID: 32936835; PubMed Central PMCID: PMCPMC7521896.
