# Supplementary material for: Proline catabolism is a key factor facilitating Candida albicans pathogenicity
Source: PLoS Pathog. 2023 Nov 2;19(11):e1011677. doi: 10.1371/journal.ppat.1011677 (PMC10621835; doi:10.1371/journal.ppat.1011677)
Supplement: Attachment — Submitted filename: 133524_1_rebuttal_2730809_rxlp3s.docx [file ppat.1011677.s014.docx]

Response to the Reviewers

The review process has provided helpful suggestions as to improve and clarify several aspects of our manuscript. The experimental work described in the paper is complex and as Reviewer 1 correctly points out, represents **“***an important and timely manuscript, which significantly contributes to the understanding of how proline metabolism intersects with yeast fitness in the context of infections*.” However, we apparently were not clear enough in our writing, which apparently contributed to reviewers 2 and 3 misjudging the significance of our work. We have addressed the concerns of all three reviewers and have worked to streamline the presentation to highlight the novelty and significance of our data, and have adjusted the title to “***Proline catabolism is a key factor facilitating Candida albicans pathogenicity***.”

Summary of our findings:

To survive in the host environment, fungal pathogens must propagate under similar physiological conditions as host cells. Proline is abundant in the human body, typically fixed in extracellular matrix proteins (e.g., collagen), and is released in significant quantity during various pathological states. In this manuscript we document the importance of proline catabolism in supporting the energy demands of virulent fungal growth. We have applied multiple experimental approaches to obtain novel insights that are of broad relevance and, in contrast to what is implied in comments by reviewer 3, this work represents a substantial and significant increase in understanding. Our manuscript is summarized in the following five points:

1) We tested and found that many *Candida* spp. known to cause mycoses in humans, including the recently described and multidrug resistant *C. auris*, have the capacity to catabolize proline as sole source of energy (**Fig. 1**). **Although other non-pathogenic fungi have been shown to use proline (as pointed out by Reviewer 2),** **this metabolic attribute has not been previously tested in members of the pathogenic *Candida* spp. complex**.
2) Using *C. albicans* as a paradigm species of the complex, we defined regulatory mechanisms controlling proline utilization and confirmed a tight coupling to mitochondrial function (**Fig. 2)**. We report key regulatory differences exist between *C. albicans* and *S. cerevisiae* that presumably reflect the microenvironments in which these fungi evolved. *S. cerevisiae* evolved to deal with high sugar environments and is widely found in nature. By contrast, *C. albicans* and other members of the *Candida* spp. complex have evolved growing in symbiosis with human hosts and are rarely found outside of mammalian hosts. **The results are novel and clear up many misconceptions stemming from using *S. cerevisiae* as the paradigm fungal organism.**
3) We report that under respiratory conditions, proline becomes toxic to cells that lack the ability to catabolize it. We traced the source of toxicity to the inhibition of mitochondrial respiration. The fact that we observe similar levels of inhibition, i.e., reduced O_2_ consumption, in both *put1* and *put2* mutants is striking, and indicates proline itself interferes with respiration. The proline hypersensitivity exhibited by *put2* mutants correlates with increased levels of P5C, a known toxic intermediate that does not accumulate in *put1* mutants, and elevated levels of ROS. However, contrary to current understanding, we found that *put2* mutant-dependent toxicity is primarily linked to inhibition of mitochondrial respiration and that ROS formation is a secondary effect. **These totally unexpected findings** (**Fig. 3) may open up novel opportunities for antifungal treatments.**
4) *C. albicans* strains lacking the capacity to catabolize proline exhibit impaired virulence characteristics. It is important to note that the *put* mutations do not target proteins with diverse and overlapping functions, such as transcription factors or signaling proteins, so the phenotypes we document, particularly for *put1* and *put2*, are clearly due to direct effects. We have developed and applied a novel collagen transwell assay and also used a reconstituted human skin model to monitor invasive growth (**Fig. 4**). The capacity for virulent growth was assessed using both fly and mouse models of systemic infections, and we also examined survival during co-culture with whole human blood and human neutrophils (**Fig. 5**). **The results from each of the diverse assays were consistent in showing that proline catabolism constributes significantly to the ability of *C. albicans* to proliferate in host envrionments and grow virulently. This has not been shown before.**
5) We have successfully used intravital 2-photon microscopy to visualize early events during the infection of *C. albicans* in the kidneys of a living murine host (**Fig. 6**). We observed the formation of hyphae in real-time, directly deep in the tissue, and discovered that this is dependent on the ability of fungal cells to catabolize proline. The inability of a *put2-/-* mutant to undergo morphogenesis within the comparable timeframe as that of the wildtype provides a readout for proline accessibility. Here we thank Reviewer 3 for suggesting that we place our results in context of the beautiful work by Lionakis et al. (2013) J Clin Invest, which demonstrated the special role of host macrophages in kidney infections. Our previous work, Silao et al. (2019) PLoS Genetics and Silao et al. (2020) PLoS Pathogens, has shown that phagocytized *C. albicans* cells respond to proline present in the phagosome to initiate hyphal growth, providing the means to escape macrophages. Importantly, hyphal growth and escape does not require alkalization, as *C. albicans* strains lacking *GDH2* are able to undergo morphogenesis and escape macrophages. Consequently, the host response as documented by Lionakis et al. dovetails perfectly with the pathogen requirement of *C. albicans*. **The knowledge gained and the technical advances represented by the successful application of intravital microscopy opens up new possibilities to address fundamental questions regarding virulent fungal growth with broad biological implications.** **This is the first report of intravital 2-photon microscopy of a fungal kidney infection.**

Together our findings demonstrate that a proline catabolic network tailored and tuned to the mammalian host environment is an important characteristic shared by multiple human fungal pathogens, and our results highlight the significance of proline as an excellent source of cellular energy. Intriguingly, our results display similarity to observations that proline metabolism can support the progression of various invasive diseases, including cancer during nutrient stress (Phang, J. M. (2019) Proline Metabolism in Cell Regulation and Cancer Biology: Recent Advances and Hypotheses. *Antioxid Redox Signal* **30**, 635-649). Consistently, fungal cells have recently been found associated with human cancers (Narunsky-Haziza, L. *et al.* (2022) Pan-cancer analyses reveal cancer-type-specific fungal ecologies and bacteriome interactions. *Cell* **185**, 3789-3806 e3717), clearly suggesting that fungi and other constituents of the microbiome can benefit through spatial association with cancer cells and accompanying macrophages at sites of metastatic growth, perhaps the consequence of cross-feeding host-derived nutrients, i.e., proline derived from the degradation of the extracellular matrix.

**The data documented in this manuscript are entirely novel and we find it difficult to accept the notion that the results presented are incremental.**

Point-by-Point Response (author’s replies in blue)

------------------------------------------------------------------------------ 
Reviewer #1 (Evidence, reproducibility and clarity (Required)):

Summary: Silao et al make the intriguing observation that yeasts that are generally considered less pathogenic are unable to catabolize proline than Candida albicans. They then, in Candida albicans, construct mutants defective for the two key enzymes (Put1, Put2) required to convert proline to glutamate, which they show to be essential for proline utilization as an energy (carbon) and nitrogen source. The authors proceed to untangle the regulatory aspects of proline degradation, including the respective cellular localization of its key enzymes. They then make the important discovery that strains lacking either Put1 or Put2 suffer from a proline-dependent growth defect, which they attribute to resulting defects in mitochondrial metabolism.

The manuscript then goes on to analyze a broad range of infection models including: reconstituted human epithelial skin model, Drosophila, mouse systemic infections, organ colonization in these mice (kidney, spleen, brain, liver and histochemistry of the kidneys) as well as survival when incubated with cultured human neutrophils. Finally, they use yeast cells constitutively expressing yEmRFP (so that yeasts can be distinguished from other host cells) and coated with FITC before incubation with the host cells (which coats the wall of the original cells, but does not spread to progeny) and they go on to perform an impressive set of analyses of C. albicans growth within mouse kidneys both in vivo and ex vivo, exploiting an implanted window together with intravital imaging with a two photon microscope at different time points. The system is impressive and visualizes tissue invasion by hyphal cells beautifully. Finally, they compare the intra vital images from WT and put2-/- cells and show that, as in vitro, put2-/- cells do not form filaments and do not show extensive invasion of the kidney tissue. While the in vivo aspect of the study includes many different models, it finds defects in virulence for different subsets of put mutants and the relative importance of filamentation vs proline utilization for virulence is not conclusively resolved.

Overall, this is an important and timely manuscript, which significantly contributes to the understanding of how proline metabolism intersects with yeast fitness in the context of infections. However, there are several major concerns regarding some of the conclusions drawn from the study. In addition, some general recommendations that would improve the manuscript are provided.

Specifically, the manuscript provides a very detailed description of experiments and observations. However, in several parts it is difficult to follow and the reader needs more guidance about the logic involved in reaching conclusion. Specifically, several aspects of the paper are written for experts in Candida (yeast) metabolism. Here, explaining the rationale for some of the experiments, and providing more background information that is not obvious to a non-expert, is required.

In particular, writing a clear and measured summary sentence at the end of each paragraph and a conclusion paragraph that summarizes key findings in simple terms would help make the manuscript more digestible for readers.

In addition, the impressive microscopy and broad range of in vivo experiments is comprehensive but only adds incremental information relevant to proline metabolism-that filamentous growth in vivo and virulence is reduced in cells carrying some mutations in one or more put genes. However, this broad sweep of model systems and the development of the in vivo imagining system might have more impact in a separate paper focused on the real-time in vivo visualization of kidney invasion.

We thank Reviewer 1 for the extensive list of comments and have endeavored to adjust the manuscript to address all of the major and minor concerns. It is evident that Reviewer 1 clearly understood the significance of the work and we appreciate that the comments are presented in a positive manner intended to improve our manuscript.

Major comments:

1. The main finding that impressed this reviewer is that "removing the ability to catabolize proline, in an organism that evolved to catabolize it, leads to (growth) defects". This point could be better highlighted throughout the manuscript. Thanks for the comment. We will adjust the text to reflect this suggestion.

2. The authors show that deletion strains for proline metabolism have defects that are important for in vivo pathogenicity. This is an important finding. However, as the manuscript reads now, it suggests that the main findings are that the ability to use proline in the respective host niche is key. Mechanistically, the manuscript revolves primarily around defects that arise when deleting PUT1 and/or PUT2 (i.e., an "unknown" toxicity of proline in the case of put1-/- (or put1-/- put2-/-) and the additional P5C-dependent toxicity for put2-/- mutants; see below). Yes, the reviewer is correct in that we believe that proline catabolism is necessary to initiate and power hyphal growth, which is coupled to virulence. We have previously shown that upon phagocytosis by macrophages, the expression of Put1, Put2 and even Gdh2 are induced in phagocytized *C. albicans* cells, which is consistent with the analysis shown in Fig. 2D and Fig. S2B. Consequently, proline, or an amino acid that is metabolized via the proline catabolic pathway, must be present in the phagosomal compartment. However, as we now report, proline inhibits growth of cells lacking the capacity to catabolize it. Although we cannot differentiate the cause of reduced virulence in *put* mutants, i.e., the lack of energy due to the inability to catabolize proline vs proline toxicity, proline catabolism is clearly important and a robust indicator of virulence. As point 1, we have adjusted the text to make this clearer.

3. In order to claim that catabolizing prolines promotes pathogenicity (as opposed to the alternative hypothesis that the inability to catabolize proline leads to the observed defects), additional experiments would be required. For example, the put mutants would need to be compared with mutants that significantly reduce/impair proline uptake, such as the referenced gnp2 mutant (Garbe et al 2022). While the finding that less pathogenic yeast species are unable to catabolize proline is both intriguing and important, it also remains as is presented as a loose, non-quantitative correlation that only tangentially address the question of whether "proline catabolism is key for pathogenicity". We have in fact already shown that proline uptake is required to induce filamentation (Martínez and Ljungdahl 2003, Fig. 6). The main point of our current work, which we believe is important and of general interest, is that C. albicans is adapted to use proline as sole energy source, which reflects the environment (humans) in which it evolved. See the response to point 2. Interestingly, the differences in the expression levels of Put1 (off in the absence of proline, induced robustly by proline) and Put2 (low level of constitutive expression, induced robustly by proline) suggest that cells are primed to decrease the likelihood of becoming inhibited by P5C, i.e., the constitutive expression of Put2 is able to ameliorate the potential toxicity of P5C. Regardless, the finding that *put1* and *put2* mutants exhibit significantly reduced virulence in two host models provides clear support for proline catabolism being key for *C. albicans* pathogenicity.

4. 238 onwards: The conclusion that "the primary growth inhibitory effect of proline is linked to catabolic intermediates formed by Put1 and that are metabolized further by Put2"does not appear to be fully supported by the evidence. Addition of proline to put1 mutants already reduced OD600 by ~50% (Figure 2); and is further reduced to ~10% when put2 is deleted. This implies that there are two inhibitory effects of proline, not one primary one. At the least, this option should be discussed, including why deletion of PUT1 leads to proline toxicity. The latter is not clear-is it that too much proline accumulates in the cell and this accumulation is toxic? If this is the case, the effect would be expected to be proline concentration dependent. Performing a relatively simple experiment as performed for the put2 mutant (Fig. 3 / S3F) may clarify this issue. Particularly, if the experiment would be coupled with intracellular quantification of proline. Precisely! Proline toxicity is evident even in *put1* mutants, clearly suggesting that proline, without being further catabolized, exerts a growth inhibitory effect (Fig. 3A). We traced this inhibitory effect to decreased mitochondrial respiration (Fig. 3E). There are two parameters to consider regarding the inhibitory effects of proline in *put2* mutants. First, the presence of proline induces the expression of Put1 independent of Put2 (Fig. S2C), consequently, the levels of the toxic intermediate P5C increases (Fig. 3B). P5C has previously been postulated to inhibit mitochondrial respiration, which is well-aligned with our analysis (Fig. 3E; see response Point 5). We initially tested whether a proline-P5C cycle, suggested by work in mammalian cells, would play a role in proline-mediated toxicity; however, increasing cytoplasmic pools of proline by supplying high levels of glutamate (which according to work in mammalian cells should efficiently convert to cytoplasmic proline) did not occur; we did not see glutamate-enhanced Put1 expression (Fig. 2D, S2A, S2B). We agree with the reviewer with respect to the suggested experiment, and have monitored growth of *put1* in media with different proline concentrations. The results are incorporated in the revised Fig. 3.

5. The caption "P5C mediates a respiratory block" is misleading, as the evidence is not that compelling: Although P5C increases in put2, but not in put1 mutants, and given that both single mutants experience a proline-dependent respiratory defect (Fig. 3E), the results suggest a more complex relationship. Previous work using pure P5C (Ref. 36; Nishimura et al) showed that it targets respiration, hence the caption “respiratory block” in the header. In mammals, PRODH (Put1) physically interacts with mitochondrial respiratory complex II in the inner mitochondrial membrane (line 89-90), while P5CDH (Put2) is in the matrix. The *put1* mutation might affect basal activity of the respiratory chain resulting in lowered respiration, which may compound when proline accumulates in the mitochondria. The inhibitory mechanism remains unknown, and in going forward we have begun characterizing various GFP-tagged respiratory complex components in *put1* mutants and in strains co-expressing Put1-RFP (for interaction studies). The results are out of the scope of this current work.

6. The virulence assays and in vivo experiments do not present a unifying view: in Drosophila put2∆∆ is less virulent than put1∆∆, which appears similar to put3∆∆. Given that put2 mutants grow slowly, likely because of P5C inhibition, this seems logical. However, in mice, put3∆∆ remains highly virulent while put1∆∆ and put2∆∆ results for survival are mixed. Furthermore, in 4 mouse organs, put1∆∆ and put2∆∆ are not significantly different from one another but are different from wt, while put3∆∆ has no significant reduction in CFU. Kidney histology shows very little invasion by put1 and put2 and more by put3, but visually put3 appears to invade much less than the WT, and the human neutrophil experiment shows effects of put2 or put3 but not put1. This leaves the reader rather confused. It may be worth discussing the reasons for different results in different models. Is the availability of proline in each of the organisms and organs similar? We thank the reviewer for these thoughtful observations, however, we note that all of the diverse assay systems employed provide a clear and consistent indication that the inability to completely catabolize proline significantly reduces virulence. This is well-aligned with our previous data regarding the need for proline catabolism to escape macrophages (Silao et al, 2019). The requirement for Put3 may not be very strict since the Put enzymes are still expressed in the absence of Put3 (Fig. 2D/S2A/S2B), indicating the activity of additional regulatory factors; hence, this may explain why the *put3* strain behaves like wildtype in the murine model (Fig. 5B). The dispensability of Put3 in the murine model could be due to a lower neutrophil count and that murine neutrophils exhibit a lower affinity for fungal cells as compared to human blood (Machata et al., 2020, Front Immunol). The more pronounced requirement of Put3 to survive in whole human blood and when co-cultured with human neutrophils could indeed be linked to the need to rapidly derepress *PUT1/PUT2* (and even other target genes) as suggested by the global RNASeq analysis that shows that proline catabolism is a core response of *C. albicans* during neutrophil interaction (Niemiec MJ et al., 2017, BMC Genomics). In Drosophila, a well-established model to study innate immunity, the presence of hemocytes that fulfill the equivalent functions of neutrophils and macrophages could explain the increased requirement for Put3. In summary, although it is impossible to know the precise mechanistic basis underlying the observed differences, we believe it unreasonable to expect that all mutations behave identically in each virulence model. In fact, differences considered trivial such as the use of mouse background can have profound effects on virulence. Presumably the differences we report are due to the specific nutrient composition (proline and metabolites feeding into the proline catabolic network) and physical parameters intrinsic to each model. For instance, Lionakis et al. (2013) suggested that filamentation occurs faster in the kidney compared to other organs, such as the liver/spleen, indicating the presence of kidney-specific cues that drive infections of this organ.

7. The ex vivo and in vivo analysis of the dynamics of C. albicans growth in the host is visually impressive, but it distracts from the focus of the paper and the metabolic findings. Showing that put mutant cells do not form filaments in vivo (as in vitro) does not add much conceptually to the paper. Furthermore, this lovely advance in in vivo visualization is lost at the end of this paper and the authors should consider whether it might fit better in manuscript that could really highlight the in vivo visualization approach. We appreciate this comment. Indeed, our lab is at an advanced stage of completing a manuscript focused on the use of intravital and clearing microscopy to follow the onset of an upper urinary tract infection (UTI) in a murine candidemia model. However, our ability to visualize in 3D the onset of an infection in a living host is not a trivial achievement and we were impressed that it provided a clear answer as to whether a single *C. albicans* cell can initiate an infection and undergo morphogenesis leading to hyphal growth. Furthermore, we tested a *put2* strain, the growth of which is highly sensitive to the presence of proline, and found that it did not exhibit filamentous growth. This clearly shows that cells colonizing the kidney are exposed to an environment that requires a functional proline catabolic network to exhibit filamentous growth, a characteristic of renal infections. Our results are consistent with the kidney being a metabolic hub for arginine/proline biosynthesis, which likely increases the levels of these amino acids in this organ.

8. The discussion of cells stained with FITC and expressing yEmRFP does not clearly point out that the FITC is only an indicator for those cells that were used to innoculate the tissue and that finding cells without FITC indicates that they are mitotic progeny, indicating that they have been dividing. The authors clearly understand this, but a naive reader may miss this important point if it is not stated explicitly. We have adjusted the text to explicitly clarify this.

Minor comments:

1. Throughout: what is the distinction between utilization of proline for C or for energy? These terms seem to be used interchangeably. *C. albicans* is heterotroph that can use proline to generate biomass (gluconeogenesis, etc) and its catabolism generates sufficient amounts of ATP to power growth. Thus, when proline is used as sole carbon source, it can also serves as the sole energy source. In the text, we have tried to be consistent using “carbon source” when discussing proline as a component of growth media, and “energy source” when discussing proline catabolism.

2. Introducing the schematic in Fig. 2A at the beginning of Figure 1, would help explain proline catabolism before delving into the growth experiments that rely upon this framework. This should include an explanation, for readers less familiar with the metabolic issues, of the main limitations to catabolizing proline, and the key issues for being able to use proline for nitrogen, carbon, and energy (potentially indicated in the overview figure, e.g. pointing towards gluconeogenesis etc.). We have considered the reviewers suggestion, however, we believe that the placement of the schematic in Fig 2 is appropriate as is, and where it will hopefully enable readers to more readily grasp the strain construction and experiments documented in Fig.2.

3. Saccharomyces can only grow on proline as a nitrogen source, but not as energy/carbon source. Could the authors briefly mention or discuss why this is the case? This is not clearly apparent after reading the manuscript and it leaves the reader confused and trying to understand if the fact that proline is required for carbon utilization is a new finding of this paper or was already known. Do the authors think this is tied to the presence of complex 1 components in C. albicans that are not found in S. cerevisiae. Is this consistent for the pathogenic, but not the non-pathogenic yeasts analyzed in figure 1? We have adjusted the text to clarify our thoughts regarding this. Indeed, we do believe that a major reason for the ability of *C. albicans* to efficiently grow using proline as a sole energy source is the presence of Complex I. However, *C. glabrata* appears to be able to grow well using proline as sole energy source despite apparently lacking Complex I. Consequently, alternative NADH dehydrogenases exist in *C. glabrata*, but how this is coupled to energy metabolism will require additional work that is out of the scope of the present work.

4. 100: While Gdh2 is apparently an important enzyme for generating ammonium, why is it not necessary for macrophage escape and virulence as shown in reference 18? A recent paper from Garbe et al (ref 12) suggests that Gnp2 is the major proline permease in C. albicans and what is known, and not known, about proline uptake would be good to mention, given that PUT gene functions require that proline enters the cells. We have recently shown that ammonia generation by Gdh2 is dispensable for macrophage escape and documented that phagosome alkalinization is not a requisite for the induction of hyphal growth (Silao et al. 2020). We have referred to the work of Garbe et al., which is consistent with our previous work (Martinéz and Ljungdahl, 2004) where we reported that proline-dependent filamentation is dependent on Csh3. Csh3 is an ER membrane-localized chaperone responsible for catalyzing the proper folding of amino acid permeases, in *csh3* null mutant strains, amino acid permeases accumulate in the ER as non-functional unfolded aggregates. Consistently, we have tested and found that proline-induced Put2-GFP expression is dependent on Csh3 (unpublished), clearly establishing that the regulatory effects of proline are dependent on its uptake. We have not generated a *gnp2*-/- strain, but suspect that we could find growth conditions where such a mutant would be refractory to proline induction. We have adjusted the text to include this information.

5. 116: Is the "low sugar environment of the host" referring to a specific niche, such as the GI tract, or human blood? Compared to most natural environments, glucose is abundant in the host, e.g., at ~5 mM, it is the most abundant metabolite in blood, and similarly, in the GI tract, levels can go beyond 50 mM glucose (see e.g. PMIDs 34371983, 21359215). Or is this comment indicating that the in vivo sugar concentration is lower than that in common lab growth media? Please spell out the niche/concentration for clarification - and compare that to other niches that are considered "high sugar environments". We have adjusted the text to clarify our statement. The natural environment *of C. albicans* is the human host. Virulent infections are not within the GI with high sugar content, but rather result when *C. albicans* cells successfully cross into the blood with a relatively low glucose (5 mM), which importantly is a level that does not effectively repress mitochondrial function. A major point of our recent work is that laboratory experiments with *C. albicans* growing on YPD or SD with 2% glucose (111 mM) examine growth of cells with repressed mitochondrial functions.

6. 123: "proline as sole energy source" - suggest "is the source of carbon, nitrogen, and energy" The text is adjusted (see response to Minor Point 1).

7. 142: it is worth noting to readers that C. neoformans is a basidiomycete and thus VERY distant from the other yeasts studied here-it is in a different major phylum of fungi. Again, thanks for this suggestion, the text is adjusted. We included *C. neoformans* since the role of proline catabolism has been characterized and linked to its pathogenicity (reviewed in Christgen and Becker, 2018, Antioxi Redox Signal, Ref. 1).

8. 143: Here it is implied that put1 and put2 mutant strains do not grow on SPD, but this is not stated explicitly. The *put1* and *put2* mutants are unable to grow in/on all media containing proline as sole nitrogen source. The phenotype is very tight that we were able to exploit this as a selection phenotype for reconstitution (Fig. 1A). We have adjusted the text to make this clear.

9. 151: The abbreviation SPG is not explained in main text. This was explained in the methods (1% glycerol as primary carbon source). As suggested, we have defined SPG in the main text.

10. Paragraph 156 onwards: this section is particularly hard to read and very dense. Also, it is difficult to understand the significance of these experiments for the overall findings of the paper. Please at least provide a small conclusion / summary at the end of the paragraph that puts the findings into perspective. We have adjusted text to make it more accessible.

11. Figure 2 C: simplifying the scheme (e.g. lots of redundant information, P2 and Mito - just give it one name) would help. This figure may be better in the supplementary material. The schematic of our subcellular fractionation study uses standard designations routinely used by the cell biology community. We believe that its inclusion will help readers judge the how we mapped the intracellular localization of the reporter proteins, which is essential to understand the proline catabolic network.

12. Figure 2B: It is not directly apparent from the micrographs that Put1-RFP localisation is mitochondrial. Co-localisation of the RFP with a mitochondrial dye (e.g., mitotracker) or something similar is required to validate it. We have previously reported that Put2 is a bona fide mitochondrial protein (by confocal microscopy, subcellular fraction, and co-localization with Mitotracker (Far Red) (Silao et al., Ref 17). The fact that the Put1-RFP associated fluorescence exhibits a distinct mitochondrial signature, is spatially exclusive and exhibits no overlap with the cytosolic pattern of Gdh2-GFP, co-fractionates with Put2-HA and the mitochondrial marker Atp1, should suffice to confirm that Put1-RFP is a mitochondrial localized protein.

13. Throughout the manuscript (figure legends): Suggest using "mean" instead of "Ave." We have adjusted the legends.

14. 175: According to the 'Yeasttract' and 'Pathoyeasttract' databases, Put1 regulates at least 36 and 22 genes, in S. cerev. and C. alb., respectively (based on DNA binding and/or regulatory changes). The only gene in common between these two lists of genes is PUT1. Thus, it is quite likely that Put3 regulates many other processes that explain its function and that its major function may not be only to regulate Put1. We assume that the reviewer is referring to Put3 (instead of Put1). Yes, Tebung et al. (2017) suggested that Put3 also regulates other genes. However, their data show that *C. albicans* *put3* mutant was unable to grow in medium (YCB+Pro) compared to SPD (2% glucose as carbon source) where proline is used merely as a nitrogen source (Tebung et al., Fig. 3A). Our data in Fig. 1C shows that a *put3* null strain exhibits residual growth on SPD, which aligns well with the expressed levels of PUT enzymes (Fig. 2D). Our conclusion is that despite being essential for rapid proline-dependent derepression of proline catabolic genes, Put3 is not the only transcription factor operating at the promoters of the *PUT* genes.

15. 175: Is it clear whether the Put3-independent mechanisms are positive or negative with respect to Put1? We have accumulated evidence that an additional transcription factor positively regulates *PUT1* expression and have a manuscript in preparation to describe this factors. The manuscript will focus on the Put3-independent regulation of *PUT1*, *PUT2*, and *GDH2* expression.

16. 218: Suggestion: "growth was indistinguishable".Unless growth curves or growth rates are provided and if one time-point data are the basis for this point, than "rates" is not a relevant term. The reviewer is correct; we will adjust the text accordingly. We have performed growth assays in a multi-well microplate format (Bioscreen) and found that the growth rates are not statistically different between WT, *put1*, *put2*, and *put1* put2 strains in the presence and absence of proline in SD with 2% glucose. This is consistent with glucose repression of mitochondrial function, i.e., proline toxicity depends on derepression of mitochondrial function.

17. 256 onwards: did the authors test if the ROS scavenging effectively reduced ROS? i.e. does the luminol-HRP assay yield less ROS in +proline +scavenger treatment? This is necessary to effectively conclude that the growth inhibitory effect of proline is due to blocking respiration. Indeed, we used NAC as a control in the luminol-HRP system and we saw reduction in ROS formation. In fact, this is the underlying reason why we used high levels of NAC for growth rescue (in Fig. 3D). We include the control data as Fig S3F – see graph.

18. The Figure captions are extremely lengthy and detailed, making it cumbersome to find the relevant information. Suggest moving some of the information, such as additional experimental details, into the methods section. We have streamlined the figure legends.

19. 277-301: Phloxine is not exclusively a live/dead cell indicator-it is an indicator of metabolic activity. In Scerev. and Calb. it also indicates slower growth, opaque growth, and it has been used as an indicator of aneuploidy in C. glabrata (<https://journals.asm.org/doi/10.1128/msphere.00260-22>) and of diploids vs haploids in S. pombe. The colonies illustrated aer made up of many live cells, and thus the section "Defective proline utilization is linked to cell death" needs to be presented more carefully. In addition, it appears that this section shifts from using defined medium to using rich medium and 37C instead of 30C. Why was this shift necessary? The reviewer is correct that phloxine (PXB) has been used to identify opaque growth (EFG1-dependent). However, the fact that the accumulation of PXB in the *put* mutants is evident in both SC5314 and *cph1 efg1* backgrounds (Fig. 3G and Fig. S4C) suggests that we are not assaying opaque switching. We mention that we have observed an increase in the number of PI+ cells in *put* mutants under similar conditions, but as we pointed out, we were unable to reliably quantitate this by FACS due to the clumping of *put* mutants. Zheng et al 2022, the paper cited by the reviewer, used PXB to assess the ploidy of *C. glabrata* strains, but their assay was developed using 5 μg/ml PXB, half of the concentration we used. The homogenous accumulation of PXB as the macrocolonies grow (Fig. 3G), suggests that the accumulation is not a consequence of spontaneously occurring ploidy variations. Thus, we believe that the accumulation of PXB does indeed reflect enhanced cell death. The point here is to trace the consequences of proline toxicity and to test the dependency on mitochondrial function. We used complex media, which contains multiple nitrogen sources (amino acids, peptides), to specifically highlight the contribution of proline catabolism in the fitness of *C. albicans*. The *put1*, *put2* and *put1 put2* mutants grow normally on YPD+PXB (30 ^o^C) without accumulating the dye; we only observed visible PXB uptake in *put2* after 2-3 days in mature macrocolonies. We attribute the gradual increase in PXB accumulation to be a consequence of glucose becoming limiting, derepressing mitochondrial functions, a requisite for proline toxicity. Consistently, the accumulation is more evident in cells grown on non-fermentable C-sources (Fig. 3G and Fig S4C).

20. 295-301: Related to the point above, these results are hard to interpret due to the switch from defined medium in all prior experiments to rich growth medium here. Also, it is not clear why a 48h old YPD culture was chosen to show that the degree of PI staining correlates with mitochondrial activity - is this due to the culture age? It would be more clear to image cells grown on glucose vs. glycerol/lactate, or under repressive / de-repressive glucose concentrations (e.g., as shown in Fig. S4C where a PI+ difference is apparent for 0.2% glucose vs. 2% glucose at 30 ^o^C). See response to Point 19 for our rationale to switch to rich medium. We have adjusted the text to enhance its readability. In liquid YPD, all strains grow, however, we noticed that the *put* mutants tend to flocculate (sign of stress in yeast) when cells enter stationary phase; see erratic OD readings below, particularly evident in the *put1* mutant (red) in YPL (included here for the reviewer, but not included in the manuscript). At 48h, the cultures become dense and cells experience glucose limitation, derepress mitochondrial functions and exhibit maximal flocculation (Fig. S4D). In *put* mutants, the derepression of mitochondrial function results in proline sensitivity. We tested the notion that this would also increase cell death, which it does, see Fig. S4E.

21. 313-14: The statement 'the invasion process was dependent on the ability of cells to catabolize proline' doesn't take into account that put mutant cells are defective in filamentous growth irrespective of their utilization of proline...and like the efg1 cph1 double mutant. Proline-induced filamentous growth is dependent on the catabolism of proline, which activates Efg1 and consequently the hyphal growth program. In Fig. 4A we show that *put* mutants grown on Spider media, initiate filamentation (as evidence by wrinkled colonies) but do not grow invasively (no halo). In Fig. 4B we developed and used a novel invasion assay to assess growth through a collagen plug. Similar to the control *cph1 efg1* mutant, the *put* mutants exhibit drastically reduced capacity to penetrate through the plug, and reach the D10 media in the transwell (D10 = DMEM with 10% FBS). However, it is important to note that although these results are linked to two distinct processes - the filamentation defect of *cph1 efg1* is due to the inability respond to multiple filamentation cues (e.g., CO_2_, 10% FBS, etc.), whereas the filamentation defect of the *put* mutants is linked to the inability to catabolize proline and to its toxicity. Clearly, the WT strain relies on proline catabolism, coming from one or three possible sources of proline (see response to Reviewer 3): 1) DMEM/F-12 medium used in the PureCol EZ Gel; 2) diffusion of nutrients up through the collagen from the recovery medium DMEM supplemented with 10% FBS; and 3) the proteolytic breakdown of collagen. Also, in contrast to the *put* mutants, WT cells are refractory to inhibition by proline.

22. 316-327: The results of the experiment described can only be interpreted as an effect of proline catabolism if the three strains (efg1 cph1; put1; put2) have similar growth rates as yeast cells in vitro. Why weren't the cells competed directly (efg1 cph1 vs put cells)? We believe that the relevant comparisons are to WT. We recovered cells from the top of the collagen (see Fig. 4B inset) to monitor their ability to survive and grow on top of the collagen. We found that the ability to catabolize proline enables WT and *cph1 efg1* cells to grow equally well (recovered similar ratio as starting input). This was not the case with the *put* mutants, they did not grow as well and almost 100% of the cells recovered were WT.

23. Fig 6: The logical order of the experiments, and in the text, is: 1) 4 h window, 2) 26 h window and then 3) ex vivo. The cartoon in 6B should be in this order as well. Thanks for bringing this issue up. We have adjusted the figure and text placing the schematic time-lines in proper order.

24. 337: it is not clear what the 'direct exposure...' is trying to tell us. Can this be made more explicit? The direct exposure means that the fungal cells are in contact with the culture media at the edges/border of the 3D skin model (see schematic diagram). Hence, fungal cells are in direct contact with 10% FBS, facilitating the observed filamentous growth. The inability of the put mutants to invade the skin model should be evaluated at the center of the artificial epithelium where there is likely a local increased concentration of proline stemming from the proteolytic activities associated with fibroblasts and keratinocytes.

25. 340-346: Here proteins with high proline content were used to ask if they could be induce transcription of PUT1 or PUT2 RNA and protein. This experiment is designed only to test the role of these proteins to induce utilization of nitrogen, as glucose is included in the medium. Given that these proline-rich proteins need to be lysed by proteases before they can be imported, and since no import pathways were tested, the results appear to tell us that mucin is more readily digested to peptides that contain proline-but why that is the case is not clear and how it relates to proline utilization is also not clear. We thank the reviewer for raising this important point. First, we monitored protein not mRNA levels. We will adjust the text to provide better context for this experiment. Briefly, these experiments were initiated as we were perplexed as to why the wildtype cells took such a long time (14 days) to fully invade the collagen matrix (Fig. 4B); we naïvely assumed that fungal cells would secrete proteases to degrade the collagen and assimilate the liberated proline. In going forward, our experimental strategy was to incubate various proteins with a dense culture of cells in HBSS medium (pH 7.4) supplemented with low glucose (3.8 mM) and lactate (0.83 mM). This condition mimics interstitial fluid, where most broad range proteolytic enzymes are inactive or at least operating suboptimal. The results were clear; with the exception of mucin, the proteins did not stimulate Put1 or Put2 expression. We conclude that host-dependent processes play an important role on the release of the amino acids/peptides from these high-proline content proteins (see line 531-553 for discussion). The capacity of mucin to efficiently induce Put1 expression is interesting since mucin is abundant in the gut where systemic infections are thought to originate. It is important to be cautious here, we used a commercial mucin preparation (Sigma, 2 batches) that may contain degradation products, e.g., proline-rich peptides, that can easily be assimilated by C. albicans. Put1 expression is an excellent readout for proline uptake since its expression responds tightly to the presence of proline derived from exogenous supply or from intracellular conversion (Fig. 2D, S2A, S2B).

26. 363-369 An alternative is that Put3 induces different proteins important for growth. We included this possibility in the revised text.

27. 379-380-the conclusion for this paragraph is somewhat of an overstatement as there is no analysis of the degree to which proline utilization is a predictor of virulence. It simply shows that put mutants affect the ability to survive in neutrophils. We have adjusted the text.

28. Discussion: The statement that "S. cerevisiae" evolved in high sugar environments is debatable. The natural niche could well be forest soil and tree bark, or insect/wasp guts with arguably little glucose around. The reviewer is correct, *S. cerevisiae* can be isolated from diverse environments with variable sugar contents, but it is the capacity to deal with high sugar environments that makes this yeast stand out in comparison to *Candida* spp. The unique attribute of *S. cerevisiae* have been exploited and truly benefited humankind in making alcohol and bread. We have amended the text to state this more accurately.

29. 469-470-how strong is the 'correlation' between the ability to utilize proline and virulence? Given that different mutants had different effects in different models, this seems like a very loose 'correlation'; it would be good to have some quantitative measures to make this claim. We have used directed genetic approaches to determine whether a gene/protein is essential for virulence by testing them in currently available infection models. It is important to note that all virulence assays provided a consistent and clear read-out, namely that the inability to catabolize proline significantly reduced the expression of virulence characteristics. Presumably the differences we report are due to the specific nutrient composition (proline and metabolites feeding into the proline catabolic network) and physical parameters intrinsic to each model. In fact, the expression of virulence factors (i.e., hyphal growth) can significantly differ in different organs within a same mouse model (Lionakis et al., 2013) and that virulence outcomes can change depending on mouse background. We fail to see how this can be viewed as loose. This has not been shown before. Please refer to our response to major point 6.

30. 500: Was the experiment was done in larvae, and not in adult Drosophila? Fig 5 legend says flies and shows a picture of a fly and larvae are only mentioned much later in the text. These experiments were performed using adult flies. We now include a reference regarding the levels of arginine in hemolymph in both larvae and adult Drosophila (Priyankage et al., 2012; Anal Chem).

31. 512:Why is it presumed that proline accumulates in the mitochondria in put1 mutants? How strong is the presumption? Despite a great deal of efforts in many labs, the mechanism of proline transport across the mitochondrial membrane is not known. What has been shown in mammalian and plant systems is that proline can readily enter and accumulate in mitochondria where it is catabolized. (<https://link.springer.com/article/10.1007/s00425-005-0166-z>; <https://www.sciencedirect.com/science/article/pii/0003986177902089>). Our presumption that proline accumulates in the mitochondria is based on our finding that proline inhibits mitochondrial respiration when Put1, catalyzing the first oxidation reaction, is absent.

32. 539: why are MMPs important for digestion of collagen? This is not clear at this point of the Discussion. In mammalians cells, some secreted MMPs have collagenase activity (e.g., MMP-1) that degrade proteins comprising the extracellular matrix, which releases proline. We emphasize this since the 3D skin model is comprised of dermal fibroblasts and keratinocytes that are known to secrete MMPs (Ref. 69).

33. 574: Concluding sentence of this paragraph seems unsubstantiated. There are at least two defects in put2 strains-hyphal growth and growth in general, presumably because of P5C accumulation. See response to point 21. Proline-induced filamentous growth is dependent on its catabolism, which activates Efg1 and consequently the hyphal growth program. However, there are many potential cues in hosts that could induce hyphal growth in situ. Our finding that strains unable to catabolize proline do not filament, indicates that proline is a key modulator of virulence.

34. Fewer abbreviations would make the manuscript easier for non-experts to read. For example, P5C is not defined in the abstract. Furthermore, if an abbreviation is not used more than 3 times, it is not necessary to provide it (e.g., mammalian proteins in the last paragraph). We have adjusted the text.

typos:

1. 82: should read 'is restricted to the mitoch...' THANKS!

2. 102-103: should read 'to evade macrophages' THANKS!

3. Fig. S4F is mislabelled as Fig. S4G. THANKS!

**Referees cross-commenting**

Overall, we stand by our initial assessment of the study. However, we were not aware of previous studies that investigated proline utilization in yeasts, as noted by Rev # 2 (<https://onlinelibrary.wiley.com/doi/epdf/10.1002/yea.1845>). The current study suggests that using proline as an energy/carbon source is more wide-spread, beyond pathogenic yeasts. Further, the C. albicans strain they used for this study (ATCC 10231) was apparently unable to grow on proline in the quoted paper. In light of this, we think the authors should reference this study, tone down the claims about the clear correlation of pathogenicity and proline utilization, and address this apparent discrepancy with the indicated Candida albicans isolate. We note that our review considered this a paper mostly of interest to specialists. Although other non-pathogenic fungi have been shown to use proline as pointed out by Reviewer 2, this metabolic attribute has not been previously tested in members of the pathogenic *Candida* spp. complex. We have included the reference and included a statement that many fungi, isolated from diverse environmental niches, can use proline as a carbon source.

Reviewer #1 (Significance (Required)):

1. The advance in this paper is conceptual for the proline utilization connection to virulence in a range of species and technical for the in vivo microscopy. Limitations are that the conceptual advance is based only on qualitative work in figure 1 and that the animal studies do not provide a conceptual advance, although the technical advance of in vivo visualization of kidney tissue is impressive and (to the knowledge of this reviewer) quite new as the only prior work was in mouse ears. In response to the reviewer’s comment regarding Fig. 1, although it is qualitative, it is very reproducible. We even tried several clinical isolates of *S. cerevisiae* and observed consistent behavior to the standard laboratory strains (i.e., they do not grow on SP medium where proline is used as sole carbon/nitrogen/energy source). We tried to quantify growth of all strain in liquid SP medium at 30 ^o^C using a TECAN microplate reader, but then the results show very erratic reading among species (and replicates) as each behaves differently; *C. tropicalis*, *C. krusei*, and *C. parapsilosis* form pseudohyphae and clump readily, while *C. albicans* forms hyphae and pseudohyphae.

2.The work fits well as an extension of the body of work from the corresponding author's lab with additions from the labs with expertise in models of infection.

3. People interested in yeast metabolism and pathogenic yeast virulence will be the audience for this paper and as written it is for a specialized audience interested in pathogenic yeast metabolism and, perhaps, (although not mentioned at all in the text) for those who want to try PUT gene products as new drug targets.

This was actually mentioned in the last paragraph of the discussion (line 581-582).

4. Reveiwer expertise is in pathogenic yeast biology and yeast metabolism. Little expertise in high tech microscopy. 

Reviewer #2 (Evidence, reproducibility and clarity (Required)):

The study is part of the continuous work by the authors to dissect the mechanism of utilization of proline as a carbon source in Candida spp. In particular, this work shows that the inability to process proline leads to accumulation of the toxic intermediate P5C and subsequent inhibition of mitochondrial respiration and toxic effect on the cells. Furthermore, the study demonstrates that proline utilization is important for C. albicans kidney colonization. The experiments are meticulously designed and the study adds to the overall understanding of the metabolic utilization of proline as a carbon source and its potential relevance for infection.

I find this work interesting, but the role of Put1 and Put2 in proline utilization is not particularly novel. The novelty here is the subcellular localization of the two proteins. Also, the importance of proline utilization for infection is unclear. The host-pathogen interaction assays are ambiguous as each assay gives different result. Lastly, the authors try to generalize the importance of use of proline as a energy source by other Candida spp.. This is not very surprising, given that it has been reported previously by others (example DOI: 10.1002/yea.1845) and that many pathogenic or closely related to C. albicans species use various amino acids, not only proline, as a carbon source.

Yes, as reviewer 2, we are not surprised that many of the pathogenic members of the Candida spp. complex are able to use proline, but this needed to be checked. The fact that proline can be used as a sole carbon/nitrogen/energy source clearly set them apart from the paradigm yeast *S. cerevisiae*. A major question is what amino acids are important in the context of the host? To assess this, we have used mutations that specifically block proline utilization. Our past studies demonstrating that proline catabolism is rapidly activated in *C. albicans* cells phagocytized by macrophages indicates that proline is present in the phagosomal compartment. Furthermore, put mutations clearly affect virulence in flies and murine systems. We are at a loss to understand why the reviewer believes that our data, which consistently shows that proline catabolism is important, is ambiguous.

The expectation that all three mutant strains, i.e., *put1*, *put2* and *put3,* would behave identically in the different infection models reflects an unnuanced view of how infection works. In fact, differences considered trivial such as the use of mouse background can have a profound effects on virulence. Consequently, it is striking how the diverse infections models consistently and unequivocally demonstrate that proline catabolism affects virulence. Also, it should be appreciated that we are not testing mutations affecting proteins with many overlapping functions, where it may be appropriate to challenge claims as to their direct role in virulence. Here we tested mutants that lack the enzymes that catalyze proline utilization. A more reasonable expectation is that the virulence is commensurate to the specific nutrient composition of model systems (as asked by reviewer#1), which can fluctuate among models (see our response to the major comment 6 of reviewer 1). As it is not practical to precisely test the proline levels in the models, we have worked to identify and focus on critical phenotypes that can be analyzed in vitro. Our findings provide the basis for understanding the virulence and growth properties of the mutants in the context of the complex infection models.

Moreover, the authors take C. albicans as an example to demonstrate the role of PUT in invasion and infection. Proline is known stimulus for hyphal growth in this species, but many other Candida spp., including C. auris, do not filament. So how, aside from supporting growth, proline is linked to infection in these species? I think the authors oversell the importance of proline in Candida spp. pathogenesis and should tone this part down or remove completely. A new story that validates the importance of PUT in non-albicans species can bring clarity to why and where proline is critical for survival and infection. The fact that proline supports growth in the host environment is one of the critical aspects of our work. The lack of appreciation for this finding represents a common misconception in infection biology. It is not just the ability to gain access to a host and initiate an infection that counts, it is equally important to sustain growth and to thrive within the host. Thus, the adaptation to the host environment is critical. Here we document that proline catabolism not only initiates but sustains an infection acting as a critical carbon/energy source. The inability of the *put1* and *put2* mutants, which are sensitive to proline, to grow and infect multiple models clearly suggests the substantial quantity of proline is accessible. Also, we have constructed *C. glabrata* (Fig. S1C) and *C. auris* (not shown) strains that lack the ability to catabolize proline, and are currently characterizing the virulence properties of these strains. This is out of the scope of the present study.

Major comments: I am not convinced by the data that proline is important to initiate infection. Candida infections of the kidney occur only at late stages of sepsis. The authors need more compelling data to prove that proline is important for infection in the host. Again, not sure why there is such skepticism here, regardless of whether kidney infections occur late, the fact that in contrast to WT, we do not observe *put* mutants filamenting, clearly suggesting that the capacity to catabolize proline plays a role in the expression of virulence characteristics of *C. albicans*. Based on our findings using IVM, which provides 3D information, we can at least conclude that a single isolated *C. albicans* cell can initiate hyphal growth, initiating a point of infection. In addition, our newly added whole human blood data suggests that proline catabolism is required for survival in the blood; human blood contains high amount of proline, arginine, and ornithine that are all catabolized via the proline catabolic network.

Minor comments: I find the manuscript difficult to read and the discussion part is overly long. Some streamlining and adding a bit more explanation for the rationale of each experiment will make the work easier to follow. Some language/style needs refining as well. We have attempted to take this critique into account during the revision of the manuscript and have streamlined the text and added explanations regarding the rationale underlying our experimental approaches.

**Referees cross-commenting**

In this manuscripts the authors clarify the cellular compartmentalization of steps in proline catabolism. However, it is not novel that proline is a valuable carbon source. The role of proline utilization for establishing or progression of infection remains ambiguous even after the authors provide different in vivo results. The overall significance of the study is limited. Please refer to our comments below. We do not understand that the reviewers apparently question the obvious role of proline utilization facilitating virulence.

Reviewer #2 (Significance (Required)):

The strengths of this study are in the experimental design and variety. The data is well presented and visualized. The limitations are as pointed above - I find it especially difficult to figure out where, in a real infection scenario (e.g. breach of the gut barrier and entry into the bloodstream) proline will be the primary energy source. To me the significance of this work is minor. *C. albicans* is the primary human fungal pathogen placed under the “Critical Priority Group” by WHO and yet our understanding of nutrient assimilation in this fungal pathogen is only a fraction of what is known in the model yeast *S. cerevisiae*, which has proven not to be the best paradigm for understanding the regulatory circuits operating in human fungal pathogens. This manuscript, as well as other recent publications, have revisited and corrected earlier assumptions regarding *C. albicans* growth, providing novel information that reflect important regulatory differences specifically relevant to the life of *C. albicans* in the host. For example, had it not been for the recent findings (Ref. 10, 18, 31) that show that proline utilization in *C. albicans* is not subject to nitrogen catabolite repression (NCR) and that glucose represses mitochondrial function, the perception in the field would remain that *C. albicans* cannot utilize proline as a carbon and/or nitrogen source in the presence of a “preferred” source of nitrogen, which is applicable in the blood that contains high concentrations of possible sources of carbon and nitrogen. Furthermore, the low but constitutive expression of Put2 and the tight highly responsive Put1 expression in response to proline (Fig. 2D, S2A, S2B), suggest that *C. albicans* is well equipped to productively anticipate proline availability depending on the host status, entirely consistent with its “opportunistic” character. The many incorrect and previously held assumptions regarding *C. albicans*, uncritically propagated in several influential reviews, likely have hampered efforts to develop novel antifungal therapies. We do not understand, nor accept the view that a more precise understanding of the proline catabolism is incremental.

The type of question raised by the reviewer is exactly what we hope to achieve in the future but to get there we have to have correct assumptions in place, and this is only possible if we have a more thorough understanding of the regulatory mechanisms driving proline utilization in *C. albicans*. The idea that certain proteins are refractory to degradation by *C. albicans* suggest that other external factors are triggering the release of amino acids from these proteins. This work however, suggest that proline is likely accessible in the gut due to the presence of proline-rich proteins like mucin (Fig. S5A/B).

Reviewer #3 (Evidence, reproducibility and clarity (Required)):

The manuscript of Silao et al. describes an in-depth investigation of the role of Put1 and Put2 enzymes in proline catabolism and virulence in Candida albicans. This is an extension of previous work in this system. The basic biochemistry and genetics are solid and support the role of these enzymes in the proposed pathway and provide evidence that the build up a toxic intermediate in the absence of Put2 is likely involved in the poor growth of the strain when proline is the only carbon source. Note that we observe the toxic effects of proline even when it is not the sole carbon source, however, and importantly, toxicity is dependent on mitochondrial function, which is repressed by high levels of glucose. Proline toxicity is observed when glycerol/lactate are present as carbon sources *in addition to* proline. Under these conditions, mitochondria are not repressed and exogenous proline impairs growth, particularly evident in *put2* cells that accumulate the toxic intermediate P5C.

The conclusions regarding its role in virulence are less convincing, particularly the data derived from the collagen invasion assay, the ex vivo skin model and the ex vivo/in vivo imaging. The survival and fungal burden assays support a modest role in virulence and a modest reduction in infectivity (although the presented data for survival does not have statistical significance data reported for the kaplan analysis. See below for response regarding collagen assay. We have included the significance values derived from Kaplan analysis in the revised Fig. 5B.

The manuscript is clearly written. The methods are well described.

**Referees cross-commenting**

I remain unconvinced of the broad significance of the advances and stand by my assessment that this is for the most part a reasonable study but does not move the field forward. The novel technical aspects are either extensions of previous in vivo imaging or are not well controlled (collagen invasion assay)s. See below for response.

Reviewer #3 (Significance (Required)):

This is a detailed study of an area that is fairly mature and thus will be of interest to those in the field but does not represent a large advance and is thus truly incremental. See below for response.

Major limitations of the work are as follows. First, the collagen invasion assay may be flawed. The recovery media is made with DMEM which is a medium that lacks proline and is fairly stringent. Control experiments need to be done to be sure that the mutants grow in the recovery medium. Second, the data from the RHE model are hard to interpret since so few cells are present in the tissue. It is hard to see if there are few filaments of if there are just too few cells to assess in the tissue. Third, in vitro experiments assessing the filamentation of the mutants in the medium in which these assays are preformed need to be done as controls. Candida albicans filaments in many conditions such as tissue culture medium. Spider medium is a strong inducer of filamentation but is very different than in vivo/ ex vivo conditions. Related to the collagen invasion assay, there is a misunderstanding. The reviewer appears to confuse the *put* mutations with proline auxotrophy. The *put* mutants are proline prototrophs and can synthesize proline as they possess a full repertoire of biosynthetic enzymes. In contrast, the *put* mutants cannot utilize proline to obtain nitrogen or energy. In fact, the presence of excess proline imposes toxicity to the *put* mutants. There are three possible sources of proline. 1) PureCol EZ Gel is a ready-to-use collagen solution that forms a firm gel when warmed to 37 °C. It contains purified Type I bovine collagen (5 mg/ml) dissolved in DMEM/F-12 medium, which has multiple amino acids, including a substantial amount of arginine. 2) The recovery medium DMEM supplemented with 10% FBS. The presence of FBS provides amino acids and induces filamentous growth. As the reviewer points out, C. albicans grows in this media and exhibits filamentous growth. 3) The proteolytic breakdown of collagen is expected to liberate proline. Consequently, the poor growth of the mutants clearly demonstrate the importance of proline catabolism. Also, the fact that we recovered *put* mutants surviving on top of the collagen (Fig. 4B, inset) suggests that they remain viable but simply are unable to efficiently invade the collagen. Consistently, microscopic inspection of the wells of the *put* mutants showed extremely few or even complete absence of invading cells in the recovery medium. We will adjust the text and provide a more detailed description of the experimental set-up. In summary, the main concern of the reviewer with respect to lack of proline is not relevant.

Regarding the 3D-skin model, equal numbers of fungal cells were applied on top of the RHE. To avoid overgrowth, only low numbers (100 C. albicans cells) can be applied for the WT strain, and consequently for all other strains. In contrast to WT, which clearly proliferates, the apparent low level of *put1* and *put2* cells at the center of the 3D skin model is the consequence of poor growth. The upper layer of the RHE consists of stratified keratinocytes. To grow, WT fungal cells obtain proline either directly from the keratinocyte, from secreted proteases that liberate proline from keratin (proline not as abundant in keratin as in collagen, the main component of the dermis), or from the medium that basolaterally feeds the RHE. At the border of the model leakage from the medium can occur. Our results, showing poor growth of the mutants in the center of the 3D-skin model, entirely consistent with the collagen plug experiments, indicates that proline catabolism plays a determinant role to enable invasive growth.

Lastly, the imaging experiments are highly problematic. First, reference must be made to previous ex vivo imaging reported by the Lionakis lab in 2013. Second, the number of cells imaged is so low that there is no power to make any conclusions. At 24 hr, the mutants may be delayed in filamentation or they may be delayed in establishing infection. There is no way to know what is causing the apparent lack of filaments. This technique as presented is not any higher resolution than traditional histology and in fact histology would provide a more convincing case for reduced filamentation.

These considerations significantly reduce the overall significance of the work.

I work on Candida albicans.

We thank the reviewer for highlighting the beautiful study by Lionakis et al which document the host response, specifically the role of macrophages in mitigating *C. albicans* infection of the kidney. However, the reviewer apparently failed to recognize that their method is completely differed from ours. Lionakis et al. performed ex vivo imaging of kidney slices using regular confocal imaging, and the authors express an awareness regarding the limitations of this approach. In fact, these authors even state in their discussion that intravital microscopy should be pursued in the future to further investigate Candida-macrophage interactions in the kidney. Also, they point out that kidney-specific factors seem to facilitate rapid filamentous growth of *C. albicans*. In our work, we have experimentally addressed both of these astute statements. To our knowledge, our work is the first report of imaging a *Candida* cell infecting a kidney in a living mouse, which on its own is a major development and achievement considering the complexity of the kidney microenvironment. The finding that the *put2* mutant does not exhibit filamentous growth in the kidney of a living mouse (24 h) is striking and strongly suggests that a substantial quantity of proline, or amino acids (e.g., arginine) that are metabolized via the proline catabolic network, is present in the kidney. This is clear based on finding that WT *C. albicans* cells respond accordingly to initiate hyphal growth. Consistent to this, it is well documented that the kidney is a major metabolic hub for arginine and proline metabolism. The work by Lionakis aligns remarkably well with our previous and current work in that put mutants exhibit greatly reduced survivability in co-culture with macrophages and do not evade these primary immune cells due to their inability to induce filamentous growth within the phagosome (Silao et al., 2019). We have adjusted the text to include a discussion that places our work in the context of the Lionakis work.

We have added a Fig. 6C showing an example of the scanned area of the kidney. Further we added the following in the revised legend to indicate that large areas of kidneys were imaged in our assessment of fungal growth and filamentation:

“Sites of colonization where localized using a spiral scan in the Las-X Navigator-module in the FITC channel. The entire area of the renal surface attached to the glass imaging window was scanned; circles highlight examples of regions of interest (ROI) exhibiting stronger and deviating fluorescence from the background. Each ROI was examined in detail using FITC, yEmRFP and autofluorescence. Scale bar, 500 µm.”

CONCLUDING STATEMENT – SUMMARY RESPONSE:

Our current work is based our previous discovery that proline metabolism provides energy to induce and support filamentous growth (PLoS Genetics, 2019). This turned out to be important since we also discovered that *C. albicans* cells depend on mitochondrial proline metabolism to evade engulfing macrophages, implicating this process as being an important virulence determinant. Consistently, using time-lapse microscopy, we subsequently found that proline catabolic enzymes are rapidly induced in *C. albicans* cells upon phagocytosis by macrophages. These results demonstrated that proline is present within phagosomes. As exciting as these findings are, they focused on a single phenotype, i.e., filamentation, and were obtained using in vitro experimental approaches. These results demanded that we pursue additional avenues to further characterize and test the in vivo relevance and merely provide a solid background for the current work.

In contrast to reviewer 2 and 3, we do not believe that our finding that proline catabolism plays such a critical role in virulence as being merely “incremental”. We also could not have foreseen that the ability to use proline as an energy source is a common feature of multiple fungal pathogens capable of causing human disease. This is conceptionally very important in that human fungal pathogens, unlike the well-studied yeast *Saccharomyces cerevisiae*, are not readily found out in nature, and thus have evolved to use a similar spectrum of nutrients as host cells, including cancer cells. It is important for the fungal pathogen community to realize that regulatory switches operating in *C. albicans* are wired substantially differently to those in *S. cerevisiae*, and are likely optimized to reflect the actual condition in the host environment. The growing appreciation that diverse cancers are able to shift metabolism to exploit proline as an energy source is strikingly and fascinatingly similar to our findings with pathogenic fungi. This represents a conceptual advance in that it points to the wealth of proline stored within extracellular matrix proteins as providing a potential and significant source of energy for virulent fungal and cancerous growth.

Finally, we strongly believe it is improper to extrapolate virulence properties based on in vitro findings, and that it is essential to actually test host-microbial pathogen interactions using refined in vivo models. Our successful use of advanced intravital microscopy goes beyond traditional and accepted murine infection models and has provided us with a unique state-of-the-art vantage point. Our findings that a single *C. albicans* cell is able to initiate and establish a site of infection in a kidney within a living mouse is itself important, and coupled to the novel finding that hyphal development at sites of infection depends on the ability of the fungal cells to catabolize proline must reflect the physiological conditions in the kidney. This is not an incremental finding, and we do not understand that reviewers 2 and 3 diminish the significance of these findings. Clearly, our manuscript provides a strong foundation for more detailed and advanced studies.
