# Supplementary material for: Proline catabolism is a key factor facilitating Candida albicans pathogenicity
Source: PLoS Pathog. 2023 Nov 2;19(11):e1011677. doi: 10.1371/journal.ppat.1011677 (PMC10621835; doi:10.1371/journal.ppat.1011677)
Supplement: Attachment — Submitted filename: Response to review_ version 7g-b.docx [file ppat.1011677.s015.docx]

Response to reviewer’s comments:

1. P5C should be defined in the abstract – Done
2. C. parapsilosis seemed to grow almost as poorly as C. dubliniensis (Fig. 1A). This should be commented on. I would also soften the statements that virulence correlates with ability to utilize proline because C. dubliniensis and C. parapsilosis are virulent in humans. – *C. dubliniensis* and *C. parapsilosis* are diploid members of the pathogenic Candida species complex. Existing literature suggest that *C. dubliniensis* and *C.* *parapsilosis* are not as virulent as *C. albicans* (Moran et al., 2012 PMID: 21904553; Andes et al., 2012, Clin Infect Dis). In fact, this is reflected in epidemiological data of species associated with candida infections (Turner and Butler, Cold Spring Harb Perspect Med. 2014 Sep 2;4(9):a019778) and the lower mortality rate associated with *C. parapsilosis* compared to *C. albicans* (Andes et al., *Clinical Infectious Diseases*, Volume 54, Issue 8, 15 April 2012). Also regarding *C. parapsilosis*, this fungus has unique mitochondrial functions with parallel oxidases, some that are not coupled to creating a proton gradient, and thus may be less able to obtain energy from proline (Toth et al., 2019; PMID: 30814115). Under the growth conditions we have used, *C. parapsilosis* may use proline as energy source less efficiently, however, in certain microenvironments, e.g., phagosomes, the alternative oxidases may be less active resulting in tighter coupling. Regarding *C. dubliniensis*, as stated by Moran et al., “Taken together, these data suggest that *C. dubliniensis* is in the process of undergoing reductive evolution and may have become adapted for growth in a specialized anatomic niche.” Perhaps proline is less abundant in this specialized niche, i.e., oral cavity.

Line 132: Changed text to ”considered to be less virulent” from ”considerably less virulent”

Line 523: Changed to “*The ability to use proline correlated with established virulence characteristics of the Candida species, exemplified by comparing the growth of the two closely related species C. albicans and C. dubliniensis, C. albicans considered to be more virulent (66)*”

3. Line 184. Define SGL – Done
4. Fig 2E. Need statistical comparison between Am and each of the proline isomers/analogs to show which are significantly different from baseline, especially for the put3 mutant.  This is important because it is not clear whether there are any significant changes in Put2GFP expression in the put3 mutant (unlike what is stated in the text) – Done. We analyzed the intrastrain effect of analogues by one-way ANOVA followed by posthoc test. Statistical significance is now indicated.
5. Fig. 3 and 4. The panels should be arranged so that they can be read from left to right and then top to bottom.  For example, panel B should be placed to the right of panel A in the same row – Done
6. Fig. 3E shows that the presence of proline causes the same drop in O2 consumption in the put1 cells as in the put2 cells. However, proline has a much greater inhibitory effect on the growth of the put2 mutant than the put1. These findings do not appear to support the conclusion that the growth inhibitory effect of proline in the put2 mutant is due to a block in respiration (lines 274-6). This conclusion also needs to be modified in the Discussion (586-590). Also, statistical comparisons between the different strains of C. albicans needs to performed and indicated for Fig. 3E. We will adjust the title of the section in the results to “**Proline inhibits growth of proline catabolic mutants**” and in the legend 3E. Since *put1* mutations affect the 1^st^ catabolic step, proline itself must be impairing mitochondrial functions. By contrast, *put2* mutations result in the accumulation of the toxic intermediate P5C. Note that Put1-RFP is robustly expressed in a *put2* strain when proline is supplied (Fig. S2C) suggesting that proline is readily converted to P5C. As we mentioned in our response to the first round of reviews (Review Commons), previous work by Nishimura et al. (PMID: 22698729) convincingly showed that P5C targets respiration, hence the original “respiratory block” in the header. The IC50 for P5C determined by these authors is very low, which is consistent to the very low levels of proline (78 μM) required to inhibit *put2* (Fig. 3F). With respect to the drop in O_2_ consumption, although the resulting O_2_ consumption for both *put1* and *put2* look the same in the presence of proline, the baselines (without proline) are different. The *put1* strain, even in the absence of proline, shows a significant reduction in O_2_ consumption relative to wildtype, presumably because it affects the basal function of the ETC (see the blue asterisks derived from one way ANOVA analysis with multiple comparison made on the no proline baseline). Consequently, the addition of proline to *put1* resulted to a 43% drop in the O_2_ consumption rate while it is 51% in *put2*. The *put2* mutation does not have the same effect on basal respiration presumably because Put2 is in the matrix while Put1 is predicted to associate with complex II of the ETC at the inner mitochondrial membrane (Hancock et al., 2016, PMID: 26660760). The precise inhibitory mechanism of proline remains to be elucidated, and as we mentioned, to study this we are starting to characterize various GFP-tagged respiratory complex components in *put1* mutants and in strains co-expressing Put1-RFP to check for possible interactions. The results are out of the scope of this current work.
7. Fig. 3G. If phloxine B is a measure of viability, one would expect that the colonies with phloxine B staining would be smaller than those that don’t stain because nonviable cells obviously don't proliferate. Why are the colonies of the put1 and put1/2 mutants larger than those of the WT cells, especially in YPD, even though they are phloxine B positive? This observation raises the concern that phloxine B staining may not be a good measure of viability. – The comment is correct if there is a simple temporal relationship with phloxine B accumulation and colony growth. However, on YPD, the toxic effects of proline is complex due to glucose repression of mitochondrial function. Proline toxicity results only after cells within the developing colony are relieved of glucose repression, which occurs faster at elevated temperature (37 ^o^C). WT colonies can expand in all directions, including upwards, due to not being susceptible to proline toxicity, whereas the *put* colonies expand primarily at the colony edges (glucose repressed). Consequently, the relationship with phloxine B and colony size is not simple.

Note that the growth rates of WT and *put1* in liquid YPD (Fig. S4G) are similar until cells reach the saturated phase, accompanied by the relief of glucose repression. At this point, OD_600_ measurements of *put1* cultures become highly erratic due to aggregation of cells, which contain higher number of propidium iodide (PI) positive cells (Fig. S4I). Consistently, the *put1* mutant colonies growing on YPD are composed of highly aggregated cells, a well-characterized stress response (correlates with the more wrinkled colony appearance). The toxicity of proline in *put1* cells under glucose-limiting conditions is likely due to the inhibition of mitochondrial function and reduced capacity to generate ATP. Interestingly, the growth phenotype exhibited by *put1* and *put1 put2* mutants on YPD appear to overlap with some of the reported phenotypes of ETC complex mutants (Zhang et al., 2018, FGB; PMID: 29146491). We are continuing to investigate the growth inhibitory effects of proline in cells lacking the ability to catabolize it.

Although we remain convinced that phloxine accumulation reflects cell death, we have not pinpointed the precise mechanism of proline toxicity. Thus, we have adjusted our terminology to use **vitality** rather than **viability.** This includes the possibility of phloxine positive cells being merely metabolically inactive and thus unable to pump out the dye (Kwolek-Mirek, et al., FEMS Yeast Research., PMID 25154541; Carmona-Gutierrez et al., 2018, Microbial Cell).

8. Fig. 5 A and B. Need statistical analysis of the survival curves (using Kaplan Meier statistics).  Is the survival of flies/mice infected with the put1 mutant significantly different from the put2 mutant?  This is stated in the manuscript, but likely not supported by statistical analysis. For flies, using the Bonferroni-corrected α value, there is no significant difference between the virulence of *put1* and *put2* mutants, both strains exhibit greatly reduced virulence. For the mice, as there are 3 curves per strain, area under the curve (AUC) was determined from each curve and the AUCs analyzed by one-way ANOVA followed by Tukey’s posthoc test. There was no significant difference between put1 and put2. We have corrected the text accordingly.
9. Fig. 5C. The organ fungal burden of mice infected with put1 and put2 mutants should be compared statistically to evaluate the possibility that the put1 mutant achieves lower organ fungal burden than the put2 mutant. We thank the reviewer for this comment. We performed a localized comparison of fungal burden between *put1* and *put2* using student t-test, and interestingly we found that in certain organs such as spleen and brain, *put1* is significantly lower compared to *put2*, whereas in the kidney and liver, they are statistically similar. We adjusted the text to reflect this result.
10. Lines 395-6. Should compare the median survival, not mean survival because median survival is less affected by outliers. Thanks for this! We adjusted the text in accordance to this recommendation. Median survivals of mice infected with *put1* and *put2* were undefined since >50% of subjects survived. We compared the survival curves of *put1* and *put2* and found no significant difference between the two. In addition, there was no significant difference in the median survival (in days, d) of wildtype (8.5d, 7.5d, 7d) and *put3* (9d, 7d, 7d) obtained from 3 independent experiments. Similarly, the median survival of flies infected with *put1*, *put2*, and *put1* *put2* were undefined since >50% of flies survived, while wildtype and *put3* showed a median survival of 3 and 6 days, respectively.
11. Fig. 5D. The magnification in the lower panels is not sufficient to see any organisms in the kidneys infected with the put1 and put2 mutants, and the possible filamentation defect of the put3 mutant is also not evident. The sections show mainly the renal pelvis. Were there visible lesions in the renal cortex? If so, how did they look? Since the PAS staining only displays a thin layer in the middle of the kidney, this method did have limitations in representing the fungal burden. However, PAS staining can truly reflect the colonization position of fungi and the basic conditions of organs at the time of sampling. In the kidneys infected with the *put1* or *put2* mutants, the fungal burden was nearly 10 times lower than that of the other two groups, which is the major reason why visible fungal cells in these two groups look very sparse and not obvious. Indeed, strain infections of the renal pelvis could be clearly visualized at a higher resolution. Here is an example of the PAS staining results in kidney infected with the *put1* mutant, the fungal cells (purple) are clearly displayed in the area of red circle.

The PAS staining can only show the picture at a single time point. It is impossible to track the dynamic change of fungal morphology. Moreover, the PAS staining results are limited to a single plane, it is difficult to count the number of cells in either yeast or hyphal morphologies. Based on the fungal load statistics and PAS staining results, we speculate that the *put3* mutation results in a higher proportion of yeast-like rather than filamentous cells.

Our PAS staining results suggested that the fungal colonization was mainly concentrated in the renal pelvis. We mainly intercepted the vicinity of the renal pelvis when displaying the staining images. The fungal colonization in the renal cortex was less distinct than that in the renal pelvis. Moreover, we observed that kidneys infected with WT or *put3* mutant had significantly higher levels of lesions with inflammatory cell infiltration in the renal cortical areas and few numbers of *Candida* cells that could be visualized (marked by red arrows).

12. The conclusion that “especially Put2” is require for virulence is not supported by the data because there were no statistically significant differences between put1 and put2 mutants in the in vivo infection models.  The in vitro models clearly did not mimic the in vivo models because deletion of PUT3 had a much greater effect in vitro than in vivo. We agree and modified discussions previously alluding to Put2 being more important than Put1. However, as we mentioned in the earlier response to reviewers, the fact that proline is growth inhibitory and affects hyphal formation in cells that cannot catabolize it efficiently complicates interpretations. We also need to adhere to the most basic premise that mice challenged intravenously die not due to Candida cells themselves, rather due to progressive sepsis (Spellberg et al., PMID: 15962230) correlating with the induction of host responses activated by recognition of immunostimulatory PAMPs exposed during hyphal growth.

Currently, we are unable to precisely differentiate the cause of reduced hyphal growth and virulence in *put1* and *put2* mutants in vivo since they exhibit overlapping and specific defects. The differences between the in vitro and in vivo models can be due to many factors, differences in oxygen, glucose, etc. Potential differences between *put1* and *put2* could be due to different mechanisms of action. For instance, while Put1 is associated with the inner mitochondrial membrane and is predicted to interact directly with components of the ETC (i.e., complex II; Hancock et al., 2016, PMID: 26660760), *put2* mutations lead to the accumulation of P5C, which inhibits respiration and also generate ROS. Note that the MIC of proline needed to inhibit *put2* is extremely low (78 μM). Regardless, proline catabolism is clearly important and a robust indicator of virulence.

13. The extended methods section should be moved from the supplemental data section into the main text. – Done, thanks!
14. Fig. 6. While the in vivo imaging data are nice, a major weakness is that the labeled strains were auxotrophic (arg- his-). It is known that even though strains with amino acid auxotrophies can appear to filament normally in vivo, these auxotrophies can interact with other mutations (e.g., the put2 deletion), leading to filamentation defects that are not apparent in fully prototrophic strains. For this reason, these studies should be repeated with fully prototrophic strains. Also, it would aid in the interpretation of the results to show data with both the WT and put2 mutant, and optimally the put1 mutant, at the same time points. In panel E, only the WT stain is shown at 4 h. Results with the put mutants should also be shown. We have already shown virulence data and kidney histology on prototrophic strains (Fig. 5), which were performed independently three times. This provides us with the confidence to state that the inability of *put1* and *put2* strains to efficiently filament in the mouse kidney is not due to the auxotrophies. As for the IVM, we started with SC5314-derived reporter strains constitutively expressing either RFP or GFP (used in Silao et al., 2019; 2020). However, the fluorescent signals, although excellent for confocal microscopy, were extremely weak for IVM. Using these strains, it was too challenging to locate fungal cells in both sedated mice and extracted kidneys. We even attempted to use the triple-tagged reporter strain (CFG433) to follow PUT expression in the kidney but the signals were even weaker due to mitochondrial localization of the enzymes. Sufficient and reproducible fluorescent signal were obtained for strain PLC096 (S. Keppler-Ross et al., Genetics 179, 705-710 (2008)), hence we continued with this strain background. To ensure that we have accounted the potential effects of auxotrophies of the parental strain for accurate comparison, we directly inactivated *PUT2* in this background. Since the *put2* strain did not filament after 24 h, we fail to see how the 4 h data will add more support of the conclusion. The goal of the IVM, highlighting the technical challenges, is to demonstrate that a single candida cell can establish itself and that proline is available for access in the kidney environment, which is reflected in the inability of *put2* to grow.
15. The statement the glutamate and glutamine are not readily converted to proline in fungal cells (lines 537-540) should be softened because the authors did not show this (by measuring intracellular proline levels) and there are alternative explanations for why glutamate and glutamine did not induced PUT enzymes. We have adjusted the text and added new data (Fig. S2D) to clarify this section. Briefly, the basis of our contention that glu/gln is not readily converted to proline is supported by the new data (Fig. S2D) demonstrating that submillimolar concentrations of proline (78 μM) readily induce Put1 expression. This strongly suggests that induction occurs at a very low threshold level. We posit that if *C. albicans* cells convert glu/gln to proline, then Put1 expression should have been induced upon the addition of the high concentrations of glu and gln to cultures (10 mM). Specifically, 78 μM is merely 0.8% of 10 mM glu/gln. Also, the basal respiratory medium used (SGL) allows for the maximal expression of the Put enzymes. Consequently, since we did not observe expression of Put1, we believe that cells did not produce significant amounts of proline from either glu or gln. The role of glutamate metabolism in ammonia production is the focus of an independent manuscript that has been submitted to PLoS Pathogens to be considered for publication.
16. The discussion in lines 604-614 are based on the presumption that proline, arginine, and ornithine are the only stimuli that induce PUT3 expression. An alternative possibility is that other factors that are present in the microniche of the phagosome are able to induce PUT3 expression. Because the investigators did not measure the amino acid levels in the phagosome, the conclusions in this section should be softened. Thanks for this comment. We assume the reviewer is referring to Put3-dependent expression of PUT1 and PUT2, not PUT3 expression. We will adjust the statement to “*suggests the presence of proline*” instead of “*clearly has significant levels of proline*” to tone down the statement. However, we would like to mention that in addition to our data, where we showed the induction of Put2 and Gdh2 (proline-dependent expression; Fig. 2D) in the phagosomes of macrophages (Silao et al., 2019; 2020), an independent work by Tebung et al. (2017; PMID: 29242833) showed that ICL1, a well-known highly transcribed fungal gene in the phagosome (Lorenz et al., 2004; PMID: 15470236) is also dependent on proline.
17. Line 636 is incorrect.  Hematogenously disseminated candidiasis is establishing a pyelonephritis (kidney infection), not candiduria, which represents simple colonization of the bladder. We get the point of the reviewer, however, we respectfully disagree. Candiduria is defined simply based on the presence of *Candida* cells in the urine, which can be due to colonization and/or infection of the lower- or upper - urinary tract; lower UTI include infections of the bladder (cystitis), urethra (urethritis), and prostate (prostatitis) in men, while upper UTI include infections of the kidney (pyelonephritis) and ureter (ureteritis). Based on our understanding of clinical literature, candiduria is often considered as a physiological marker of disseminated candidiasis in ICU patients (Kauffmann et al., 2011, Clin Infect Dis). Our statement, line 636, that we have observed the initiation of an upper UTI is based on the source of the infection being the *C. albicans* injected into the tail vein. SPF mice do not normally harbor *C. albicans* and we recovered fluorescent candida cells from urine (i.e., the injected strain). This gives us confidence that our IVM setup enabled us to visualize the onset of candiduria stemming from an upper UTI, which we will be describing in detail in a separate paper. We adjusted the text to “*thus enabling us to literally observe the establishment of candiduria stemming from fungal cells growing in the kidney (pyelonephritis) as a consequence of a systemic infection. Candiduria in ICU patients is often considered an indicator of invasive candida infections [91, 92].”*
