# Supplementary material for: Proline catabolism is a key factor facilitating Candida albicans pathogenicity
Source: PLoS Pathog. 2023 Nov 2;19(11):e1011677. doi: 10.1371/journal.ppat.1011677 (PMC10621835; doi:10.1371/journal.ppat.1011677)
Supplement: Peer review history

# Author comment: Author response to previous submission

**DOI:** 10.1371/journal.ppat.1011677.r001

13 Jul 2023

Attachment Submitted filename: 133524_1_rebuttal_2730809_rxlp3s.docx Click here for additional data file.

# Editor report: Decision Letter 0

**DOI:** 10.1371/journal.ppat.1011677.r002

**Authors:** Scott G. Filler, Alex Andrianopoulos

26 Jul 2023

Dear Professor Ljungdahl,

Thank you very much for submitting your manuscript "Proline catabolism is a key factor facilitating Candida albicans pathogenicity" for consideration at PLOS Pathogens. I reviewed the manuscript and read the three reviews that you had submitted. The reviewers appreciated the attention to an important topic and I feel that you have addressed their comments satisfactorily. However, after reading the manuscript closely, I have identified some additional issues that must be addressed. Based on the reviews, we are likely to accept this manuscript for publication, providing that you modify the manuscript according to the following recommendations.

1.  P5C should be defined in the abstract
2. C. parapsilosis seemed to grow almost as poorly as C. dubliniensis (Fig. 1A). This should be commented on. I would also soften the statements that virulence correlates with ability to utilize proline because C. dubliniensis and C. parapsilosis are virulent in humans.
3. Line 184. Define SGL
4. Fig 2E. Need statistical comparison between Am and each of the proline isomers/analogs to show which are significantly different from baseline, especially for the put3 mutant.  This is important because it is not clear whether there are any significant changes in Put2GFP expression in the put3 mutant (unlike what is stated in the text).
5. Fig. 3 and 4. The panels should be arranged so that they can be read from left to right and then top to bottom.  For example, panel B should be placed to the right of panel A in the same row.
6. Fig. 3E shows that the presence of proline causes the same drop in O2 consumption in the put1 cells as in the put2 cells. However, proline has a much greater inhibitory effect on the growth of the put2 mutant than the put1. These findings do not appear to support the conclusion that the growth inhibitory effect of proline in the put2 mutant is due to a block in respiration (lines 274-6). This conclusion also needs to be modified in the Discussion (586-590). Also, statistical comparisons between the different strains of C. albicans needs to performed and indicated for Fig. 3E.
7. Fig. 3G. If phloxine B is a measure of viability, one would expect that the colonies with phloxine B staining would be smaller that those that don’t stain because nonviable cells obviously don't proliferate. Why are the colonies of the put1 and put1/2 mutants larger than those of the WT cells, especially in YPD, even though they are phloxine B positive? This observation raises the concern that phloxine B staining may not be a good measure of viability.
8. Fig. 5 A and B. Need statistical analysis of the survival curves (using Kaplan Meier statistics).  Is the survival of flies/mice infected with the put1 mutant significantly different from the put2 mutant?  This is stated in the manuscript, but likely not supported by statistical analysis.
9. Fig. 5C. The organ fungal burden of mice infected with put1 and put2 mutants should be compared statistically to evaluate the possibility that the put1 mutant achieves lower organ fungal burden than the put2 mutant.
10. Lines 395-6. Should compare the median survival, not mean survival because median survival is less affected by outliers.
11. Fig. 5D. The magnification in the lower panels is not sufficient to see any organisms in the kidneys infected with the put1 and put2 mutants, and the possible filamentation defect of the put3 mutant is also not evident. The sections show mainly the renal pelvis. Were there visible lesions in the renal cortex? If so, how did they look?
12. The conclusion that “especially Put2” is require for virulence is not supported by the data because there were no statistically significant differences between put1 and put2 mutants in the in vivo infection models.  The in vitro models clearly did not mimic the in vivo models because deletion of PUT3 had a much greater effect in vitro than in vivo.
13. The extended methods section should be moved from the supplemental data section into the main text.
14. Fig. 6. While the in vivo imaging data are nice, a major weakness is that the labeled strains were auxotrophic (arg- his-). It is known that even though strains with amino acid auxotrophies can appear to filament normally in vivo, these auxotrophies can interact with other mutations (e.g., the put2 deletion), leading to filamentation defects that are not apparent in fully prototrophic strains. For this reason, these studies should be repeated with fully prototrophic strains. Also, it would aid in the interpretation of the results to show data with both the WT and put2 mutant, and optimally the put1 mutant, at the same time points. In panel E, only the WT stain is shown at 4 h. Results with the put mutants should also be shown.
15. The statement the glutamate and glutamine are not readily converted to proline in fungal cells (lines 537-540) should be softened because the authors did not show this (by measuring intracellular proline levels) and there are alternative explanations for why glutamate and glutamine did not induced PUT enzymes.
16. The discussion in lines 604-614 are based on the presumption that proline, arginine, and ornithine are the only stimuli that induce PUT3 expression. An alternative possibility is that other factors that are present in the microniche of the phagosome are able to induce PUT3 expression. Because the investigators did not measure the amino acid levels in the phagosome, the conclusions in this section should be be softened.
17. Line 636 is incorrect.  Hematogenously disseminated candidiasis is establishing a pyelonephritis (kidney infection), not candiduria, which represents simple colonization of the bladder.

Please prepare and submit your revised manuscript within 30 days. If you anticipate any delay, please let us know the expected resubmission date by replying to this email.

When you are ready to resubmit, please upload the following:

[1] A letter containing a detailed list of your responses to all review comments, and a description of the changes you have made in the manuscript.

Please note while forming your response, if your article is accepted, you may have the opportunity to make the peer review history publicly available. The record will include editor decision letters (with reviews) and your responses to reviewer comments. If eligible, we will contact you to opt in or out

[2] Two versions of the revised manuscript: one with either highlights or tracked changes denoting where the text has been changed; the other a clean version (uploaded as the manuscript file).

Important additional instructions are given below your reviewer comments.

Thank you again for your submission to our journal. We hope that our editorial process has been constructive so far, and we welcome your feedback at any time. Please don't hesitate to contact us if you have any questions or comments.

Sincerely,

Scott G. Filler, M.D.

Guest Editor

PLOS Pathogens

Alex Andrianopoulos

Section Editor

PLOS Pathogens

Kasturi Haldar

Editor-in-Chief

PLOS Pathogens

​orcid.org/0000-0001-5065-158X

Michael Malim

Editor-in-Chief

PLOS Pathogens

orcid.org/0000-0002-7699-2064

***********************

Reviewer Comments (if any, and for reference):

Figure Files:

While revising your submission, please upload your figure files to the Preflight Analysis and Conversion Engine (PACE) digital diagnostic tool, https://pacev2.apexcovantage.com. PACE helps ensure that figures meet PLOS requirements. To use PACE, you must first register as a user. Then, login and navigate to the UPLOAD tab, where you will find detailed instructions on how to use the tool. If you encounter any issues or have any questions when using PACE, please email us at figures@plos.org.

Data Requirements:

Please note that, as a condition of publication, PLOS' data policy requires that you make available all data used to draw the conclusions outlined in your manuscript. Data must be deposited in an appropriate repository, included within the body of the manuscript, or uploaded as supporting information. This includes all numerical values that were used to generate graphs, histograms etc.. For an example see here: http://www.plosbiology.org/article/info%3Adoi%2F10.1371%2Fjournal.pbio.1001908#s5.

Reproducibility:

To enhance the reproducibility of your results, we recommend that you deposit your laboratory protocols in protocols.io, where a protocol can be assigned its own identifier (DOI) such that it can be cited independently in the future. Additionally, PLOS ONE offers an option to publish peer-reviewed clinical study protocols. Read more information on sharing protocols at https://plos.org/protocols?utm_medium=editorial-email&utm_source=authorletters&utm_campaign=protocols

References:

Please review your reference list to ensure that it is complete and correct. If you have cited papers that have been retracted, please include the rationale for doing so in the manuscript text, or remove these references and replace them with relevant current references. Any changes to the reference list should be mentioned in the rebuttal letter that accompanies your revised manuscript. If you need to cite a retracted article, indicate the article’s retracted status in the References list and also include a citation and full reference for the retraction notice.

# Author comment: Author response to Decision Letter 0

**DOI:** 10.1371/journal.ppat.1011677.r003

3 Sep 2023

Attachment Submitted filename: Response to review_ version 7g-b.docx Click here for additional data file.

# Editor report: Decision Letter 1

**DOI:** 10.1371/journal.ppat.1011677.r004

**Authors:** Scott G. Filler, Alex Andrianopoulos

11 Sep 2023

Dear Professor Ljungdahl,

We are pleased to inform you that your manuscript 'Proline catabolism is a key factor facilitating Candida albicans pathogenicity' has been provisionally accepted for publication in PLOS Pathogens.

Before your manuscript can be formally accepted you will need to complete some formatting changes, which you will receive in a follow up email. A member of our team will be in touch with a set of requests.

Please note that your manuscript will not be scheduled for publication until you have made the required changes, so a swift response is appreciated.

IMPORTANT: The editorial review process is now complete. PLOS will only permit corrections to spelling, formatting or significant scientific errors from this point onwards. Requests for major changes, or any which affect the scientific understanding of your work, will cause delays to the publication date of your manuscript.

Should you, your institution's press office or the journal office choose to press release your paper, you will automatically be opted out of early publication. We ask that you notify us now if you or your institution is planning to press release the article. All press must be co-ordinated with PLOS.

Thank you again for supporting Open Access publishing; we are looking forward to publishing your work in PLOS Pathogens.

Best regards,

Scott G. Filler, M.D.

Guest Editor

PLOS Pathogens

Alex Andrianopoulos

Section Editor

PLOS Pathogens

Kasturi Haldar

Editor-in-Chief

PLOS Pathogens

​orcid.org/0000-0001-5065-158X

Michael Malim

Editor-in-Chief

PLOS Pathogens

orcid.org/0000-0002-7699-2064

***********************************************************

Reviewer Comments (if any, and for reference):

# Editor report: Acceptance letter

**DOI:** 10.1371/journal.ppat.1011677.r005

**Authors:** Scott G. Filler, Alex Andrianopoulos

9 Oct 2023

Dear Professor Ljungdahl,

We are delighted to inform you that your manuscript, "Proline catabolism is a key factor facilitating Candida albicans pathogenicity," has been formally accepted for publication in PLOS Pathogens.

We have now passed your article onto the PLOS Production Department who will complete the rest of the pre-publication process. All authors will receive a confirmation email upon publication.

The corresponding author will soon be receiving a typeset proof for review, to ensure errors have not been introduced during production. Please review the PDF proof of your manuscript carefully, as this is the last chance to correct any scientific or type-setting errors. Please note that major changes, or those which affect the scientific understanding of the work, will likely cause delays to the publication date of your manuscript. Note: Proofs for Front Matter articles (Pearls, Reviews, Opinions, etc...) are generated on a different schedule and may not be made available as quickly.

Soon after your final files are uploaded, the early version of your manuscript, if you opted to have an early version of your article, will be published online. The date of the early version will be your article's publication date. The final article will be published to the same URL, and all versions of the paper will be accessible to readers.

Thank you again for supporting open-access publishing; we are looking forward to publishing your work in PLOS Pathogens. 

Best regards,

Kasturi Haldar

Editor-in-Chief

PLOS Pathogens

​orcid.org/0000-0001-5065-158X

Michael Malim

Editor-in-Chief

PLOS Pathogens

orcid.org/0000-0002-7699-2064
